# Effects of Membrane Cholesterol on the Structure and Function of Selected Class A GPCRs—Challenges and Future Perspectives

**DOI:** 10.1021/acs.biochem.5c00145

**Published:** 2025-08-08

**Authors:** Marina Christofidi, Efpraxia Tzortzini, Thomas Mavromoustakos, Antonios Kolocouris

**Affiliations:** † Laboratory of Medicinal Chemistry, Section of Pharmaceutical Chemistry, Department of Pharmacy, School of Health Sciences, 68993 National and Kapodistrian University of Athens, Panepistimiopolis-Zografou, 15771 Athens, Greece; ‡ Department of Nutritional Science and Dietetics, School of Health Sciences, University of the Peloponnese, Antikalamos, 24100 Kalamata, Greece; § Laboratory of Organic Chemistry, Department of Chemistry, School of Science, National and Kapodistrian University of Athens, Panepistimiopolis-Zografou, 15771 Athens, Greece

**Keywords:** allosteric sites, cholesterol, cholesterol consensus motif, cholesterol recognition amino acid consensus motif, class A GPCR, sequencing analysis

## Abstract

Cholesterol can affect class A G protein-coupled receptors (GPCRs) function since it can modulate membrane properties, but can also bind to allosteric sites, with positions and numbers that can be general and conserved or can depend on the unique receptor and its functional state. The reliable identification and characterization of cholesterol-interaction sites, which can help toward the development of novel allosteric drugs, is challenging, but different biophysical methods, such as structural biology and molecular dynamics simulations, can be combined toward this aim. We reviewed results for 14 receptors selected based on phylogenetic relationships, functional relevance, and their established significance as therapeutic targets. The precise mechanistic interpretation of the effect of cholesterol at a molecular level is generally ambiguous for many class A GPCRs. Additionally, the experimentally observed effect of cholesterol on the function of a receptor often varies, likely due to variations in experimental systems, including cell types, lipid environments, protein constructs, or methodological differences. To elucidate the role of cholesterol in GPCR function, a robust methodological framework is required—one that integrates diverse biophysical techniques with carefully controlled experimental conditions, particularly regarding membrane composition and cellular context. Additionally, combining insights from both in vitro and in vivo studies is crucial for developing a comprehensive understanding of cholesterol’s role in GPCR modulation.

## Graphical abstract

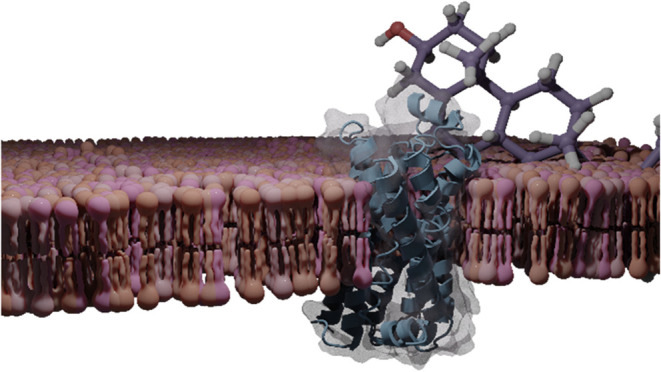

## 1 Introduction

### 1.1 Purpose of the Review

In this review, we discuss the localization and critical role of cholesterol in regulating class A GPCR function, as revealed through biophysical methods and biochemical assays. We focused on 14 representative class A GPCRs to show how different methods can be combined to explore the role of cholesterol, identify and characterize cholesterol-interaction sites, and provide structural insights that may drive the rational design of cholesterol-targeting allosteric modulators.

### 1.2 Background

#### 1.2.1 G Protein-Coupled Receptors

G protein-coupled receptors (GPCRs) account for more than 1% of the human genome. GPCRs are coupled with transducer proteins and play a crucial role in physiological processes. GPCRs traverse the plasma membrane, whose lipid bilayer is primarily composed of glycerophospholipids or phospholipids (PLs), the major class of structural lipids in biological membranes.^1^ The functional properties of GPCRs are affected by membrane lipids. The extracellular (EC), transmembrane (TM), and intracellular (IC) domains are well-defined in GPCRs. GPCRs are characterized by seven transmembrane (7TM) α-helices, linked by three EC loops (ELs 1–3) and three IC loops (ILs 1–3). Most GPCRs also feature an additional IC helix 8 (H8) connected at the end of TM7 and a disordered C-terminal tail (C-tail).

It is widely accepted that GPCRs are highly dynamic proteins that can adopt multiple distinct conformations, depending on the ligands, signaling transducers, and lipid environment, through a conformational selection mechanism.^2,3^ GPCRs activate intracellular signaling pathways by coupling to heterotrimeric G proteins, composed of three subunits: Gα, Gβ, and Gγ. Upon agonist binding, the receptor undergoes a conformational change that enables interaction with the G protein complex, stabilizing the receptor in its fully active state. Within this activated complex, GDP on the Gα subunit is exchanged for GTP, triggering GPCR-mediated G protein activation and signaling. As a result of this nucleotide exchange, the G protein dissociates from the receptor; the GTP-bound Gα subunit dissociates from the Gβγ dimer, and all three subunits can be coupled with downstream effectors and can trigger downstream signaling responses. Beyond different G protein families (e.g., Gi/o, Gs, or Gq), other key transducers of GPCR signaling are GPCR kinases (GPCRKs/GRKs) and arrestins (arrs) that can transmit signals via a variety of pathways. GRKs and arrs can inhibit G protein signaling; following GRK phosphorylation of the receptor at the ILs or the C-terminus, arrs bind the active state of GPCRs, causing receptor desensitization and/or internalization and can cause arr-mediated signaling. In mammals, up to 16 G protein subtypes are expressed, classified into the four subfamilies Gs, Gi/o, Gq/11, and G12/13. Additionally, four arr subtypes (arr-2 and arr-3 being widely expressed) and seven GRKs (particularly GRK2, GRK3, GRK5, and GRK6) contribute to the regulation and diversification of GPCR signaling.^4–6^

There are over 800 distinct human GPCRs and thousands of human GPCR-ligand binding combinations. Each GPCR subfamily possesses unique structural features and ligand-binding specificities that are closely linked to the receptor’s physiological roles.^7^ For example, since signaling triggered by agonist binding is mediated through four major G protein families, this implies the existence of a “selectivity barcode” at the GPCR–G protein interface. Such a mechanism allows a vast repertoire of GPCRs to engage selectively with a limited set of transducer proteins, thereby enabling precise and diverse cellular responses.

Interactions between GPCRs and lipids represent an additional layer of specificity that contributes to the functional diversity of these receptors by influencing their structural and signaling properties.^8^ These interactions encompass not only the overall physical properties of the surrounding lipid bilayer^9–14^ —such as membrane thickness, curvature, and fluidity—but also specific, stoichiometric direct interactions between a receptor^15^ and specific lipid molecules.^15,16^

Even in natural tissues, many GPCRs have been shown to form dimers or higher-order oligomers,^8,17,18^ which can have critical roles in both physiological and pathological processes.^19^ For certain GPCRs, a dynamic equilibrium between monomers and dimers or other oligomers has been noted.^17,20,21^

#### 1.2.2 Structural Features of Cholesterol and Its Interactions in Membranes

Cholesterol is a key component of most biological membranes and plays a critical role in regulating cellular function. Its unique structural features enable it to modulate the physicochemical properties of the lipid bilayer, thereby influencing both membrane organization and the structure and function of membrane proteins. Cholesterol serves as a precursor for the biosynthesis of several hormones and vitamins. It is the predominant sterol component in the cell membrane of higher eukaryotes and is absent in microorganisms. In animal cells, however, cholesterol is a vital constituent of the plasma membrane. In the plasma membrane of mammals, cholesterol comprises 34% of the total lipid content and accounts for 80–90% of the total cholesterol content within the cell. Although plant membranes lack cholesterol, they incorporate functionally analogous sterols that fulfill comparable roles in membrane structure and receptor organization.

Several distinctive characteristics of cholesterol’s molecular structure come together to make the molecule unique from a functional standpoint. The structural features of cholesterol and their significance^22^ are analyzed below in **a–e**:

(a) The four fused rings form the common scaffold of the steroid nucleus. This tetracyclic fused structure confers rigidity and high lipophilicity to the cholesterol molecule (Figure 1A). Thus, this structural motif promotes cholesterol’s localization within the hydrophobic core of cellular membranes and facilitates interactions with lipophilic amino acid residues of membrane-associated proteins (Figure 1B).
(b) The flexible iso-octyl hydrocarbon side chain (Figure 1A) can adopt multiple low-energy conformations. This conformational flexibility enhances cholesterol’s ability to interact with the dynamic alkyl chains of membrane lipids (Figure 1B). When the rigid tetracyclic ring system cannot establish optimal hydrophobic contacts, the flexible side chain can compensate by adapting to the local membrane environment. Thus, the rigid and flexible hydrophobic moieties of cholesterol act in a complementary manner, conferring high structural and functional versatility to the molecule.
(c) The double bond between carbon atoms C5 and C6 of cholesterol is crucial for its interactions with the phospholipids that constitute the cellular membrane^23^ (Figure 1B). Due to its rigid and planar sterol ring system, cholesterol induces concentration-dependent changes in membrane fluidity, a phenomenon often described as a solvent-like effect.^24^
(d) The polar 3β-hydroxyl group (Figure 1A) equips cholesterol with a certain amphiphilicity, despite its largely hydrophobic steroid core. This hydroxyl moiety anchors cholesterol at the polar interface of the lipid bilayer, while the hydrophobic rings and iso-octyl chain align with the fatty acyl chains of membrane lipids (Figure 1B). In addition, the hydroxyl moiety can form hydrogen bonds with charged or uncharged polar amino acid side chains of membrane-associated proteins.
  Beyond anchoring cholesterol within the membrane, the 3β-hydroxyl group promotes cholesterol’s association with other amphiphilic lipids, such as sphingolipids, leading to the formation of lipid aggregates commonly known as lipid rafts.^26^ These ordered domains, including caveolae—a specialized subtype—serve as platforms for the spatial organization of membrane proteins and signaling components. Key receptors, such as tyrosine kinase receptors and GPCRs, are frequently concentrated in these regions, where the cholesterol-rich environment enhances signaling efficiency and compartmentalization.^27^ In this context, cholesterol modulates GPCR activity both through direct interactions and via colocalization with transducers and effectors.
  The compartmentalization of GPCRs within lipid rafts is further regulated by caveolin-1, a scaffolding protein found in caveolae. Caveolin-1 interacts with various GPCRs, including neurotransmitter-related receptors such as the class C metabotropic glutamate (mGluRs), mGluR type 1 (mGluR1), and type 5 (mGluR5).^28,29^ This interaction influences GPCR surface expression, raft localization, signaling, and endocytosis. Moreover, post-translational modifications—such as palmitoylation—serve as membrane anchors that promote lipid raft targeting of several GPCRs, further refining their spatial and functional regulation within the membrane.^30^
(e) Cholesterol has a smooth α-face where no methyl groups protrude, and a rough β-face, which bears two methyl groups attached to C18 and C19 quaternary carbons.^22^ The existence of two distinct faces enables cholesterol to interact differently with important biomolecules. The α-face is thought to interact more effectively with lipid acyl chains, whereas the β-face preferentially associates with membrane proteins. Notably, the rough β-face has been shown to favor interactions with β-strands while the smooth α-face is mostly associated with α-helices, highlighting cholesterol’s multifunctional character.^31^ These interactions are tailored to accommodate the cholesterol’s β-face structure through hydrophobic amino acid residues and are further stabilized by aromatic side chains. However, coarse-grained (CG) molecular dynamics (MD) simulations in ref 32 did not reproduce this face-selective preference in studies involving the Smoothened (SMO) receptor, a class F GPCR, and with serotonin (5-hydroxytryptamine/5-HT) receptors (5-HTR), e.g., type 1A (5-HΤ_1A_R).^33^

**Figure 1:**
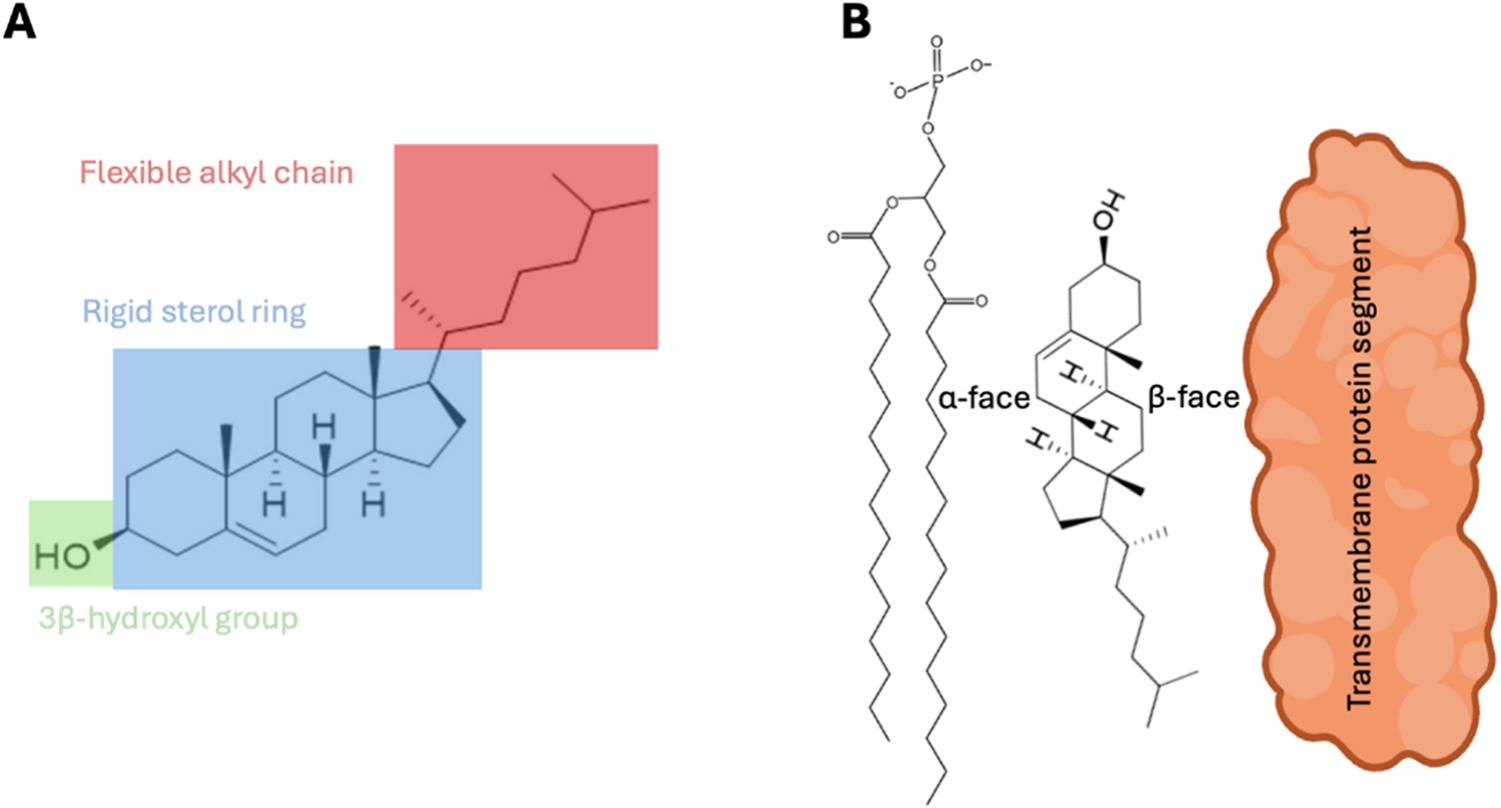
Schematic representation of key structural features of cholesterol and its interactions with membrane proteins. (A) Molecular structure of cholesterol highlighting its three distinct regions: a flexible iso-octyl side chain (red), a rigid near-planar tetracyclic sterol ring (light blue), and a polar 3β-hydroxyl group (light green). The apolar (the sterol ring and the isooctyl side chain) and polar (3β-hydroxyl group) portions provide cholesterol with an amphipathic nature that facilitates interaction with other membrane constituents (lipids and proteins). (B) Diagram of cholesterol orientation within a lipid bilayer, relative to phospholipids and membrane proteins. Cholesterol is positioned such that its apolar moiety aligns with the phospholipid fatty acyl chains and/or the TM segments of membrane proteins, while the polar hydroxyl group interacts with the carbonyl groups of phospholipids or forms hydrogen bonds with charged or polar amino acid residues Cholesterol exhibits molecular asymmetry due to methyl substitutions on its β-face, giving the sterol ring plane distinct structural characteristics. The smooth α-face—composed exclusively of axial hydrogen atoms—forms favorable van der Waals interactions with the saturated fatty acyl chains of phospholipids. In contrast, the rough β-face, marked by protruding methyl groups, interacts preferentially with TM protein segments..^34^

Overall, cholesterol’s ability to fine-tune the balance between membrane order and flexibility is essential for maintaining membrane integrity and functionality, supporting vital cellular processes such as signal transduction and nutrient transport under varying environmental conditions.^23,24^ Specifically, cholesterol increases acyl chain order and hydrophobic thickness of the bilayer^35,36^ and modifies the energy of elastic deformations in the membrane, generally increasing the mechanical stiffness.^36,37^ These effects are attributed to the increased lateral area compressibility and elastic bending modulus.^36,37^ Cholesterol demonstrates a preferential association with negatively curved monolayers,^38^ diminishes water penetration into the membrane,^39^ facilitates phase separation and partitioning selectively between distinct coexisting lipid phases.^40,40,41^

#### 1.2.3 The Function of GPCRs Is Modulated by Cholesterol

Depending on the receptor, cholesterol can exhibit different modulatory effects. It may act as a positive allosteric modulator (PAM), enhancing agonist-mediated signaling, or as a negative allosteric modulator (NAM) acting in the opposite direction. Importantly, PAMs cannot activate the receptor in the absence of an orthosteric agonist. In some cases, receptor signaling remains unaffected by cholesterol.^34,42–46^ The PAM or NAM effect can be attributed to the direct association of cholesterol with specific hydrophobic receptor sites of the receptor^47,48^ (Figure 2).

**Figure 2:**
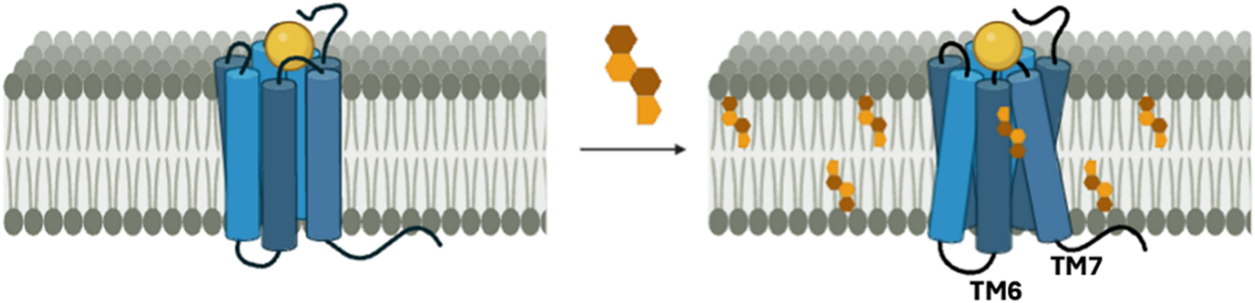
Cholesterol can modulate GPCRs’ function by direct association.^49^ Structural representation of TM helices of the GPCR depicted as rods, while the EC and IC loops, along with the C-terminus, are represented as solid lines.

These direct interactions can shift the receptor’s conformational equilibrium toward active or inactive conformations, respectively, thereby modulating signaling efficacy.^15,50,51^ Cholesterol-interaction sites in GPCRs of different classes have been discussed; see, for example, refs 15,43–45,51–53 . In contrast to the term “binding site” typically used for high-affinity ligand interactions, we prefer the more appropriate term “cholesterol-interaction site”, reflecting the generally weaker and more transient nature of cholesterol’s association with GPCRs^54–58^ (Exceptions apply when referring to standard concepts like “binding affinity” or “free energy of binding.”). Cholesterol can also affect GPCRs’ function indirectly through membrane perturbation^59^ and changes in bulk membrane bilayer physical properties, like order and fluidity. For many GPCRs, the precise nature of cholesterol’s influence remains incompletely understood.^42,60,61^

#### 1.2.4 Class A G Protein-Coupled Receptors

Class A GPCRs represent targets for approximately 36%^62^ of commercial drugs.^63–65^ These receptors are categorized according to ligand specificity into aminergic, peptide, protein, nucleotide, lipid, steroid, melatonin, aliphatic carboxylic acid, olfactory, and sensory. Despite their structural diversity, class A GPCRs share a conserved signaling mechanism governed by a set of conformational “microswitches” spanning EC and IC domains.

In the fully activated conformation of a class A GPCR, an agonist binds to the orthosteric binding site (OBS)—an extracellular pocket formed within the 7TM bundle, primarily involving segments TM3, IL2, TM4, EL2, and TM5—while the cytoplasmic region engages with the Gα subunit of the heterotrimeric G protein. Agonist binding stabilizes a subset of conformations within the receptor’s conformational ensemble, shifting the equilibrium toward active states. These active states are defined by coordinated conformational states in a conserved network of “microswitch” motifs in class A GPCRs. This network consists of key conserved motifs that include residues located in both EC and IC regions of the receptor. These motifs include: D^3.49^R^3.50^Y^3.51^ (E/DRY), P^5.50^I^3.40^F^6.44^ (PIF), C^6.47^W^6.48^xP^6.50^ (CWxP), N^7.49^P^7.50^xxY^7.53^ (NPxxY), along with the conserved Y^5.58^ residue in TM5.^2,66–69^ For the stabilization of the fully activated state of a class A GPCR, the most characteristic conformational changes are observed in the N^7.49^P^7.50^xxY^7.53^ motif^70^ and the activation state is heavily reliant on Y^7.53^ conformation.^70^ The downstream signaling of many receptors is decreased or eliminated by the mutation of Y^7.53^.^70^

In detail, successful coupling of a class A GPCR to a G protein requires that the microswitch motif network must adopt the active conformation. Specifically, compared to the inactive conformation, this needs the inward movement of residues I^3.40^ from the PIF motif and R^3.50^ from the E/DRY motif within TM3 pushes F^6.44^ (PIF motif) and L^6.34^ outward, resulting in the outward displacement of the cytoplasmic portion of TM6. Simultaneously, the intracellular part of TM5 swings outward, while its central and EC segments shift inward by ∼2.5 Å. A key step in activation is the downward movement of Y^5.58^ in TM5, enabling a water-mediated hydrogen bond with Y^7.53^ of the NPxxY motif^70^—forming the so-called “YY-lock.” Cοmpared to the inactive conformation, this interaction stabilizes the outward swing of TM6 by ∼7–14 Å, creating a cytoplasmic cavity for Gα protein binding, as demonstrated in crystallographic, biophysical, and biochemical studies.^3,62^ Additionally, the conformational shift of W^6.48^ (“toggle switch”) further facilitates this process. In the agonist-bound receptor state TM5, TM6 α-helices remain highly dynamic to mediate this pronounced conformational change, observed in numerous class A GPCRs, that enables coupling with a G protein. Alternatively, GRKs can phosphorylate IL3 and the C-terminus, and β-arrs interact with phosphorylated IL3 and the C-terminus.^71^ Once recruited, activated G proteins or β-arrs can further engage downstream effectors, initiating a broad range of physiological signaling responses.^2,72–75^

It has been reported through structural^76,77^ and biochemical assays that certain class A GPCRs can assemble into homo- and heterodimers, or even larger oligomeric complexes, which play a role in allosteric regulation of their function, e.g., in ligand binding or receptor signaling and trafficking.^8,78,79^ The dimerization of class A GPCRs has been suggested to be more likely.^78^ While it is known that monomeric class A GPCRs activate multiple G proteins^80^ and β-arrs,^81–86^ class A GPCR dimerization or oligomerization can alter the selectivity for G protein coupling, as was shown with the chemokine receptors, C–C chemokine receptor type 2 (CCR2), and C-X-C chemokine receptor type 4 (CXCR4),^87^ and can cause an increase^88^ or a decrease^89^ in G protein activation compared to monomeric GPCR, as revealed for the CXCR4 or the neurotensin receptor type 1 (NTS1R), and can impact intracellular trafficking. Nevertheless, it is still unknown how GPCR dimerization or oligomerization affects β-arr engagement, as has been explored, for example, for the μ-opioid receptors (μORs)^90^ and the chemokine receptors CXC4R and CCR2.^87^ Both the function and the dimerization/oligomerization behavior of class A GPCRs are influenced by the lipid composition of the membrane.^1,15,42,50,59,91–95^ In particular, cholesterol-rich lipid rafts serve as essential signaling hubs for many class A GPCRs. Receptors such as β-adrenergic receptors (βARs), dopamine receptors (DRs), and muscarinic cholinergic receptors (mAChRs) are known to localize within these cholesterol-rich lipid rafts.^96^ As noted earlier, caveolin-1 contributes to lipid raft organization and GPCR localization, particularly in receptors like D1R, M2R, and β_2_AR.

### 1.3 Examples of General Findings on the Interaction between Cholesterol and Class A GPCRs

#### 1.3.1 Biochemical Assays

Cholesterol has been found to affect the stability of^44,97–99^ agonists’ affinity,^99^ activation and signaling,^100–102^ and oligomerization^8,103–106^ of various class A GPCRs. For example, cholesterol acts as a PAM of the CXCR4, as demonstrated by the intracellular Ca^2+^ responses assay,^107^ and of the δ-opioid receptor (δOR) as shown by a radiolabeled GTP-γ-S binding assay (a nonhydrolyzable analog of GTP), as well as a coimmunoprecipitation assay involving Gαi proteins and caveolin-1.^108^ In contrast, cholesterol has been reported to function as an NAM of the κ-opioid receptor (κOR) based on a cAMP production assay^109^ or a radiolabeled binding assay that measures agonist-induced binding of radiolabeled GTP-γ-S,^110^ and of the M2 mAChR based on a cAMP production assay.^111^

Competition radioligand binding studies have been applied to explore the effect of cholesterol on GPCR-ligand binding affinity. Depending on the receptor, cholesterol can act both as a PAM and as an NAM, as shown with the CCR3,^112^ the δOR^108^ or the κOR^110^ and the β_2_AR,^113^ respectively. Additionally, while cholesterol acts as a PAM of the cholecystokinin receptor type 1 (CCK1R),^114–116^ no modulatory effect has been observed for the cholecystokinin receptor type 2 (CCK2R).^114,115,117^ Conversely, the cholesterol’s association strength in GPCR structures can change by ligand binding compared to the apo-receptor state or can change according to the type of ligand, being different between agonists and antagonists.^43,44^ For instance, a study employing radioligand-binding assays on A_2A_ adenosine receptor (A_2A_R) showed that the affinity of cholesterol was significantly reduced upon binding of an antagonist to the receptor.^118^ Biochemical assays showed that cholesterol also affects the oligomerization of class A GPCRs.^8,106^ For example, cholesterol stabilizes μOR homodimers by interacting with a palmitoylated cysteine residue in the receptor’s cytoplasmic region, near the G protein coupling site. Cholesterol depletion impairs signaling^102^ and removal of the palmitoylation site reduces cholesterol affinity, thereby diminishing receptor function.

#### 1.3.2 Biophysical Methods

##### 1.3.2.1 Experimental Structures

Cholesterol can interact directly with GPCRs mainly through hydrophobic contacts. Structural data from X-ray or cryogenic electron microscopy (cryo-EM) structures reveal that these interactions frequently involve multiple residues located either at the interfaces between TM1–7, H8 helices, or within a TM helix. It has been reported^45^ in a sample of 560 experimental X-ray and cryo-EM structures of GPCRs, the presence of cholesterol was confirmed in 240. However, accounting for redundancy (i.e., the same receptor solved under different conditions or using different methods), only around 68 of these represent structurally distinct GPCRs, primarily of human origin. Most notably, the A_2A_R and β_2_AR dominate this subset. Table 1 presents Protein Data Bank accession (identification) codes (PDB IDs) for representative class A GPCRs in which cholesterol-interaction sites have been resolved, primarily from X-ray structures.

**Table 1:** Cholesterol-Interaction Sites in Experimental Structures of Some Class A GPCRs

**IC sites**			
**TM1/H8**	**TM1/TM2, TM2**	**TM4**	**TM2–TM4** or **TM4**
β_2_ AR-agonist	5-HT_2A_ R-agonist	**TM5/TM6**	β_2_ AR-antagonist
5D6L,5JQH	7WC4,7VOD	CX3CR1-agonist-Gi	2Y00,3D4S,3NY8,3NYA,3PDS, 5D5A
β_2_ AR-inverse-agonist	β_2_ AR-agonist	7XBX	β_2_ AR-agonist or -inverse agonist
2RH1	5D6L,5JQH	apo-CX3CR1-Gi	5D6L,6PS0,6PRZ, 2RH1,5D5B
5-HT_2B_ R-antagonist or -agonist	CCK_1_ R-agonist-Gi	7XBW	β_1_ AR-agonist or -antagonist
4IB4,4NC3, 5TVN,3D4S,5D5A	7MBY		2Y00,2Y04,3ZPQ
5-HT_2A_ R-agonist			5-HT_2A_ R-antagonist, or -agonist
7WC4,7VOD	**ΤM3/TM4/ΤM5, TM5**	**TM6/TM7, TM6**	6A93,6A94,7WC5
5-HT_2A_ R-inverse agonist	β_1_ AR-agonist	5-HT_2A_ R-antagonist	CB_1_ R-agonist or -agonist-PAM
6WH4	2Y00, 2Y04	7WC8	5XR8, 5XRA,7V3Z or 7FEE
AT_1_ R-agonist-NbAT110i1	5-HT_2A_ R-antagonist	5-HT_1A_ R-agonist-Gi	apo-CX3CR1-Gi
6DO1,6OS1	7WC8	7E2Y	7XBW
5-HT_1A_ R-agonist-Gi	P2Y1R-antagonist	apo-5-HT_1A_ R-Gi	5-HT_1A_ R-agonist-Gi
7E2Y	4XNV	7E2X	7E2Y
apo-5-HT_1A_ R-Gi	CB_1_ R-agonist-Gi	D_1_ R-agonist-PAM-Gs	apo-5-HT_1A_ R-Gi
7E2X	6N4B	7LJC, 7LJD	7E2X
	CX3CR1-agonist-Gi		5-HT_4_ R-agonist-Gq
	7XBX		7XT8
	apo-CX3CR1-Gi		5-HT_2A_ R-agonist-Gq
	7XBW		6WHA
	D_1_ R-agonist-PAM-Gs		D_1_ R-agonist-Gs or -agonist-PAM-Gs
	7LJC		7CKW,7CKX,7X2C,7CKZ,7X2F 7LJD
			CB_1_ R-agonist-Gi
			6N4B, 6PT0
			D_1_ R-agonist-PAM-Gs

**EC sites**					
**TM1, TM1/TM2**	**TM2/TM3**	**TM1/TM7, TM7**	**TM4/TM5, TM4, TM5**	**TM5/TM6, TM6**	**TM6/TM7**
5-HT_2A_ R-antagonist	A_2A_ R-antagonist	P2Y1R-antagonist	P2Y1R-antagonist-NAM	A_2A_ R-antagonist 5JTB,5K2A,5UVI 4EIY,5IU4	A_2A_ R-antagonist
7WC8	4EIY,5IU4,5JTB,5K2A,5UVI	4XNV	4XNV	5-HT4-agonist-Gs	4EIY,5IU4, 5JTB,5K2A,5UVI
D_1_ R-agonist-PAM-Gs	5-HT_4_ -agonist-Gs	5-HT_1A_ R-agonist-G_i_	OXTR-antagonist	7XT8,7XT9	μOR-antagonist
7LJD	7XT8,7XT9	7E2Y	6TPK	β_1_ AR-agonist-Nb6B9	4DKL
		apo-5-HT_1A_ R-G_i_	D_1_ R-agonist-PAM-Gs	7BTS	5-HT_2A_ R-agonist
		7E2X	7LJC, 7LJD	D_1_ R-agonist-PAM-Gs	VOD,7VOE,7WC4, 7WC5
		CB_1_ R-agonist-Gi		7LJC	5-HT_2A_ R-agonist-Gq
		6PT0			6WHA
					5-HT_4_ -agonist-Gs
					7XT8,7XT9
					5-HT_7_ R-agonist-Gs
					7XTC

The experimental structures of these class A GPCRs are in inactive conformations, as complexes with antagonist or inverse agonist, in intermediate-active, preactive conformations as complexes with only agonist, and fully activated conformations as complexes with agonist and a G protein or a G protein mimetic nanobody (Nb), e.g., Nb6B9 or Nb80 or NbAT110i1. The receptors listed in Table 1 are the β_1_AR and β_2_AR, A_2A_R, 5-HT_1A_R, 5-HTR type 2B, type 4 (5-HT_2B_R, 5-HT_4_R), μOR, angiotensin II receptor type 1 (AT_1_R), purinergic receptors type P2Y, P2Y12R and P2Y1R, cannabinoid receptors type 1 and 2 (CB_1_R and CB_2_R), D_1_R, NTS1R, CCK_1_R, and CX3CR1. These class A GPCRs have interesting functional responses based on their cholesterol-sensitive activity, relevance to human health, or significance as drug targets. More detailed analysis and discussion of cholesterol-interaction sites and the structural/functional significance for many of these class A GPCRs are provided in Section 2.

Notably, the experimental structures of βARs (particularly β_2_AR) and 5-HTRs (notably 5-HT_1_AR and 5-HT_2_AR) account for a large proportion of all GPCR structures with resolved cholesterol molecules. The number and location of cholesterol molecules vary across structures, typically ranging from one to four cholesterol molecules per receptor, located in cavities between TMs or along the TM helices on both membrane leaflets. The analysis of the structural data showed^15,45^ that there are some frequent cholesterol-interaction sites (that appear more than once), such as the IC sites between TM2/TM3/TM4 (TM2–TM4) or between TM1/H8 or between TM1/TM2, TM3/TM4/TM5, EC sites located at TM5/TM7, and TM6/TM7. Cholesterol, or its more hydrophilic analog cholesterol hemisuccinate (CHS)^119^ are used to stabilize the folding of membrane proteins in biochemical or biophysical assays and structure determination. In several experimental structures, including structures described in Table 1, cholesterols may correspond to CHS molecules. This structural evidence also suggests that cholesterol contributes to GPCR oligomerization, including μOR homodimerization,^76,105^ supporting its functional role in GPCR organization and dynamics

##### 1.3.2.2 Simulations

Docking calculations^120^ and molecular simulations—both atomistic^121,122^ and coarse-grained^49,91–93,123–125^ —have provided important insights into the dynamic interactions between cholesterol and GPCRs, such as the β_2_AR or A_2A_R,^56^ complementing experimental findings.

Noteworthy, for the exploration of interaction sites of cholesterol to GPCRs and other membrane proteins a specialized protocol named RosettaCholesterol has been developed.^126^

A robust CG MD simulation protocol for lipid–protein interactions was described in ref 15. This approach^15^ employs 4 receptors embedded in a plasma-mimetic membrane,^93^ and employs long-time-scale simulations to resolve lipid binding behavior. The analysis of the MD simulations in ref 15 utilizing PyLipid^127^ have been employed to identify GPCR-lipid interactions mostly in combination with CG MD simulations. The outer leaflet of the plasma mimetic membrane contains ganglioside (GM) lipids, while the inner leaflet features phosphatidylserine (PS), phosphatidic acid (PA), PI, and PIPs (PI-monophosphates, PI-bisphosphates, and PI-trisphosphates). Both leaflets are composed of cholesterol, phosphatidylcholine (PC), phosphatidylethanolamine (PE), and sphingomyelin (SM), while small amounts of lysophosphatidylcholine, diacylglycerol, and ceramide are also present. Originally applied to 10 membrane proteins,^128^ the protocol was expanded to 28 GPCRs^15^ of all classes. The study demonstrated that each GPCR exhibits a unique lipid-interaction fingerprint characterized by specific, long-lived cholesterol and phosphoinositide interactions that affect membrane curvature, thickness, and local organization. The study also found minimal differences in the distribution of PI-phosphate lipids and cholesterol between aminergic and nonaminergic class A GPCRs. For instance, among aminergic GPCRs, dopamine receptor type 3 (D_3_R), histamine receptor type 1 (H_1_R), and serotonin type 2B receptor (5-HT_2B_R) exhibited 3, 6, and 6 cholesterol-interaction sites, respectively. The total number of potential cholesterol-interaction sites varied widely and can be even 8 in CB_1_R and β_2_AR.

The spatial distribution of saturated and polyunsaturated lipids, relative to cholesterol density, was found to be highly nonuniform across GPCRs. An interactive web tool built on these results (https://bisejdiu.github.io/GPCR-lipid-interactions) allows exploration of 3D cholesterol/PIP density maps, membrane thickness, and curvature profiles.

Compared to X-ray data,^15,45^ (see Table 1) in simulations, the most frequent interaction sites were interfaces between two or three TMs, often involving EC and IC loops, e.g., the IC surfaces at TM2–TM4, TM1/H8, and EC/IC at TM4/TM5, TM5, TM6, TM6/TM7. For many GPCRs of class A, the simulations showed that the hydrophobic residue at TM6 position 6.46 (commonly occupied by valine, leucine, or isoleucine) preferentially interacts with cholesterol. Cholesterol interactions that primarily engage TM1 are facilitated by interactions between the “ROH” cholesterol’s bead and H8 residues. In the CG MD simulations, it was found that cholesterol’s hydroxyl group bead is well resolved from the hydrophobic skeleton and can bind charged but also uncharged residues. Aromatic side chains also participate in stabilizing cholesterol-interaction sites. Some simulations have used simplified membranes, replacing lipid tails with single carbon solvent ethane (SCSE) to reduce complexity while preserving key cholesterol interactions, as shown in β_2_AR simulations.^129^

Various methods have been applied to calculate free energies of cholesterol association to GPCRs, for example, on β_2_AR, A_2A_R, or adenosine A_1_ receptor (A_1_R), 5-HT_1A_R, 5-HT_2B_R, and μOR based on equilibrium AA MD simulations using the fractional occupancy (density) of cholesterol and an inverse Boltzmann method^57^ or analyzing the force field energy and number of cholesterol contacts,^121^ or equilibrium CG MD simulations and the densities of cholesterol,^130^ or using the potential of mean force combined with umbrella-sampling (US/PMF) calculations,^131^ or calculations using absolute binding free energy (ABFE) perturbations^54,58^ or metadynamics sampling.^54^ Another method has been developed to determine membrane protein-cholesterol binding affinities by utilizing equilibrium CG MD simulations in combination with binding saturation curves.^55^ The ranking of affinities for different sites showed good agreement between calculations and experimental values for GPCRs (e.g., the 5-HT_1A_R), which indicates that the approach can be expanded to accommodate automated and/or high-throughput analysis.^55^ These methods^54–58,121,131^ showed that cholesterol exhibits a range of ∼1–6 *kT* (1 *kT* = 2.479 kJ/mol) for the binding strength, depending on how much the cholesterol is buried in its site, or the method used. Another method for the calculation of the binding free energy of weakly bound allosteric ligands can be applied, which is based on a two-state Boltzmann model and calculation of partition coefficients.^132^

CG MD studies^8,56,133^ also revealed cholesterol’s involvement in both homodimerization and heterodimerization of chemokine receptors (CCRs),^134–136^ where it serves as an allosteric modulator of receptor function.^102,106^ Finally, a large-scale MD database (covering ∼60% of currently available GPCR structures) has been made available, enabling exploration of previously hidden cholesterol-interaction sites, lateral lipid entry paths, and allosteric hotspots across GPCR classes.^122^

##### 1.3.2.3 Overview of Biophysical Methods Used to Explore GPCR–Cholesterol Interactions

Various biochemical^137^ and biophysical^138–140^ methods are used to explore membrane protein–lipid interactions. The principal features and applications of commonly used methods are summarized in Table 2. Applications for these methods to class A GPCRs are described in Section 2. Beyond X-ray, cryo-EM and MD simulations, other biophysical techniques that are often used to investigate cholesterol-interaction contacts are solution Nuclear Magnetic Resonance (NMR) spectroscopy, Electron Paramagnetic Resonance (EPR), Single-Molecule Fluorescence (SMF) microscopy, Fluorescence Resonance Energy Transfer (FRET), and confocal Fluorescence Recovery After Photobleaching (FRAP). GPCR unfolding temperature under Lipidic Cubic Phase (LCP)^141^ conditions can be followed by measuring the melting temperature (LCP-Tm).^142^ LCP-Tm assay is combined with mutagenesis experiments or with suitable fluorescent probes attached to the GPCR to study the effect of cholesterol in receptors bearing a mutation on residues interacting with cholesterol. Additionally, Surface Plasmon Resonance (SPR) and Isothermal Titration Calorimetry (ITC), UltraViolet-visible (UV–vis) spectroscopy, and Differential Scanning Calorimetry (DSC) have been used.

**Table 2:** Summary of the Features of Biophysical Methods Used to Capture Class A GPCRs-Cholesterol Interactions

	General description	Output information	Strengths	Limitations
1. X-ray crystallography	Diffraction pattern of X-rays to infer the location and configuration of atoms in single crystals	High-resolution structure of GPCR-cholesterol complex	High-resolution method that identifies cholesterol-interaction sites	Requires crystallization of GPCRs; static structures, non-native environment
2. Cryo-EM	Electron beam captures pictures of frozen protein solutions; single particles are aligned and classified in 2D classes; reconstruction software determines 3D structure.	High-resolution structure of GPCR-cholesterol complex	Native-like environment (NLE)	Applicable generally for large complexes; may lack resolution for cholesterol; ensemble averaging
3. NMR	Local molecular changes of the nuclei of an isotopically labeled protein	GPCR-cholesterol interaction sites, dynamics	Use nanodiscs as NLE; dynamic data	Protein size limitation: not applicable for full-length-GPCR; complex spectra
4. EPR	Changes in the EPR spectrum of spin-labeled cholesterol analog by a GPCR	GPCR-cholesterol proximity	Use nanodiscs as NLE	Cannot detect direct interactions
5. SMF	Describes how a single GPCR folds or changes shape over time at the SM level on a cell membrane	GPCR-cholesterol interactions at specific sites, dynamics	Native-like environment; real-time conformational changes	Low resolution; possible mutation of residues for tagging reporter groups can perturb the GPCR conformation
6. FRET	Describes the motion of two interacting GPCRs or/GPCR and cholesterol tagged with the appropriate donor/acceptor fluorescent groups	Distances between GPCR or GPCRs and cholesterol molecules at nm proximity	NLE; measures distances at <10 nm	Low resolution; mutation of residues for tagging with reporter groups can perturb the GPCR conformation
7. FRAP	A laser beam photobleaches the fluorescence of a tagged GPCR in a specific region of the membrane, creating a dark spot	Proportion (mobile fraction) and rate (diffusion) of GPCRs to diffuse back into the bleached area according to membrane organization or contacts with cholesterol	Live-cell imaging; quantitative measurements; does not require proximity between molecules for signal	Follows movement over μm-scale but cannot resolve nanodomains like lipid rafts or detect direct GPCR-cholesterol contacts; tagging can perturb GPCR conformation; immobile fraction can be due to aggregation
8.Chemical cross-linking/MS	Reactive cholesterol analogs connected through photoactivation or CuAAC click chemistry, following MS	GPCR-cholesterol interaction sites	Accurate positions of cholesterol	Mutation of residues for tagging reporter groups can perturb the GPCR conformation
9. SPR and ITC	Describes the interaction strength between a GPCR and a ligand	Thermodynamics of binding	Measurement of the binding strength; use of liposomes	Might not detect the weak interactions, the GPCR-cholesterol interaction
10. LCP-Tm	Unfolding temperature under LCP conditions, with the use of fluorescent dyes to detect exposed hydrophobic regions as the protein unfolds with increasing temperature	The stability of GPCR is affected by cholesterol	A simple way to check if cholesterol is stabilizing/destabilizing a receptor can be combined with mutagenesis experiments	A shift in Tm could be due to global conformational changes, not necessarily to a specific cholesterol-binding event; dyes used to monitor protein unfolding can interfere
11. MD simulations	Newtonian motion and dynamics	Prediction of cholesterol-interaction sites and dynamics of GPCR	Provide predictions for combining experimental findings; describe dynamic motion	*It* depends on a force field, starting structure, and a membrane model; it needs experimental validation; computationally expensive for long time scales or large systems.

Photosensitive chemical cross-linking and “click” assays^143^ are very promising for identifying cholesterol-interaction sites. The enrichment and visualization of cross-linked receptor–ligand complexes is followed by Mass Spectrometry (MS) analysis that identifies the specific amino acid residues within the GPCR that interact with cholesterol. This provides detailed mapping of cholesterol-interaction sites at the molecular level. Thus, photoactivatable cholesterol analogs, such as diazirine-modified cholesterol analogs (e.g., KK174 and LKM38 ^144^) can mimic natural cholesterol interaction with membrane proteins while including the photoreactive diazirine group. Upon UV exposure, these analogs generate reactive carbenes that are linked covalently to nearby amino acid residues within the GPCR structure.^144^ Alternatively, incorporated alkyne or azide functional groups in cholesterol analogs enable “click chemistry” through copper-catalyzed azide–alkyne cycloaddition (CuAAC) with reporter tags in the GPCR, such as fluorescent dyes or biotin.^145^ There are only a few examples of solid-state NMR (ssNMR) spectroscopy applied to examine the effect of cholesterol in class A GPCRs, e.g., in CCR3.^146^ The ability of native mass spectrometry (nMS) to detect weakly associated lipid species like cholesterol has been questioned.

#### 1.3.3 GPCR–Cholesterol-Interaction Motifs

##### 1.3.3.1 Cholesterol Consensus Motif

The Cholesterol Consensus Motif (**CCM**) is a structural motif involving two adjacent TM helices and consists of four amino acid residues; three residues are included in the R **/** K-(X)_1–7_-**I**/**V**/**L**-(X)_1–3_-**W**/**Y** or its inverse (i.e., **W/Y**-(X)_1–3_-**I/V/L**-(X)_1–7_-**R/K**) (from inner to outer leaflet direction) and a fourth non polar amino acid residue **Y** or **F** or **W** in a nearby TM helix. This motif was identified initially in the protein hemagglutinin (HA) of the influenza virus.^147^ In GPCRs the combination of TM4 and TM2 forms a strict **CCM** motif often observed in the IC leaflet, [4.39–4.43­(R,K)]-[4.46­(**I**, **V**, **L**)]-[4.50­(**W**, **Y**)]/[2.41­(**F**, **Y**, **W**)], and has been observed for the first time in β_2_AR.^113^ The fourth amino acid residue can lie on TM5 or in its IC juxta membrane region (e.g., in IL3) in the strict **CCM**. A putative **CCM** can also be formed when the number of residues between **Y** and **I** or between **R** and **Y** varies from the strict **CCM**, as has been observed^148^ in **CCM**s from other mammalian GPCRs, for example, in 5-HT_1A_R, the **CCM** YGR **I** F­(R/K)­AA **R** F **R** and **Y** IPL­(**I**/**L**) **L** MLVLYG **R** is formed.

##### 1.3.3.2 Cholesterol Recognition Amino Acid Consensus Motif

Contiguous residue sequences located within single TM helices of membrane proteins, including GPCRs, have been identified as the Cholesterol Recognition Amino Acids Consensus (**CRAC**) motifs. These motifs include the -**L/V**–(X)_1–5_–**Y/F**–(X)_1–5_–**R/K**- sequence and are thought to play a role in stabilizing membrane proteins and GPCRs within the membrane.^44,53,149–152^ The **CRAC** motif was first experimentally identified in the peripheral-type benzodiazepine receptor (PBR),^153^ a five-TM helices domain mitochondrial translocator protein. Although PBR is not a GPCR, it is involved in the transport of cholesterol from the outer to the inner mitochondrial membrane. Additionally, cholesterol has been shown to bind to the “**CARC**” motif, which follows the reverse to **CRAC**, -**R/K**–(X)_1–5_–**Y/F**–(X)_1–5_–**L/V**- sequence as observed in the nicotinic acetylcholine receptor (nAChR).^154–156^

##### 1.3.3.3 CCM and CARC Motifs in Class A GPCRs

Although **class A GPCRs** share a conserved seven-helical TM core, their cholesterol-binding patterns vary widely. Beyond the general **CCM** and **CARC** motifs, other unique motifs have also been recognized as general cholesterol-interaction motifs in membrane proteins.^157^ Recall that, in contrast to the **CRAC/CARC** domains, **CCM** is composed of residues arranged spatially as opposed to in a linear fashion; **CCM** involves a motif in which cholesterol lies between two spatially proximal TM α-helices, including 4 amino acid residues, with which it interacts, often found in the cytoplasmic leaflet^158^ of the cell membrane. It has also been noted^148^ that in a conformation where TM2 is close to TM4, a putative **CCM** interacting with cholesterol can be formed between TM4 and TM5, since a nonpolar aromatic amino acid can become accessible, e.g., tyrosine from TM5, or phenylalanine from IL3 fragment replacing the fourth residue in TM2.

Sequence alignments indicate^51,53,159^ that the IC TM2–TM4 **CCM**, observed first in β_2_AR, extends far beyond β_2_AR and encompasses a substantial proportion of all classes of human class A GPCRs, as much as 44%,^113^ it is found, for example, in 5-HT_1A_R, A_2A_R, OXTR, D_1_R, NTS1R, M2R, sphingosine 1-phosphate receptor (S1PR), α_1_AR adrenergic receptor (α_1_AR). The bioinformatics analysis of class A GPCRs^51,53,159^ revealed the presence of **CRAC** motifs frequently in class A GPCRs, with the number of **CRAC** motifs that can vary. However, it was shown that in TM7, in which the highly conserved N^7.49^P^7.50^xxY^7.53^ (NPxxY) motif^70^ (Ballesteros-Weinstein numbering^160^ in superscript) exists, a **CRAC** motif is colocalized with the N^7.49^P^7.50^xxY^7.53^ motif^70^ in class A GPCR. This IC TM7 **CRAC** sequence was identified with sequence alignment in 38% of 285 class A GPCRs.^51,53^

While **CCM** motifs and especially the IC TM2–TM4 **CCM** have been identified in experimental structures, there are no experimental structures of GPCRs deposited in the Protein Data Bank (PDB) yet showing clearly the presence of cholesterol in a **CRAC** motif. Examples of **CCM** and **CRAC** motifs, but also of distinct motifs for a selected set of class A GPCRs, will be discussed in Section 2.

#### 1.3.4 Membrane Properties Modulation

Among lipids, cholesterol likely modulates more effectively the physicochemical properties of biological membranes (see Section 1.2.2). Cholesterol facilitates phase separation and partitioning selectively between distinct coexisting lipid phases.^40,40,41^ Thus, membrane rafts and caveolae, which are cholesterol-rich cellular domains, concentrate GPCRs.^27^ By affecting the membrane properties, cholesterol can affect the conformation of a GPCR, causing up- or downregulation of its signaling depending on the receptor.^50^ Additionally, changes in these physicochemical properties possess the ability to modify the ligand-sequestration capacity of cholesterol near the bilayer surface.^161^ These solvent-like effects from the membrane can influence the function of GPCRs,^50^ as was shown for the RhoR^162^ and suggested for the 5-HT_1A_R.^161^

## 2 Cholesterol Interaction with Selected Class A GPCRs

### 2.1 Selected Set of Class A GPCRs

#### 2.1.1 Results from Sequence Analysis

The phylogenetic analysis presented in Figure S1 supports the selection of 14 representative class A GPCRs examined in this review. Our sequence analysis, conducted on human orthologs, revealed two distinct clades: one comprising CCK_1_R, CCK_2_R, RhoR, P2Y1R, NTS1R, CXCR3, OXTR, and another including β_1_AR, β_2_AR, 5-HT_1_AR, 5-HT_2A_R, CB_1_R, CB_2_R, and A_2A_R. The analysis shows schematically that these receptors often have big differences in sequence, but nevertheless have some conserved structural and cholesterol interaction motifs. This analysis showed that the depicted IC TM2–TM4 **CCM** motif (detailed in Table S1 and illustrated in Figure S2) demonstrated a high degree of conservation across the analyzed receptors. Interestingly, all these class A GPCRs demonstrated the same **CCM** motif (TM2/TM4 or TM4) colocalized with the highly conserved N^7.49^P^7.50^xxY^7.53^ motif^70^ in class A GPCRs.^51,53^ Notably, receptors in the first clade exhibit lower conservation of **CRAC** motifs, while 5-HT_1A_R, 5-HT_2A_R, CB_1_R, CB_2_R, A_2A_R, and β_2_AR showed more similar cholesterol-interacting features, with higher conservation of the **CRAC** motif (TM7). These shared structural features may represent common cholesterol-interaction elements, though further experimental validation is required.

Despite this conservation, the individual receptors exhibit additional cholesterol-interaction motifs and substantial diversity in their modes of cholesterol interaction. For example, 5-HT_1A_R has **CRAC** motifs at EC TM2, IC TM5, IC TM7, RhoR at EC TM1, EC TM3, IC TM7, and β_2_AR at IC TM5 and IC TM7.^51,53,159^ Additionally, 5-HT_1A_R and D1R have the same **CRAC** motif in TM5 while β_1_AR, P2Y1R, OXTR did not exhibit such a **CRAC** motif in TM5 or TM7. CB_1_R has one **CRAC** motif at IC TM7, CCK1R has two **CRAC** motifs at EC TM1 IC TM7/H8 and IC TM3 **CRAC**, NTS1R has **CRAC** motifs at EC TM1 and IC TM6.^51,53^ The number of amino acid residues in each motif can also vary; 5-HT_1A_R has 12 residues in all **CRAC** motifs while the number of residues in **CRAC** motifs of RhoR range from 8 to 11 and in β_2_AR ranges from 9 and 5 residues.^51,53^

Comparisons among subfamily members (e.g., A_2A_R vs A_2B_R, A_1_R, A_3_R; or β_1_AR vs β_2_AR; or 5-HT_1A_R vs 5-HT_2A_R/5-HT_2B_R) revealed that even structurally similar receptors do not always share identical cholesterol-interaction profiles. For instance, β_1_AR and A_1_R lack the TM7 CRAC motif observed in their close homologues β_2_AR and A_2A_R, respectively (see Table S1 and Figure S2), despite overall high sequence similarity.

### 2.2 RhoR

#### 2.2.1 Biochemical Assays–UV–Vis–NMR Spectroscopy

Experimental studies showed that cholesterol depletion in the native rod outer segment (ROS) membranes (retina, in vivo) affects RhoR activation, indicating that cholesterol is crucial for the receptor’s function.^163^ In this context, it was observed that elevated cholesterol concentrations stabilize the inactive form of RhoR, metarhodopsin I **(MI**), thereby inhibiting the transition to the intermediate-active state, metarhodopsin II (**MII**).^12,13,164,165^

Crystal structures have been solved for bovine rhodopsin in various conformational states: the dark state (dark opsin), the inactive **MI** state, the intermediate-active **MII** state, and opsin (PDB IDs: 1U19,^166^ 3PXO,^167^ 3CAP,^168^ respectively). Opsin exists in an equilibrium between inactive-like and active-like receptor conformations and has been crystallized under low-pH conditions. Its root mean-square deviation of Cα carbons, RMSD (Cα), is 0.51 relative to **MII** and 2.8 Å relative to the dark-state structure. However, at pH ∼ 6.1, its Fourier Transform Infrared Spectrum (FTIR) closely resembles that of the dark state, and the apo protein demonstrates limited ability to activate transducin. Therefore, dark opsin can be considered a model for the hypothesized inactive-like opsin (**MI**) structure.

Several studies have investigated the effect of lipid bilayer composition—particularly lipid order and bilayer packing—on the equilibrium between **MI** and **MII**.^9–14^ Results from these studies revealed that the interactions between rhodopsin and lipid species in the first layer of lipids surrounding the protein, and the membrane elastic stress, play a critical role in regulating the **[MII]**/**[MI]** ratio. In ref 169 the regulation of RhoR by some dietary lipids was also investigated. RhoR was reconstituted in liposomes, and DSC revealed that cholesterol enhanced the thermal stability of the proteoliposomes and shifted the **MI**–**MII** equilibrium toward the inactive MI state, as indicated by absorption at 500 nm.

Another study systematically varied cholesterol content (up to 30 mol %) in PC bilayers with saturated, perdeuterated sn-1 acyl chains and monounsaturated sn-2 acyl chains of different lengths (14:0–14:1 PC, 16:0–16:1 PC, 18:0–18:1 PC, and 20:0–20:1 PC), yielding bilayers with hydrophobic thicknesses from ∼21 to 38 Å.^14^ Ultraviolet–visible (UV–vis) spectroscopy was used to examine the equilibrium between the inactive (**MI**) and active (**MII**) states of RhoR, a model GPCR, in these bilayers. Additionally, ^2^H ssNMR was performed at 37 °C on oriented phospholipid bilayers to analyze the ordering of hydrocarbon chains and measure bilayer hydrophobic thickness. The results showed that cholesterol-induced changes in bilayer thickness within the range of the protein’s hydrophobic thickness significantly influenced **MII** formation, aligning with predictions from continuum elastic modeling. Specifically, the results showed that cholesterol shifts the equilibrium toward the **MII** state in bilayers thinner than 27 Å, which is the average length of hydrophobic alkyl chains. In contrast, in thicker bilayers, cholesterol favored the **MI** state and promoted RhoR oligomerization. Atomistic MD simulations further confirmed the proximity of cholesterol molecules to the receptor. These findings indicate that in membranes with physiological thickness—where the bilayer and protein hydrophobic thicknesses are well matched—cholesterol-induced changes in local elastic deformations around the protein play a dominant energetic role in modulating the **MI–MII** equilibrium. Thus, cholesterol exerts regulatory effects on RhoR activity through both direct interactions and modulation of the membrane’s mechanical environment.

#### 2.2.2 Results from Bioinformatics Analysis–FRET–Cryo-EM–MD Simulations

Sequence alignment studies^53^ have identified three **CRAC** motifs in RhoR at EC TM1, IC TM3, and IC TM7, in addition to the IC TM2–TM4 **CCM** (see Table S1). The fluorescent sterol cholestatrienol was used as a probe to investigate interactions between cholesterol and RhoR in bovine rod outer segment disk membranes. Cryo-EM studies were also conducted to assess the presence of cholesterol-binding sites.^165^ FRET quenching from tryptophan residues of RhoR to cholestatrienol was observed, indicating spatial proximity between the sterol and these residues. The extent of quenching was directly correlated with the concentration of cholestatrienol in the membranes and was inhibited by the addition of cholesterol, with the degree of inhibition proportional to cholesterol content, suggesting direct, competitive binding of both sterols to RhoR.

In a low-resolution (5.5 Å) cryo-EM structure of RhoR,^170^ in used to investigate dimeric arrangements, a diffuse density was detected between antiparallel dimers of RhoR, tentatively assigned as a single cholesterol molecule.^170^ This putative site was located at the EC TM6/TM7 of one RhoR molecule and the IC TM4 of a neighboring RhoR molecule. Τhese sites have been seen to interact with cholesterol also in other class A GPCRs (see Table 1). However, the low resolution of the cryo-EM structure and the nonphysiologically relevant antiparallel orientation make the presence of cholesterol quite ambiguous.

##### 2.2.2.1 AA MD Simulations

Multiple AA MD simulations were performed^171,172^ starting from the inactive RhoR (PDB ID 1U19 ^166^) embedded in a membrane composed of 1-stearoyl-2-docosahexaenoyl-*sn*-glycero-3-phosphocholine (SDPC), 1-stearoyl-2-docosahexaenoyl-*sn*-glycero-3-phosphoethanolamine (SDPE), cholesterol, and docosahexaenoic acid (DHA). SDPE phospholipids contain a PE headgroup, an 18-carbon saturated stearoyl (STEA) chain, and an ω-3 22-carbon DHA chain with six double bonds. ROS membranes are highly enriched in DHA (∼35–60% of all phospholipid acyl tails), whereas PE constitutes ∼40% of all headgroups. Hence, SDPE is a simplified approximation of rhodopsin’s lipid environment. The AA MD simulations results have argued for a nonspecific influence of cholesterol on RhoR function through increasing the membrane’s packing. Τhe simulations in refs 171,172 revealed specific interactions between cholesterol and RhoR and showed that interactions of RhoR with DHA depend on the receptor’s conformational state. The highest cholesterol density was calculated in two areas of the RhoR: (a) The EC TM1/TM7, which matches approximately the high-density sterol region observed in the cryo-EM structure.^170^ (b) The IC TM2–TM4 region, aligned with a **CCM** observed in many of the X-ray structures of class A GPCRs (Table 1). The presence of cholesterol close to EC V^1.58^, W^2.63^, P^7.38^ was found to cause significant local perturbation in the TM domains. These changes included kinks in TM1, TM2, and TM7 that cause relative angular motion between TM7, H8, and movement of TM1 relative to TM7, which shortened the distances between TM1 and TM7, and between TM7 and H8. The results suggest that DHA may bind more tightly and specifically to RhoR than cholesterol, with a conformation-dependent binding mode.^171,172^

##### 2.2.2.2 CG MD Simulations

Further insights were gained from 30 μs-CG MD simulations, which showed that RhoR-cholesterol interactions differ significantly depending on the conformational state of the receptor. In inactive RhoR, three cholesterol-binding sites were found on the extracellular side. These sites were largely absent in the active state, with the most persistent site—at EC TM1/TM7—completely disappearing. The key determinant of this change was identified as F293^7.39^. In the inactive conformation, the side chain of F293^7.39^ faces inward, allowing cholesterol to interact with the EC TM1/TM7 region. In the active state, F293^7.39^ rotates outward, preventing this interaction. Conversely, the active conformation revealed a novel cholesterol-binding site on the IC region of TM7, absent in the inactive state.

### 2.3 β_2_ and β_1_ Adrenergic Receptors

#### 2.3.1 Results from Bioinformatics Analysis

The lipid–interaction profile of adrenergic receptors is of particular significance, as they are among the most extensively studied GPCRs. Bioinformatics analysis in refs 51,53,159 have identified in the β_2_AR one IC TM2–TM4 **CCM** and two **CRAC** motifs located at the intracellular TM5 and TM7 regions (see also Table S1); in β_1_AR only the IC TM2–TM4 **CCM** is present (Table S1). These cholesterol-interaction motifs have been implicated in modulating βAR function and organization.

#### 2.3.2 β_2_AR: Biochemical Assays–SMF Microscopy

Numerous studies have explored the impact of cholesterol on β_2_AR signaling and ligand binding.^108,109^ Cholesterol depletion via methyl-β-cyclodextrin (MβCD) in cardiac myocytes was shown to enhance nonraft-associated in vitro signaling of adrenergic receptors type α and β (αARs and βARs) by releasing Gαs and AC proteins from lipid raft sequestration.^173,174^ However, this effect may be attributed more to the disruption of lipid raft associations rather than a direct interaction between β_2_AR and cholesterol, with the latter acting as a NAM of β_2_AR.^174^ Similarly, increasing membrane cholesterol levels has been associated with inhibition of β_2_AR-mediated signaling.^174^ Cholesterol depletion also leads to a more diffuse spatial distribution of β_2_AR and enhanced agonist-induced signaling, while cholesterol repletion or loading reverses this effect.^175,176^

It has been established that β_2_AR is preferentially located in cholesterol-rich caveolae^177^ in cardiomyocyte cultures. This localization is mediated in part by a C-terminal PDZ-binding motif in β_2_AR, which facilitates its targeting to caveolae.^177,178^ Cholesterol-interaction motifs may further support this compartmentalization.^178^ Caveolae rich in cholesterol content function as a negative regulator of β_2_AR activation-mediated accumulation of cAMP, while the receptor partitions out of the caveolae upon stimulation. However, cholesterol does not appear to significantly influence β_2_AR clustering, which is instead more dependent on the actin cytoskeleton, especially under reduced cholesterol conditions.^179,180^

Both cholesterol and its analog CHS have been observed to enhance the stability of β_2_AR and help the receptor’s crystallization.^104,113^ Dynamic SMF microscopy experiments investigating mechanical, kinetic, and energetic parameters of β_2_AR reconstituted in 1,2-dioleoyl-*sn*-glycero-3-phosphocholine (DOPC), both with and without the CHS, at various temperatures showed that cholesterol preserves receptor structural integrity at 25 °C and enhances its stability, particularly at physiological temperature (37 °C).^98^ CHS appeared to stabilize all receptor domains, though to varying degrees, suggesting that cholesterol constrains the receptor’s conformational flexibility in a nonlinear, temperature-dependent manner.^98^

Interestingly, β_2_AR can still be expressed in functional form in *Spodoptera frugiperda* cells, which contain significantly less cholesterol than mammalian cells.^181^ Moreover, β_2_AR has been shown to bind both agonists and antagonists when expressed in *E. coli* membranes, which lack cholesterol.^182^ Although cholesterol generally acts as a NAM of β_2_AR,^108,173,174^ radioligand binding assays revealed that cholesterol increased the affinity of the partial inverse agonist timolol but had no effect on binding of the full agonist isoproterenol.^183^

#### 2.3.3 β_2_AR: X-ray Crystallography

In the X-ray structures of β_2_AR in complex with an agonist or antagonist, in which the receptor adopts an inactive-like conformation, cholesterol molecules have been observed in the IC membrane leaflet surface of the receptor (Table 1). Representative examples of experimental structures with resolved cholesterols correspond to the inverse agonist carazolol-β_2_AR (PDB IDs 2RH1,^104^ 6PS0 ^184^) and partial inverse agonist timolol-β_2_AR (PDB ID 3D4S^113^). In structures with PDB IDs 3D4S^113^ and 2RH1 ^104^ a shallow groove that provides adequate space for the interaction of two cholesterol molecules is formed at IC TM2–TM4 **CCM** of the inactive β_2_AR (Table 1, Figure 3). In the X-ray structure PDB ID 2RH1,^104^ which is one of the earliest solved experimental structures of a GPCR with cocrystallized cholesterol, a third cholesterol cocrystallized along IC TM1. In ref 113 the first observation of a cholesterol-interaction motif (**CCM**) was reported; recall that it has been estimated that the **CCM** motif exists in 21% of human class A GPCRs.^113^ In the **CCM** of inactive β_2_AR in structures with PDB IDs 2RH1 ^104^ and 3D4S,^113^ cholesterol interacts mainly with residues W158^4.50^, I154^4.46^, R151^4.43^, Y70^2.41^. The hydrophobic I154^4.46^, which engages in hydrophobic interactions with cholesterol, is 60% conserved by homology (and 35% by identity). The aromatic residue Y70^2.41^ in TM2 forms attractive hydrophobic interactions with cholesterol and hydrogen bonds with R151^4.43^ (Figure 3). Almost universally conserved (94%) among class A GPCRs, residue W158^4.50^ appears to have a substantial interaction with the sterol ring of cholesterol. Significantly, arginine or lysine at position 4.43, which can bear a positive charge, is only moderately conserved (22%).

**Figure 3:**
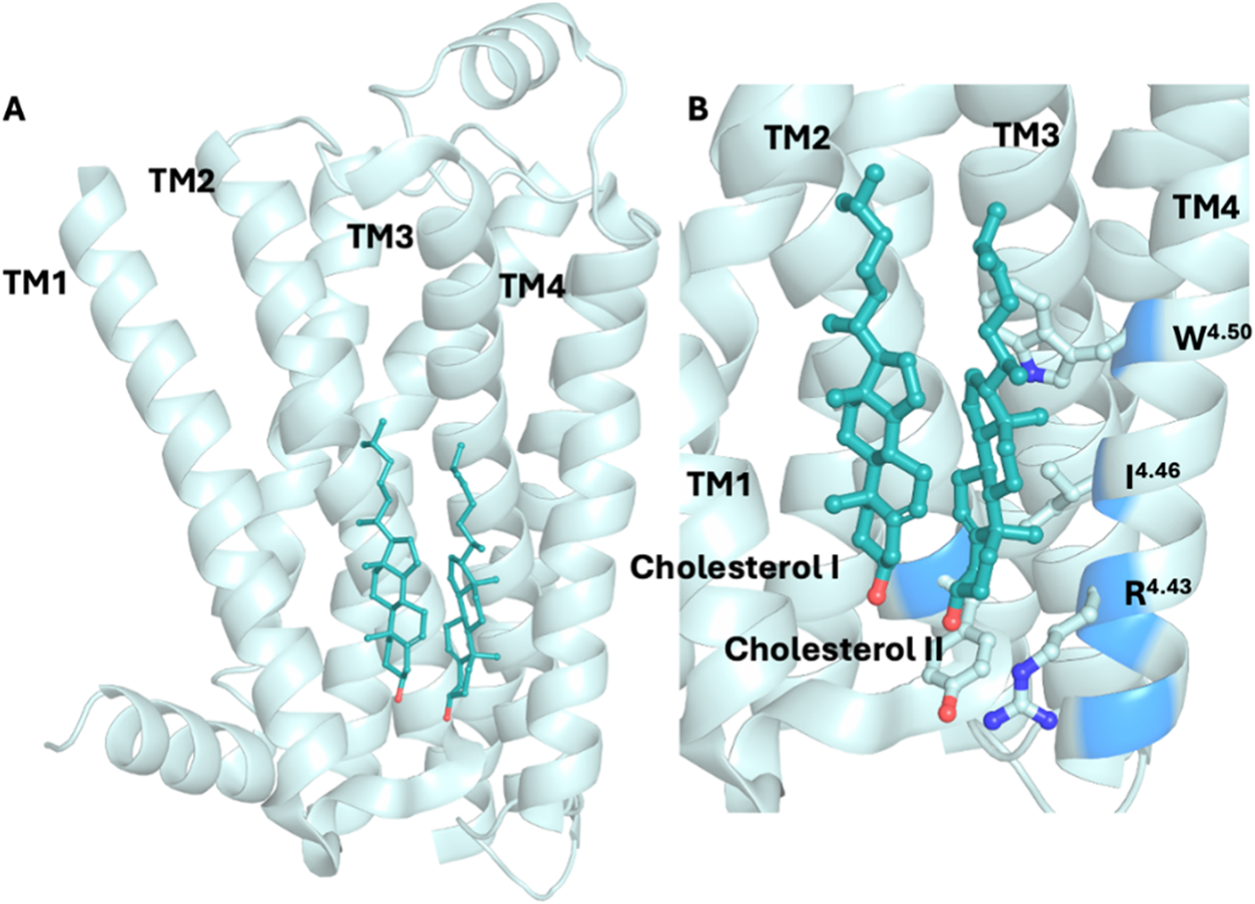
(A) Two cocrystallized cholesterol molecules are observed in the IC leaflet at the interface between TM2 and TM4 in the crystal structure of inactive β_2_AR (PDB ID 3D4S^113^), representing a **CCM**. Similarly, in another crystal structure of inactive β_2_AR (PDB ID 2RH1 ^104^), two cholesterol molecules are also present at IC TM2–TM4 **CCM**, with an additional cholesterol molecule located at the IC TM1/H8 region. The receptor is shown in ribbon representation, while cholesterol molecules are depicted as sticks. (B) The identified IC TM2–TM4 **CCM** is characterized by four key residues 4.43–4.39 of TM4 that facilitate cholesterol binding. Residues at positions 4.39–4.43 fulfill the **CCM** requirement if one or more of these positions contain an arginine or lysine residue. TM4 4.50: the most conserved and critical position in which 94% of class A GPCRs contain a tryptophan, though tyrosine can also occur; in β_2_AR, the cholesterol molecule interacts through CH-π and hydrophobic interactions with W158^4.50^, contributing the most significant interaction. TM4 4.46: typically occupied by a branched hydrophobic residue such as isoleucine, valine, or leucine; in β_2_AR, I154^4.46^ forms hydrophobic contacts with the cholesterol backbone. TM2 2.41: often contains an aromatic residue such as phenylalanine or tyrosine; in β_2_AR, Y70^2.41^ engages in hydrophobic interactions with a cholesterol molecule that is further stabilized via hydrogen bonding between its hydroxyl group and R151^4.43^.

Cholesterol interactions with β_2_AR have been documented in other crystal structures only of inactive β_2_AR. These are structures of β_2_AR in complex with antagonists (e.g., PDB ID 3NYA^185^) or with inverse agonists (e.g., PDB ID 3NY8 ^185^) or covalently bound agonist (PDB ID 3PDS^186^) in which cholesterol molecules cocrystallize at IC TM2 and IC TM2–TM4 **CCM**. In contrast, no cholesterol molecules have been resolved in crystal structures of fully activated β_2_AR bound to G proteins or G protein-mimetic nanobodies (Nb), or in agonist−β_2_AR−β-arr complexes. This difference may reflect conformational changes in TM2, TM4, and TM5 upon activation, which alter the accessibility of the **CCM**.^45^

Interpretation of cholesterol positions in X-ray structures must be made with caution due to methodological limitations. First, crystallization often employs cholesterol and CHS as stabilizing agents in LCP conditions. However, electron density maps cannot always distinguish cholesterol from CHS. Second, CHS may not accurately replicate cholesterol interactions.^119,187^ Third, in crystal structures of GPCRs, the origin of the cocrystallized cholesterol or CHS can be an artifact of the packing in the applied LCP conditions.^141^ Additionally, dimer interfaces observed in crystal structures may not reflect physiologically relevant oligomerization states and may instead be artifacts of crystallization driven by hydrophobic mismatch.^141^ Thus, in PDB ID 2RH1 appeared as a dimer^104^ the third cholesterol molecule in IC TM1/H8 might stabilize dimer formation. While β_2_AR appears dimerized in the crystallographic unit cell (due to in meso-crystallization conditions), it remains unclear whether such dimers are physiologically relevant.^188^ As discussed in Section 2.3.2, β_2_AR clustering in cells appears insensitive to cholesterol levels, and β_2_AR may not dimerize in cells.^179,180^ However, it is interesting to note that this third cholesterol site overlaps with a palmitoylation site at C341^7.68^, a post-translational modification also observed in μOR.^102^

#### 2.3.4 β_2_AR: Simulations

Computational methods, including docking calculations and both AA and CG MD simulations, have provided valuable insight into cholesterol-interaction sites in β_2_AR.

##### 2.3.4.1 Docking Predictions

Using the RosettaCholesterol docking protocol, cholesterol-binding sites were predicted at the EC TM1/TM7 interface, the intracellular IC TM1/H8 region, and the IC TM2–TM4 **CCM**. These predictions are in strong agreement with experimental observations from X-ray crystallography (see Table 1).^126^

##### 2.3.4.2 AA MD Simulations

Long-time-scale AA MD simulations performed in 1-palmitoyl-2-oleoyl-*sn*-glycero-3-phosphocholine (POPC)/cholesterol membranes used crystal structures of inactive β_2_AR (PDB IDs 2RH1,^104^ 3D4S^113^) as starting points (without ligands), as well as of inactive β_1_AR (PDB ID 2VT4 ^189^) for comparative purposes.^149^ These simulations revealed up to eight putative cholesterol-interaction sites on β_2_AR, including: IC TM2–TM4 **CCM**, IC TM5/TM6, EC TM5/TM6 and TM6/TM7, IC TM1/H8 (also observed in the dimer interface in 2RH1 ^104^). Most of these correspond to recurrent cholesterol-binding sites identified in crystal structures (Table 1). Simulations also showed cholesterol occupying the IC TM1/H8 site as dimers, matching crystallographic observations.

Further AA MD simulations used both inactive (PDB IDs 2RH1,^104^ 3D4S^113^) and active (PDB ID 3SN6 ^190^) conformations.^121^ Cholesterol is consistently located at IC TM2–TM4 **CCM**, IC TM5/TM6, and EC TM5–TM7 in the inactive form. However, cholesterol occupancy was lower at IC TM1/H8, suggesting this site may be involved in crystallization artifacts or context-dependent interactions. Importantly, interactions at IC TM5–TM7 hindered helical mobility, reducing receptor flexibility and potentially affecting transition to the active state. In the active β_2_AR, cholesterol still is located at IC TM5–TM6 (TM5/IL2/TM6), suggesting state-dependent interactions. Notably, cholesterol concentrations ≥ 10 mol % significantly reduced β_2_AR flexibility, attributed to direct receptor–cholesterol interactions rather than indirect effects on membrane properties. Additionally, certain cholesterol-binding sites showed affinity-dependent behavior, as their occupancy was sensitive to cholesterol concentration.

##### 2.3.4.3 CG MD Simulations

CG MD simulations, starting from the inactive-state X-ray structure of β_2_AR (PDB ID 2RH1 ^104^), which features two cocrystallized cholesterol molecules at IC TM1/H8 and IC TM2–TM4 **CCM**, successfully recovered these key cholesterol-interaction sites.^149^ The simulations also explored cholesterol’s impact on β_2_AR dimerization. In cholesterol-depleted bilayers, the receptor formed dimers via TM4/TM5 interfaces, like those observed in previous 8 μs-AA MD simulations.^191^ As cholesterol content increased (up to 50%), the TM4/TM5 interface became progressively disrupted due to cholesterol occupancy at TM4. This favored the emergence of a new dimer interface composed of TM1/TM2 helices. These findings highlight a direct competition between cholesterol–protein and protein–protein interactions, consistent with other membrane protein systems.^192^ In an additional 50 μs-CG MD simulation,^56^ the structure of β_2_AR bound to the partial inverse agonist timolol (PDB ID 3D4S^113^) was used as a starting point. In the ligand-free system, cholesterol was predominantly found at EC TM1/TM7 and IC TM3/TM4/TM5. With timolol bound, cholesterol still interacted within the IC region, but was engaged in different residues, suggesting ligand-dependent modulation of cholesterol-binding profiles.

Furthermore, CG MD simulations of β_2_AR embedded in a plasma mimetic membrane environment^15^ reproduced cholesterol-interaction sites at IC TM2–TM4 **CCM**, IC TM4/TM5, EC TM1/TM7, and EC TM5–TM7 sites also observed in crystal structures of class A GPCRs and found to be major interaction regions in the A_2A_R receptor, indicating shared cholesterol-binding features among aminergic GPCRs.

Overall, the IC TM2–TM4 **CCM**, identified in crystal structures of inactive β_2_AR, is consistently reproduced by docking, AA, and CG MD simulations,^121,126,149,193^ supporting its role as a genuine cholesterol-interaction site. Furthermore, simulations have uncovered cholesterol-binding regions at TM5–TM7, which are not observed in crystallographic data but may play a regulatory role in conformational transitions.

Finally, long-time scale CG MD simulations starting from the carazolol−β_2_AR complex (PDB ID 2RH1 ^104^) in POPC/cholesterol membranes (20 or 40%) revealed cholesterol binding to L324^7.51^, Y326^7.53^, and R328^7.55^, forming part of the highly conserved IC TM7 **CRAC** motif.^183^

#### 2.3.5 β_2_AR: LCP-Tm Assay–NMR Spectroscopy–Biochemical Assays

LCP-Tm and solution NMR experiments provided information on the binding strength of cholesterols to β_2_AR. In ref 194 analysis of the β_2_AR unfolding temperature with the LCP-Tm assay estimated the number of high-affinity cholesterol interaction sites to range from three to five. These sites were found to exhibit subnanomolar dissociation constants, consistent with long-time AA MD simulations reported in ref 193, which identified seven putative cholesterol interaction sites as discussed in Section 2.3.4. The STD NMR investigation of LCP samples of β_2_AR in ref 194 also revealed a second class of low-affinity cholesterol interaction-sites, with a lower-limit dissociation constant of approximately 100 mM. One possible explanation for this observation is that the microenvironment generated by tightly bound (nonannular) lipids may promote the association of lower-affinity (annular) cholesterol molecules.^195^ Such an environment may influence the recruitment of β_2_AR into cholesterol-rich domains^177^ as well as its oligomerization state.^149^

Despite the prediction of an IC TM7 **CRAC** motif by the CG MD simulations in ref 183, biochemical data from the LCP-Tm assay, combined with site-directed mutagenesis did not support this prediction. Incorporation of 10% cholesterol into the host lipid system increased the Tm, as assessed by the LCP-Tm assay. Two residues, F321^7.48^ and R328^7.55^, were selected for the mutagenesis studies. F321^7.48^, located three residues upstream of the predicted **CRAC**, was expected to form hydrophobic interactions with cholesterol, whereas R328^7.55^ was expected to form hydrogen bonds with the hydroxyl group of cholesterol. However, for the F321^7.48^A β_2_AR mutant, the cholesterol effect on Tm was not statistically significant, while for the R328^7.55^A β_2_AR mutant, cholesterol still increased the Tm value. This effect may be attributed to stabilization of the bulk lipid phase induced by the mutation, rather than a specific disruption of cholesterol–protein interactions.

#### 2.3.6 β_1_AR: X-ray Crystallography

Experimental evidence supporting the potential allosteric inhibition of β_1_AR signaling by cholesterol has emerged from available X-ray structures.^196^ Comparative structural analysis has been performed using the X-ray structures of the β_1_AR in complex with the agonist isoprenaline (PDB ID 2Y03 ^197^) and partial agonist dobutamine (PDB ID 2Y00 ^197^) in which the receptor adopts an intermediate-active (inactive-like) conformation, alongside the fully active conformation structure of isoprenaline−β_1_AR–Nb80 (PDB ID 6H7J^196^).

These conformations display distinct differences in key elements of the canonical microswitch signaling network, including the PIF, DRY, and NPxxY motifs, as well as the conserved Y^5.58^. The intermediate-active conformation notably lacks the characteristic outward movement of TM6 required for full activation. In the intermediate-active structure (PDB ID 2Y03 ^197^) cholesterol-analog CHS is cocrystallized at the IC TM3/TM4/TM5 interface. This CHS molecule forms extensive, hydrophobic contacts with residues V129^3.40^, I137^3.48^, V160^4.44^, P219^5.50^, and several nearby valines (V172^4.56^, V202^EL2^, and V314^6.59^) and forms a hydrogen bond with E130^3.41^. In the structure of β_1_AR bound to dobutamine (PDB ID 2Y00 ^197^) two CHS molecules were resolved: one at the IC TM3/TM4/TM5 site and another at the IC TM2–TM4 **CCM**, consistent with the β_2_AR inactive-like structures. However, no CHS molecules were resolved in the fully activated β_1_AR conformation (PDB ID 6H7J).^196^

It has been proposed that cholesterol, or its analog CHS, inhibits activation by stabilizing the IC TM3–TM5 region, preventing the necessary conformational transitions within the microswitch network. Full receptor activation requires the outward movement of the IC portion of TM5 and the inward displacement of TM4.^196,197^ CHS interactions with E130^3.41^, I137^3.48^ may obstruct the inward motion of TM3, a key step for PIF motif activation.^196,197^ thus locking the receptor to the intermediate-active state. Moreover, steric hindrance may arise between the aliphatic tail of CHS and side chains of I214^5.45^ and I218^5.49^, as well as between the polar head of CHS and residues V160^4.44^ and T164^4.48^. These interactions likely hinder the conformational shift from the intermediate-active to the fully active state of β_1_AR. Consistent with observations in β_2_AR, no cholesterol molecules have been resolved in the experimental structures of β_1_AR ternary complexes.

#### 2.3.7 β_1_AR: NMR Spectroscopy

A general mechanism has been proposed through which cholesterol modulates the functional activity of β_1_AR using solution NMR spectroscopy.^198^ Specifically, high-pressure NMR studies have suggested that empty hydrophobic cavities within the β_1_AR structure, when occupied by cholesterol molecules, shift the equilibrium toward an inactive-like conformation. Within this framework, cholesterol acts as a negative allosteric modulator (NAM), thereby reducing the binding of the G protein.^198,199^

In these studies, β_1_AR was solubilized in a detergent and analyzed using high-pressure 2D [^15^N,^1^H] solution NMR spectroscopy, alongside X-ray crystallography of xenon-derivatized β_1_AR crystals. The NMR results demonstrated that the addition of CHS inhibits the formation of active conformation by interfering with the activation of conserved microswitches within the GPCR structure.^198^ This effect was accompanied by a reduction in the affinity for both isoprenaline and the G protein-mimicking Nb80 for β_1_AR as determined by ITC.^198^ Further supporting this model, the results in ref 200 suggested that the intermediate-active (inactive-like) conformation of the β_1_AR contains entirely vacant hydrophobic cavities near the ligand-binding site and within the receptor core, features absent in the receptor’s active state. The active conformation of β_1_AR exhibits a total volume approximately 100 Å^3^ smaller than that of the agonist-bound intermediate-active conformation. Using X-ray crystallography of xenon-derivatized β_1_AR crystals, the locations of these internal cavities were mapped. One of them is in direct contact with the cholesterol-binding pocket observed in the agonist-bound β_1_AR structure (PDB ID 2Y03 ^197^). It was therefore suggested that cholesterol functions as a NAM of agonist isoprenaline and inhibits the binding of G protein by filling the spaces between the TMs, which are required to be compressed in order the receptor to be activated by an agonist.^198^ Consequently, cholesterol inhibits G protein binding and stabilizes the intermediate-active conformation. Interestingly, the presence of cholesterol increased the affinity for the partial inverse agonist timolol but did not affect the binding of the full agonist isoproterenol.^198^ Displacement of cholesterol from these sites may suppress these cavities and promote the transition of β_1_AR from the agonist-bound intermediate-active conformation to the fully active state. This transition occurs under physiological pressure only upon binding of G protein or an Nb-mimetic to the IC side of the receptor, illustrating the cholesterol-dependent allosteric regulation of β_1_AR activation.

#### 2.3.8 Simulation Results of the Homo-Oligomerization of β_2_AR and β_1_AR

MD simulations have been employed to investigate the effect of cholesterol on the oligomerization of β_2_AR and β_1_AR. Thus, long-time-scale AA MD simulations reported in ref 149 revealed that cholesterol modulates the dimer interface of the β_2_AR through specific interactions at TM4, suggesting a cholesterol-driven stabilization of this interface.

Additionally, CG MD simulations were performed for both β_1_AR and β_2_AR to explore the role of hydrophobic mismatch in GPCR oligomerization.^201^ The simulations used the inactive-state crystal structures of β_1_AR (PDB ID 2VT4 ^189^) and β_2_AR (PDB ID 2RH1)^104^ as starting models. For β_1_AR, a high-density cholesterol-interaction site was identified within the IC TM2–TM4 **CCM** groove. In contrast to β_2_AR, however, no association of cholesterol was observed in the IC TM1/H8 groove, potentially due to differences in the amino acid composition lining this interface. Interestingly, the orientation of cholesterol interacting with the **CCM** site in β_1_AR was nearly identical to that observed in cocrystallized cholesterol molecules in inactive β_2_AR structures (PDB IDs 2RH1,^104^ 3D4S,^113^ 3NY8)^185^ as well as to the CHS molecule resolved in the β_1_AR structure (PDB ID 2Y00;^197^ see Figure 3).

The simulations further evaluated^201^ the energetic cost of residual hydrophobic mismatch—defined as the membrane’s incomplete accommodation of a transmembrane segment’s hydrophobic length. For β_2_AR monomers, the highest energy penalties were found at TM1, TM4, and TM5, suggesting that dimerization involving the TM1 and TM4/TM5 regions may relieve this mismatch and thus drive oligomer formation. In contrast, β_1_AR, despite its high sequence homology to β_2_AR, exhibited significant hydrophobic mismatch only at TM1, favoring dimerization at TM1/TM1 interfaces rather than higher-order oligomerization. Notably, these simulations indicated that cholesterol does not significantly influence the oligomerization behavior of β_1_AR or β_2_AR under the conditions tested.^201^

### 2.4 A_2A_R

#### 2.4.1 Biochemical Assays

Conflicting data have been reported regarding the modulation of A_2A_R by cholesterol, particularly in terms of ligand binding. The functional assay results also need careful interpretation.^40,145,156^ In ligand binding studies, inconsistent results have been observed. For instance, A_2A_R retained the ability to bind the selective inverse agonist [^3^H]­ZM241385 even in *E. coli* membranes, which lack cholesterol.^202^ In another study using intact C6 glioma cells, cholesterol depletion via MβCD enhanced ZM241385 binding affinity, suggesting that cholesterol may inhibit antagonist binding.^203^ In contrast, in detergent micelle systems, radioligand binding was abolished when A_2A_R was purified without CHS, indicating a potential stabilizing role for cholesterol in this context.^204,205^ Additional radioligand binding experiments with A_2A_R incorporated into phospholipid nanodiscs containing POPC and varying cholesterol levels showed insignificant correlation between antagonist binding affinity and cholesterol concentration.^206^ A modest positive correlation was observed between cholesterol concentration and agonist binding affinity, although the effect did not exceed 1 order of magnitude. Likewise, studies in HEK cells reported no significant effect of cholesterol on ligand binding to A_2A_R.^207^

Results from functional assay experiments are more consistent, although they are nuanced. In mammalian cells expressing A_2A_R, cholesterol depletion reduces agonist-induced Gαs coupling as measured by cAMP production.^207^ Consistent findings were reported using GTP hydrolysis assays, where the addition of cholesterol to POPC nanodiscs increases nucleotide exchange.^206^ However, this effect was attenuated when anionic phospholipids such as POPS or POPG were incorporated into the membrane, indicating that membrane lipid composition can modulate the influence of cholesterol on A_2A_R function.^208^ Specifically, in POPC/POPG nanodiscs, cholesterol was found to weakly enhance basal A_2A_R signaling while concurrently reducing agonist potency.

#### 2.4.2 Results from Bioinformatics Analysis–X-ray Crystallography

The bioinformatics analysis conducted in refs 51,53,159 revealed the presence of the IC TM2–TM4 **CCM** and IC TM7 **CRAC** motifs conserved between the studied class A GPCRs (see Table S1). A substantial number of X-ray structures of A_2A_R-antagonist complexes (∼54) have been solved, with a significant portion (∼41, or ∼76%^45^ of the cholesterol-containing structures of GPCRs of all classes) containing cocrystallized cholesterol molecules.

In the inactive conformation of A_2A_R, within antagonist-bound complexes, three or four cholesterol molecules are typically resolved at the EC TM2/TM3 (TM2/EL1/TM3), EC TM5/TM6 (TM5/EL3/TM6), and EC TM6/TM7 regions, as depicted **in** Table 1 and shown in Figure 4 (see, for instance, PDB IDs 3EML,^209^ 4EIY,^210^ and other structures in refs 205,209,211–219 ).

**Figure 4:**
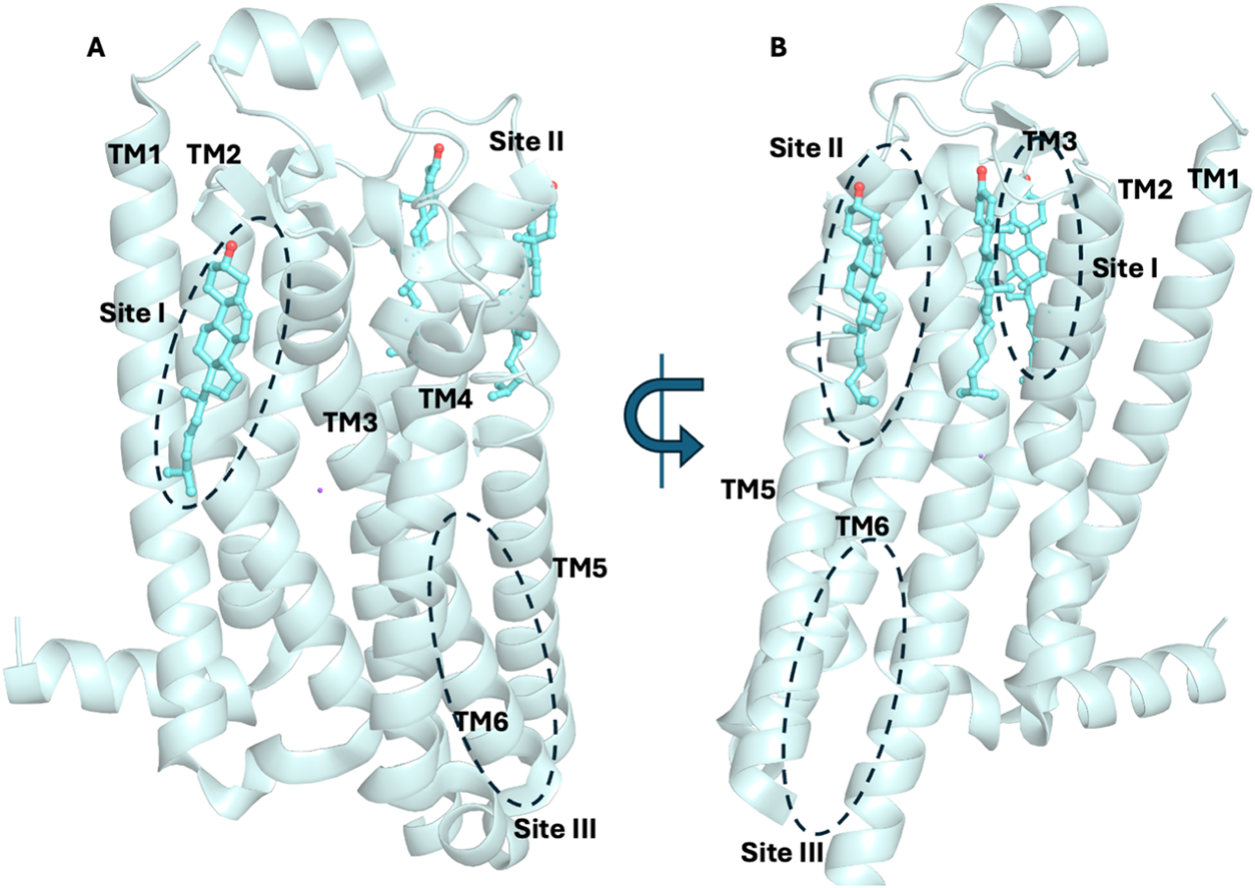
(A) Crystal structure of inactive A_2A_R (PDB ID 4EIY^210^) highlighting cocrystallized cholesterol molecules. (B) The same structure is shown rotated ∼180° along the 7TM bundle axis to provide a complementary view. Cholesterol molecules are shown as sticks. Three key cholesterol-interaction sites are marked: **Site I**, located at the interface of EC TM2, EC EL1, and EC TM3 (EC TM2/TM3). **Site II** (EC TM5/TM6 and EC TM6/TM7): spans across EC TM5, EL3, TM6, and TM7. **Site III** (IC TM5/TM6): identified through CG MD simulations as a potential intracellular cholesterol-binding site. All three interaction regions (**Sites I–III**) are enclosed with black dotted lines.

#### 2.4.3 MD Simulations–Functional Assays

The MD simulations with A_2A_R provided intriguing results and suggested important cholesterol-interaction motifs not observed with experimental methods. Since structural biology results demonstrated that cholesterol functions as a stabilizer of A_2A_R,^206^ and biochemical data indicated that signaling of A_2A_R through coupling with Gαs is reduced with cholesterol depletion,^207^ MD simulations were performed to study interactions of the inactive conformation of A_2A_R embedded in phospholipid bilayers with cholesterol. The nature of cholesterol interaction sites varied depending on the resolution of the simulation method. For instance, a favorable interaction of cholesterol with EC TM2/TM3 (TM2/EL1/TM3), which is the experimentally observed **Site I**, was identified with CG MD simulations.^131,220^ Also, interaction with EC TM6/TM7, which is the experimentally observed **Site II**, was revealed with both AA MD simulations^57,221,222^ and CG MD simulations,^131,220,221^ but the EC TM5/TM6 (TM5/EL3/TM6), which is **Site III**, not observed experimentally, was calculated only with CG MD simulations^15,131,221^ (Figure 4).

Notably, high-residence-time cholesterol interaction sites of the inactive A_2A_R were calculated using CG MD simulations in ref 131 (Figure 5A) correspond well with **Sites I–III**. An additional predicted site along EC TM1 was also identified. Following back-mapping, the AA model for **Site II** is illustrated in Figure 5B.

**Figure 5:**
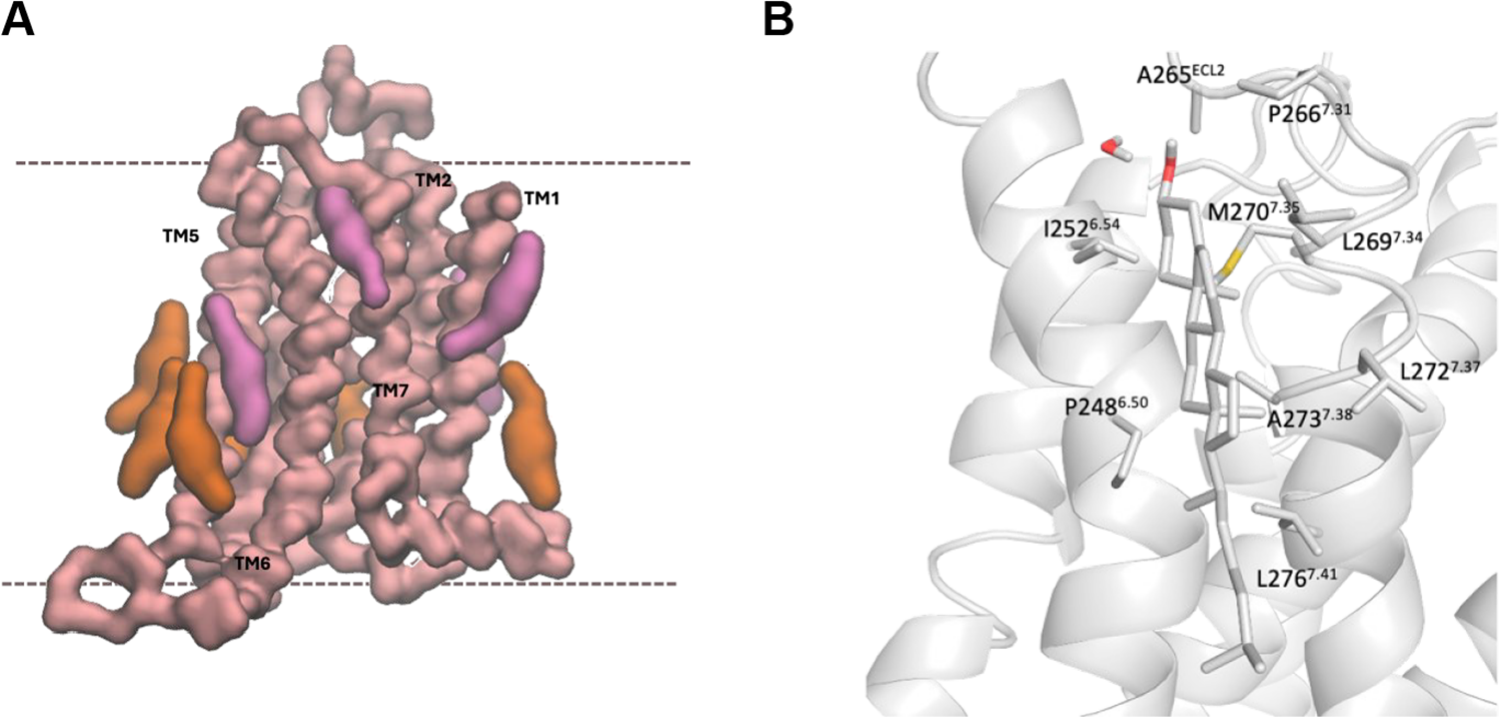
(A) Cholesterol-interaction sites on inactive A_2A_R embedded in a plasma-mimetic membrane, identified via CG MD simulations.^131^ The receptor is shown as a pink surface. Cholesterol-interaction sites are depicted as surface patches in orange (residence time <1 μs) and mauve (residence time >1 μs), indicating site stability. (B) Back-mapped atomistic model of a representative snapshot from CG MD simulations, highlighting the cholesterol-binding site at the EC TM6/TM7 region (**Site II**), which corresponds to an experimentally observed site. The receptor is shown as a white cartoon; cholesterol and key interacting side chains are shown as sticks.

AA MD simulations^223^ using the inverse agonist ZM241385-A_2A_R complex (PDB ID 3EML^209^) embedded in POPC bilayers, demonstrated that, in the absence of cholesterol, TM2 of the apo-receptor was highly unstable. In contrast, the presence of cholesterol stabilized TM2 via direct interaction with its EC region. Two additional cholesterol molecules were found associated with IC TM1–TM3, suggesting they contribute to receptor stabilization.

The AA MD simulations^57^ with the intermediate-active state of A_2A_R, using as starting structure the crystal structure of agonist adenosine-A_2A_R complex (PDB ID 2YDO^224^) embedded in POPC/cholesterol bilayers or CG and AA MD simulations with the intermediate-active state of A_2A_R, using as starting structure the crystal structure of agonist NECA-A_2A_R complex (PDB ID 2YDV^224^) embedded in POPC/cholesterol bilayers showed three cholesterol molecules in direct interaction with A_2A_R (see Figure 4) as observed in the X-ray structure of A_2A_R-ZM241385 (PDB ID 4EIY^210^).^57^ These simulations reproduced^57^ the cholesterol-binding pattern observed in the crystal structure of ZM241385–A_2A_R (PDB ID 4EIY^210^), with two cholesterol molecules occupying **Site I** (EC TM2/TM3) and **Site II** (EC TM6/TM7) (see Figure 4).

The AA MD simulations of intermediate-active A_2A_R in POPC/cholesterol bilayers in ref 221 indicated that a cholesterol molecule resides in a cavity between IC TM2–TM4 within a **CCM**. Cholesterol interacts with residues Y43^2.41^, S47^2.45^, K122^4.43^, I125^4.46^, and W129^4.50^ (Figure 6) in the IC membrane leaflet.^206^ Residues Y43^2.41^ and K122^4.43^ are positioned to form hydrogen bonds with the hydroxyl group of cholesterol (Figure 6), while I125^4.46^ engages in hydrophobic interactions.^206^ As previously shown, the most conserved residue of the IC TM2–TM4 **CCM**, W129^4.50^, forms a ring stacking interaction with cholesterol via its indole side chain, generating steric repulsion that facilitates the disruption of the E228^6.30^–R102^3.50^ ionic lock, thus favoring activation (Figure 6C,D), in agreement with the functional data discussed in Section 2.4.1.^206,207^

**Figure 6:**
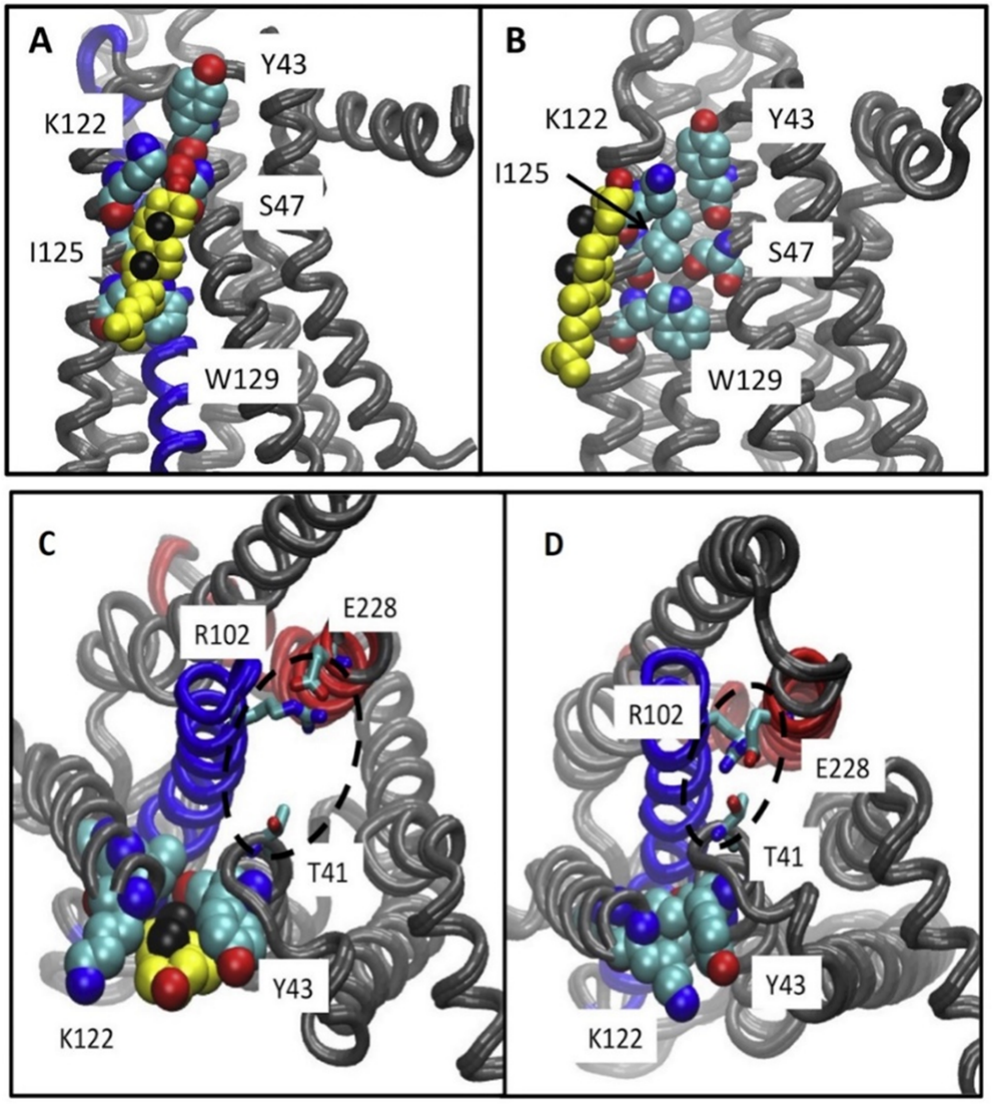
Prediction of cholesterol-interaction sites from AA MD simulations using the intermediate-active structure of A_2A_R bound to NECA (PDB ID 2YDO^224^) embedded in cholesterol-containing bilayers.^221^ (A) A cholesterol molecule (shown as van der Waals spheres) binds within the IC TM2–TM4 **CCM**, interacting with residues Y43^2.41^, S47^2.45^, K122^4.43^, I125^4.46^, and W129^4.50^. (B) A cholesterol molecule interacting along IC TM4 of the same **CCM**, contacting K122^4.43^ and I125^4.46^. The cholesterol’s presence in this site may disrupt the E228^6.30^–R102^3.50^ ionic lock, thereby increasing the receptor’s population in the signaling state. (C) Representative conformation of the disrupted ionic lock when cholesterol is present in the IC TM2–TM4 **CCM**. Notably, T41^2.39^ (located half a helix-turn from Y43^2.41^ of the **CCM**) is absent from the interaction network. (D) In the absence of cholesterol, a stable ionic lock is formed between R102^3.50^ and E228^6.30^, with additional participation from T41^2.39^, as observed in the crystal structures of inactive A_2A_R. Panels C and D were reproduced from ref 207.

A subsequent functional assay study^207^ of A_2A_R mutants W129^4.50^A and K122^4.43^A showed decreased signaling activity compared to wild-type A_2A_R, supporting the involvement of cholesterol, which—according to MD simulations—fails to bind effectively to the IC TM2–TM4 CCM in these mutants. The K122^4.43^A mutation caused only a ∼2-fold reduction in both agonist and antagonist affinity^204^ and is also part of a thermostabilized, antagonist-preferring variant of A_2A_R.^225^ In contrast, the W129^4.50^A mutant showed a modest reduction in ligand binding affinity.^206,207^ Cholesterol depletion by MβCD in cells expressing A_2A_R K122^4.43^A or A_2A_R W129^4.50^A resulted in reduced Gαs coupling and cAMP levels.^207^ While the K122^4.43^A mutant responded similarly to the wild-type receptor, the W129^4.50^A mutant showed no additional reduction upon cholesterol depletion, suggesting a loss of functional interaction with cholesterol at this site.

Additional long-time scale AA and CG MD simulations^207^ were performed using various A_2A_R conformations, including inactive states bound to an inverse agonist or antagonist (PDB IDs 4EIY,^211^ 3RFM^226^), intermediate-active conformations of A_2A_R bound to agonists (PDB IDs 2YDV,^224^ 3QAK^227^) and an apo-state derived by ligand removal. These simulations consistently identified a cholesterol-binding site at the IC TM2–TM4 **CCM** in the intermediate-active conformation, resembling the same CCM observed in the inactive β_2_AR (e.g., PDB ID 3D4S^113^). While this site is not resolved in experimental A_2A_R structures, functional assays^207^ confirm the critical role of W129^4.50^ as a cholesterol-binding residue. The absence of this site in crystallographic data may stem from (a) GPCR flexibility, which can result in different conformations between crystal and simulated structures, and (b) receptor-stabilizing modifications introduced for crystallization—such as the K122^4.43^A mutation—that may alter cholesterol binding.^225^

Other CG MD simulations,^131^ using the experimental structure of active A_2A_R (PDB ID 5G53 ^228^) as starting structure, predicted a cholesterol-binding site in IC TM7 **CRAC**, in agreement with bioinformatic analyses.^51,53,159^

Cholesterol interaction energies were estimated at each site in the AA MD simulations^57^ using an inverse Boltzmann approach and fell within the *kT* range, confirming the weak association of cholesterol with the receptor. Consistent US/PMF calculations^54–58,131^ showed cholesterol interacts with both active and inactive A_2_AR with binding free energies of ∼1–4 *kT*, depending on the site’s depth.

Multiple long AA MD simulations^118,207,222^ and CG MD simulations^15,56,131,207,220^ of inactive and intermediate-active, and active A_2A_R in POPC/cholesterol and plasma mimetic membranes^15,131^ predicted many additional cholesterol sites. For both A_2A_R and β_2_AR, the EC TM6/TM7 and IC TM1/H8 sites were consistently observed. Additionally, CG MD simulations of A_2A_R in the active state,^220,229^ inactive state^56,220,229^ and intermediate-active state^56,220^ revealed distinct long-standing cholesterol interaction sites. Notably, EC site binding was more pronounced in the intermediate-active state^220^ and active state^220,229^ compared to the inactive state.^220,229^ Moreover, the interaction time of cholesterol at IC TM6/TM7 was reduced in the active conformation due to competition with phosphatidylinositol 4,5-bisphosphate (PtdIns­(4,5)­P_2_; abbreviated also as PI­(4,5)­P_2_ or PIP2) molecules.^220^

Finally, AA MD simulations of the A_2A_R in its complex with inverse-agonist ZM241385 (PDB ID 3EML^209^) embedded in POPC/cholesterol bilayer showed cholesterol entering the 7TM core via the TM5/TM6 interface^118^ a pathway also supported by other AA MD simulations.^221^

#### 2.4.4 ^19^F NMR Spectroscopy

^19^F solution NMR studies explored the effect of cholesterol on ligand binding (agonist or antagonist) and on the conformational equilibrium of A_2A_R, revealing shifts in the population of active-like states as well as new cholesterol-interaction sites. The ^19^F solution NMR spectroscopy study examined the interaction of cholesterol with A_2A_R, supported by functional assays based on the GTP hydrolysis catalyzed by the receptor.^208^ The study used a truncated A_2A_R construct (V229^6.31^C Δ317R; residues 2–317) labeled with ^19^F at the cytoplasmic half of TM6, a region sensitive to activation-linked conformational changes. The receptor was reconstituted in POPC/POPG (3:2) nanodiscs with varying cholesterol concentrations (0–13%) along with purified Gαs-short/β1γ2 proteins. The functional assays (Section 2.4.1) showed that cholesterol slightly enhances basal signaling of A_2A_R by promoting a shift toward the active state, while reducing the potency of the NECA agonist. The ^19^F NMR spectra of the apo-A_2A_R or inverse agonist (ZM241358) bound A_2A_R at 293 K displayed two predominant inactive conformations, **S1** ^**TM6**^, **S2** ^**TM6**^ (one with a broken ion-lock), and the three active-like states, **A1** ^**TM6**^-**A3** ^**TM6**^. **A1** ^**TM6**^ conformation dominated upon NECA binding and increased further with Gαβγ addition. **A2** ^**TM6**^ likely represents a preactive state, while **A3** ^**TM6**^ conformation, stabilized by Gαβγ binding, corresponds to a precoupled state. At ≤ 4% cholesterol, no spectral change was detected. At 13%, subtle effects emerged, including line broadening and a 0.09 ppm downfield shift of the **A1** ^**TM6**^ peak. This shift was absent in the presence of both agonist and G protein, suggesting that cholesterol weakly stabilizes the active-like ensemble. Alternatively, cholesterol’s influence may occur indirectly by modulating membrane fluidity. The use of fluorinated cholesterol analogs confirmed only transient and low-affinity interactions with A_2A_R, indicating no strong direct allosteric modulation. This work also highlighted limitations of the Δ317R A_2A_R construct, as truncation affects G protein binding. SPR assays confirmed that deletion of the C-terminal tail reduces Gs protein association.^230^ Therefore, full-length receptor studies may yield different results.

In a related study, ^19^F solution NMR experiments were performed at 280 K using A_2A_R A289^7.54^C labeled at the cytoplasmic half of TM7.^206^ A_2A_R was reconstituted in nanodiscs containing POPC alone or with the addition of anionic POPS or POPG, with cholesterol concentrations ranging from 0 to 15%. Addition of POPS or POPG significantly increased the GTPase activity of agonist-bound A_2_AR and the population of active-like states, confirming previous observations.^231^ The ^19^F-NMR spectra of the A_2A_R A289^7.54^C in complex with the antagonist ZM241385 showed minimal changes with 5% cholesterol, consistent with radioligand binding studies in HEK cells,^206,207^ as opposed to the results from radiolabeled experiments in ref 232. In POPC/POPG/13% cholesterol nanodiscs, more noticeable ^19^F NMR shifts were observed. These data, along with GTPase and cAMP-based assays, suggest that cholesterol directly interacts with A_2A_R in zwitterionic membranes like POPC.^208^ Furthermore, ^19^F NMR studies with mutant A_2A_R R291^7.56^Q confirmed the presence of an intracellular TM7 **CRAC** motif (Y283^7.48^, Y288^7.53^, R291^7.56^) as predicted by bioinformatics^51,53,159^ and CG MD simulations,^131^ though not seen in crystal structures. The W129^4.50^I mutant showed that cholesterol interaction with W129^4.50^ in the **CCM** contributes to structural stability, while R291^7.56^ affects signaling. The addition of cholesterol caused only subtle shifts in the ^19^F-NMR spectra of A_2A_R A289^7.54^C in nanodiscs containing the anionic membrane POPC/POPS or POPC/POPG, aligning with the functional assays in refs 206,208 . This suggests that the effect of cholesterol is attenuated by the presence of an anionic lipid, indicating a complex interplay between cholesterol and membrane composition in A_2A_R regulation.

The conformational equilibrium of the A_2A_R (residues 1–316) in POPC/POPS (70:30) nanodiscs was studied in a subsequent work using variable-temperature ^19^F-NMR spectroscopy with labeled A289^7.54^C A_2A_R.^233^ Upon addition of 5% cholesterol, the active-state (**A1** ^**TM7**^) population increased from 47 to 67% at 298 K. However, in POPC/POPG nanodiscs, cholesterol slightly decreased the **A1** ^**TM7**^ population from 70 to 65%. This weak allosteric modulation suggests that POPG attenuates cholesterol’s impact. These results reinforce the notion that cholesterol and anionic lipids may have complementary roles in regulating A_2A_R activation, consistent with MD simulations^234^ and prior experimental studies.^206,208^

### 2.5 Serotonin Receptors 1A and 2A

#### 2.5.1 Results from Bioinformatics Analysis

Three **CRAC** motifs located in EC TM2, IC TM5, and IC TM7/H8, as well as IC TM2–TM4 **CCM** motif, have been reported in previous studies (see also Table S1).^51,53,159^ Additionally, the studies in ref 235 identified an evolutionarily conserved **CRAC** motif of the 5-HΤ_1A_R in various species, including residues L220^5.63^–R231^5.74^ (with the corresponding amino acid sequence **L**-(LMLVL)-**Y**-(GRIF)-**R**; underlined and residues that fit a **CRAC** motif are shown in bold).

#### 2.5.2 5-HT_1A_R: Biochemical Assays

5-HΤ_1A_R is a representative GPCR implicated in neuronal development and neuropsychiatric disorders, including depression and anxiety. This receptor was among the first GPCRs for which cholesterol and membrane content were shown to modulate organization, oligomerization, dynamics, function, and ligand binding.^236^ These studies provided evidence that cholesterol is essential for both ligand binding and receptor-G protein coupling of this key neurotransmitter receptor in native membranes.^237–239^ The interaction of cholesterol with a **CRAC** motif appears to be important for receptor activation. Moreover, multiple studies have explored the localization of 5-HT_1A_R within lipid rafts, a factor crucial for its function in neuronal cells.^195,237–249^

Significantly, approximately 25% of the body’s total cholesterol content is in the central nervous system (CNS). Cholesterol acts as a PAM of the 5-HΤ_1A_R; its depletion inhibits the serotonin-induced reduction of cAMP levels, reduces agonist binding, and disrupts dynamics and organization in neuronal cells.^240–242,245^ In ref 250 solubilization of hippocampal 5-HΤ_1A_R using the zwitterionic detergent 3-[(3-cholamidopropyl)­dimethylammonio]-1-propanesulfonate (CHAPS) led to cholesterol loss, resulting in decreased specific ligand binding. Reintroduction of cholesterol into solubilized membranes restored ligand binding to the receptor. Furthermore, cholesterol has been shown to enhance the stability of 5-HT_1A_R in giant unilamellar vesicles (GUVs),^99^ and to function as a PAM based on a GTP/GDP exchange assay.^244^

In ref 251 cholesterol depletion or membrane enrichment with cholesterol beyond basal levels resulted in an increase and a reduction, respectively, of basal receptor signaling. These findings indicate that an optimal membrane cholesterol level is essential for maintaining 5-HΤ_1A_R activation, highlighting the critical role of cellular cholesterol homeostasis.^251^

The modulatory role of cholesterol on the ligand-binding activity and receptor-G protein interaction of bovine hippocampal 5-HT_1A_R has been demonstrated by cholesterol depletion from native membranes using MβCD.^240^ Specifically, the selective removal of cholesterol from hippocampal membranes with MβCD resulted in a concentration-dependent reduction in the specific binding of the agonist 8-OH-DPAT to 5-HT_1A_Rs. In cholesterol-depleted membranes, the receptors exhibited approximately 2.5-fold lower sensitivity to GTP-γ-S, indicating a reduced receptor/G-protein interaction. Replenishing the cholesterol-depleted membranes with cholesterol partially restored agonist-binding activity, supporting the specificity of the effect of cholesterol depletion.

The function of 5-HT_1A_R has also been studied under lipid oxidation conditions (e.g., cholesterol oxidation), which are associated with various diseases, such as Alzheimer’s disease, multiple sclerosis, Parkinson’s disease (PD), and Huntington’s disease.^252^ GUVs composed of the polyunsaturated 1-palmitoyl-2-linoleoyl-*sn*-glycero-3-phosphocholine (PLinPC), its oxidation product 1-palmitoyl-2-(9′-oxo-nonanoyl)-*sn*-glycero-3-phosphocholine (PoxnoPC), and 5-HT_1A_R were generated and compared with GUVs containing 5-HT_1A_R in lipid mixtures subjected to prolonged atmospheric oxidation.^253^ Results from a GDP/GTP exchange assay indicated that the activity of 5-HT_1A_R was substantially enhanced in bilayers comprising oxidized lipids.^253^

In ref 243, mutants of key amino acids in the proposed EC TM2 **CRAC** motif (which includes Y96^2.63^ and K101^2.68^) and the IC TM5 **CRAC** motif of the 5-HT_1A_R (Y215^5.58^, R220^5.63^) were generated. The three mutants -Y96^2.63^S, Y215^5.58^S, R220^5.63A^- exhibited inhibition of cAMP-based signaling upon cholesterol depletion, like wild-type 5-HT_1A_R, suggesting that these residues are not directly involved in mediating the receptor’s cholesterol sensitivity. However, mutation of residue K101^2.68^ in the EC TM2 **CRAC** motif abolished the functional sensitivity of the 5-HT_1A_R to membrane cholesterol. This finding suggests that K101^2.68^ is crucial for cholesterol binding. The interaction of cholesterol at EC TM2 **CRAC** can favor the conformational change of 5-HT_1A_R to the active state.

FRAP was applied using 5-HT_1A_R-EYFP, i.e., 5-HT_1A_R tagged with the green/yellow fluorescent protein EYFP, to investigate how cholesterol levels influence the lateral mobility of the receptor in the plasma membrane of living cells.^241^ It was found that treatment of membranes with MβCD significantly reduced 5-HT_1A_R mobility, suggesting that cholesterol plays a crucial role in maintaining the receptor’s dynamic behavior within the membrane. In another study, 5-HT_1A_R was reconstituted in unilamellar protein vesicles (GUPs), and it was shown^244^ that membrane lipid composition and physicochemical properties strongly influence 5-HT_1A_R function. Results using fluorescent probes demonstrated that increasing membrane order and curvature, induced by cholesterol or other sterols or SM, enhanced 5-HT_1A_R signaling. This enhancement was confirmed via a GDP/GTP exchange assay and was further amplified as membrane elastic curvature stress increased.^244^ These findings are consistent with prior studies demonstrating the segregation of 5-HT_1A_R into the lipid raft domain.^240–242,245^ The results indicate that cell signaling functions may be modulated by alterations in membrane composition due to aging, disease, or diet. Moreover, the methodology described in ref 244 provides a valuable approach for assessing lipid-related factors that influence GPCR activity—factors that may not be easily investigated through in vivo or conventional experimental systems.

The effect of chronic cholesterol depletion induced by mevastatin on the function of 5-HT_1A_R expressed in CHO cells was explored.^245^ Mevastatin treatment led to a decrease in the diffusion coefficient and an increase in the mobile fraction of the receptor, as determined by fluorescence recovery after photobleaching studies. In another study, the impact of chronic cholesterol depletion through statin treatment on 5-HT_1A_R signaling was investigated.^254^ It was shown that lovastatin treatment reduced cAMP signaling, attributed both to a decrease in cholesterol levels and to actin cytoskeleton polymerization. Confocal microscopy and flow cytometry revealed that 5-HT_1A_R undergoes clathrin-mediated endocytosis upon agonist stimulation and traffics through the endosomal recycling route.^254^ Similarly, in ref 255 statin-induced chronic cholesterol depletion was shown to switch the receptor’s endocytic pathway from clathrin- to caveolin-mediated endocytosis. Under these conditions, a significant portion of internalized receptors is rerouted toward lysosomal degradation.

Additionally, 5-HT_1A_R exhibits tissue- and cell-type-specific activity. For example, agonist-induced internalization has been observed in neurons originating from the dorsal raphe nucleus, but not in hippocampal neurons.^256,257^ This discrepancy might be attributed to differences in lipid composition between neuronal membranes,^258,259^ with cholesterol shown to inhibit internalization of the agonist- and Gi protein-bound 5-HT_1A_R.

#### 2.5.3 5-HT_1A_R: Cryo-EM

Cholesterol interaction sites located near the OBS may significantly influence the affinity of orthosteric ligands, especially in GPCRs localized within cholesterol-rich raft domains. Cholesterol acts likely as a PAM, enhancing 5-HT_1A_R signaling by binding near the OBS and stabilizing agonist binding.

Cryo-EM was employed to resolve the structures of several 5–HT_1A_R–Gi complexes, including apo-5-HT_1A_R–Gi, serotonin–5-HT_1A_R–Gi, aripiprazole-5-HT_1A_R–Gi, serotonin–5-HT_1D_R serotonin–5-HT_1E_R, as well as BRL-54443-5-HT_1F_R complexes.^260^ In all five 5-HTR–Gi complexes, phosphatidylinositol 4-phosphate (PtdIns4P) was identified as the predominant phospholipid at the 5-HT_1A_R–Gi protein interface.^260^ The cryo-EM structures of the active 5-HT_1A_R, in the aripiprazole–5-HT_1A_R–Gi complex (PDB ID 7E2Z^260^) and the serotonin–5-HT_1A_R–Gi complex (PDB ID 7E2Y^260^), revealed ten cholesterol molecules in addition to the bound PtdIns4P, three of which are located near the OBS (Figure 7). These cholesterol interaction sites may contribute to the well-established role of cholesterol in the functional regulation of 5-HT_1A_R.^237–247^ Notably, one cholesterol molecule enters a cavity between EC TM1/TM7, acting as a structural “glue” that stabilizes the interface between these two helices (Figure 7A).

**Figure 7:**
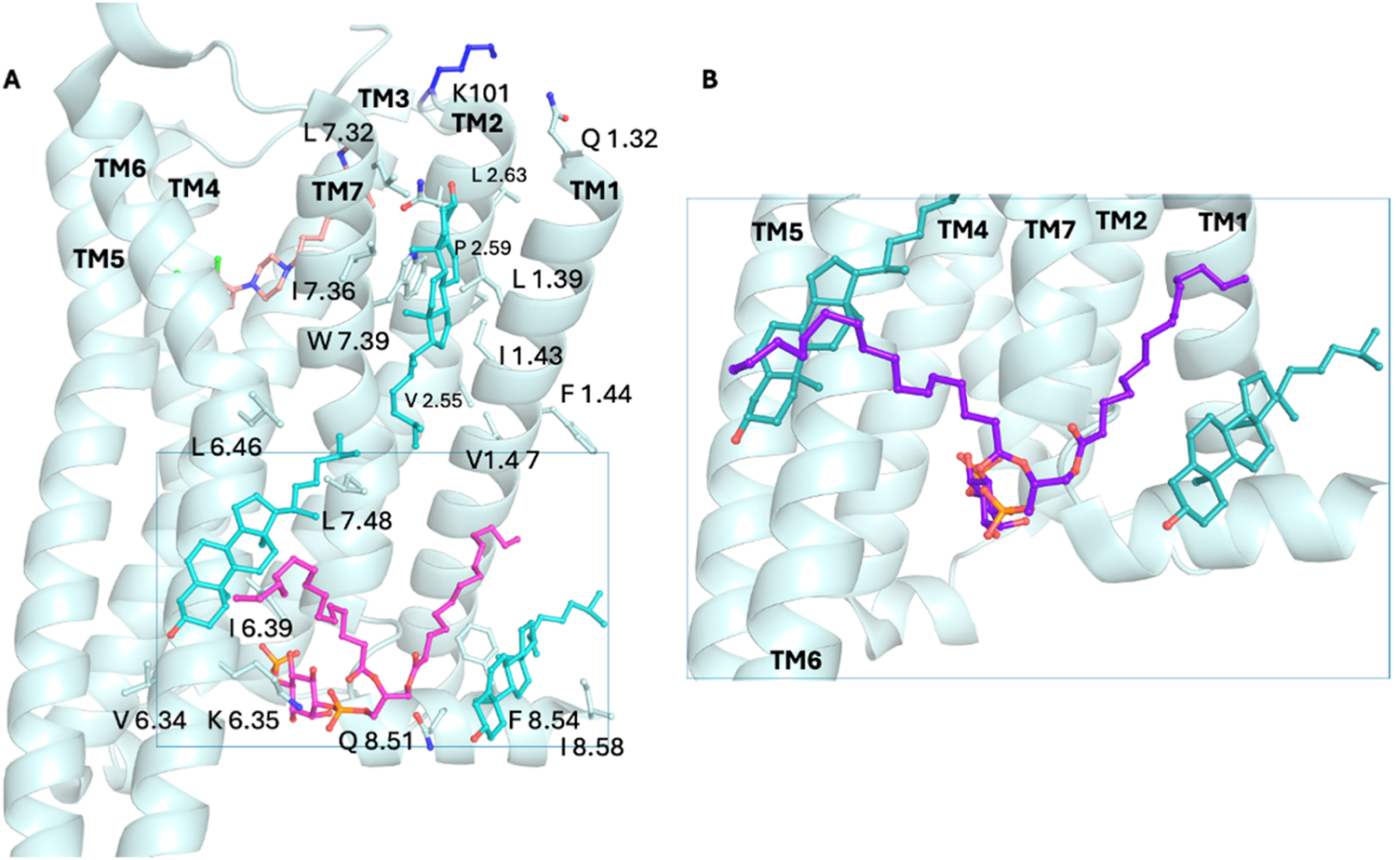
Cryo-EM structure of the aripiprazole–5-HT_1A_R–Gi protein complex (PDB ID 7E2Z^260^), shown with the Gi part omitted for clarity. Three cholesterol molecules are resolved in the structure and depicted with their carbon frameworks in light blue sticks. One PtdIns4P molecule is illustrated in purple sticks, and the orthosteric ligand aripiprazole is shown in pink sticks. Notably, one cholesterol molecule occupies a cleft at the interface EC TM1/TM7. This interaction is proposed to contribute significantly to the formation and stabilization of OBS.

In the structures of serotonin–5-HT_1A_R–Gi complex (PDB ID 7E2Y^260^) and the apo–5-HT_1A_R–Gi complex (PDB ID 7E2X^260^), an IC TM2–TM4 **CCM** is also observed. It would be of interest to investigate whether this **CCM** is maintained in inactive-like conformations of 5-HT_1_AR, given that the relative positions of TM2, TM4, and TM5 are expected to differ. The specificity of aripiprazole for 5-HT_1A_R is partly determined by the positioning of TM1 and TM7 near its quinolinone moiety, which is stabilized by cholesterol that shapes the ligand-binding pocket. This structural stabilization may explain the higher affinity of aripiprazole for 5-HT_1A_R compared to 5-HT_1D_R and 5-HT_1E_R. In contrast, in the structures of 5-HT_1B_R bound to the agonist donitriptan (PDB ID 6G79 ^261^) or to the antagonist methiothepin (PDB ID 5 V54 ^260^), as well as in the structures of 5-HT_1D_R and 5-HT_1E_R, no cholesterol molecule was observed at this site. Furthermore, in the cryo-EM structures of 5-HT_1A_R, both cholesterol and PtdIns4P directly interact with the receptor (Figure 7), providing a structural rationale for previous findings that both cholesterol and PLs modulate 5-HT_1A_R signaling^236–238^ and ligand binding.^236^ Mutagenesis of the PtdIns4P-binding residues in 5-HT_1A_ (R134^3×50^A, K345^6×35^A, and K405^8×48^A) led to reduced Gi protein activation and abolished the regulatory function of PtdIns4P.

Research has been conducted to discover drugs that take advantage of biased signaling by 5-HT_1A_R. In a study agonist ST171 was discovered, and cryo-EM studies were performed to interpret its interesting biological properties compared to agonist befiradol. Befiradol is a selective 5-HT_1A_R agonist and drug candidate that has antinociceptive properties comparable to clinically used opioids. Befiradol and serotonin can trigger recruitment of both Gs, Gi, and β-arr. On the other hand, ST171 revealed highly potent and functionally selective Gi/o signaling without Gs activation and marginal β-arr recruitment. ST171 was effective in acute and chronic pain models. In the cryo-EM structures of the complexes agonist ST171–5-HT_1A_R–Gi1 (PDB ID 8PJK^262^), agonist befiradol–HT_1A_R–Gi1 (PDB ID 8PKM^262^), agonist befiradol–HT_1A_R–Gs (PDB ID 9GL2 ^262^) was shown that befiradol and ST171 were found to be bitopic 5-HT_1A_R agonists, i.e., bind to the OBS and allosteric sites. The cryo-EM structures of ST171 bound to 5-HT_1A_R in complex with the Gi protein, compared to the canonical agonist befiradol bound to complexes of 5-HT_1A_R with Gi or Gs revealed that the ligands occupy different exosites that can affect the conformation of the cytoplasmic region, as suggested by MD simulations, causing engagement with different transducers and relevant signaling. Interestingly, a cholesterol molecule was also observed at EC TM7 close to the OBS (as discussed previously; see Figure 7A) in the cryo-EM structures of the complexes of ST171 or befiradol (PDB IDs 8PJK^262^ or 8PKM^262^ and 9GL2,^262^ respectively).

#### 2.5.4 5-HT_1A_R: MD Simulations

CG and AA MD simulations have highlighted the importance of cholesterol interactions with 5-HT_1A_R, supporting the presence of the key EC TM2 **CRAC** motif, which was also identified through sequence alignment analyses (see Table S1).^51,53,159^ Additional cholesterol-interaction sites have also been proposed.

Possible favorable cholesterol binding sites on 5-HT_1A_R were explored using CG MD simulations^152,263–265^ in POPC/cholesterol bilayers. These simulations employed homology models of the receptor based either on the X-ray structure of β_2_AR, which includes cocrystallized cholesterol molecules, or on the X-ray structure of the 5-HT_1B_R bound to the antimigraine agonist ergotamine (PDB ID: 4IAR^266^). Several cholesterol interaction sites were predicted in both membrane leaflets.^152,263^ Notably,^152^ one cholesterol interaction-site colocalized with the IC TM2–TM4 **CCM** motif identified in the cryo-EM structures of the fully activated 5-HT_1A_R and another with the IC TM5 **CRAC** motif, in agreement with the bioinformatics predictions discussed in Section 2.5.1.^53,235^ In ref 263 CG MD simulations demonstrated the presence of cholesterol significantly enhanced the stability of the receptor and its complexes with ligands. Furthermore, CG MD simulations in ref 265 indicated that the oligomerization of 5-HT_1A_R is lipid-dependent. Two primary dimer interfaces were identified, involving TM1/TM2 and TM4-TM6. These simulations underscored the critical role of cholesterol in modulating both the stability and the conformational flexibility of these dimeric interfaces.^265^

AA MD simulations of 5-HT_1A_R^267^ and 5-HT_2A_R^268^ as a function of cholesterol composition, have revealed opposite effects on their conformational flexibility; cholesterol was found to reduce the conformational flexibility of 5-HT_1A_R,^267^ whereas it increased that of 5-HT_2A_R.^268^

In addition to functional studies highlighting the importance of K101^2.68^ for cholesterol association with the EC TM2 **CRAC** motif identified via bioinformatic analysis (see Section 2.5.1),^243^ 2 μs AA MD simulations were performed in ref 243 on the serotonin–5-HT_1A_R complex. A homology model of 5-HT_1A_R generated from the closely related 5-HT_1B_R structure (PDB ID 6G79 ^261^) was used. The simulations (Figure 8) revealed that cholesterol occupies a groove at EC TM1/TM2, consistent with cryo-EM observations (Figure 7). In this region, cholesterol frequently interacts with a **CRAC** motif near K101^2.68^, being present at the site for at least 20% of the simulation time. Cholesterol is stabilized via polar interactions with Q36^1.32^ and K101^2.68^, and through extensive hydrophobic interactions (Figure 8B). This favorable interaction with K101^2.68^ tethers EL1 into a defined conformation by restricting its flexibility. These results suggest that the functional sensitivity of 5-HT_1A_R to membrane cholesterol may stem from cholesterol-induced stabilization of EL1. As discussed previously, mutation of K101^2.68^ in the EC TM2 **CRAC** motif, which also includes Y96^2.63^, abolishes receptor signaling, underscoring the crucial role of cholesterol binding at this site in maintaining the active conformation of 5-HT_1A_R.^243^

**Figure 8:**
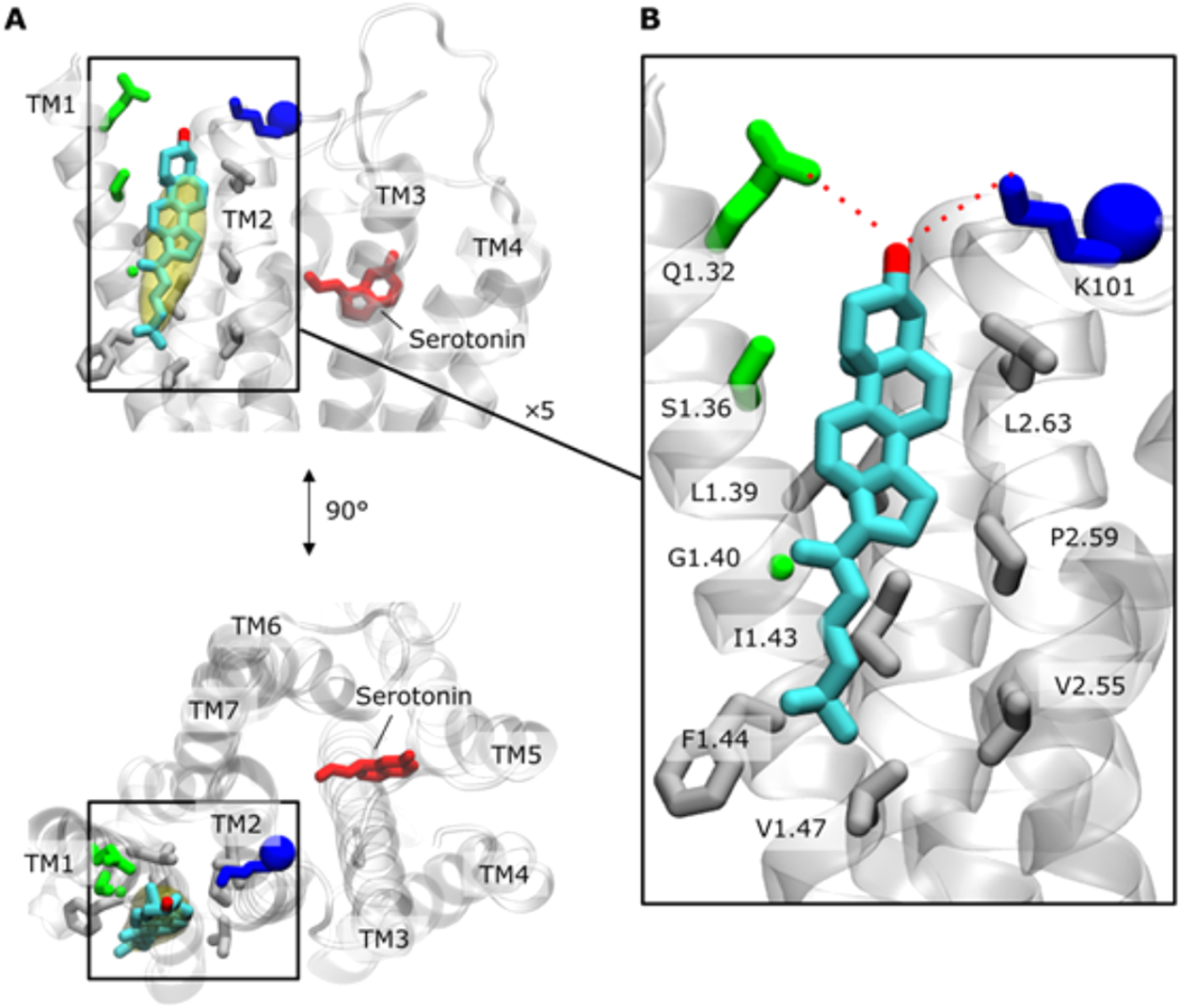
Favorable cholesterol interaction at the EC TM2 **CRAC** motif of 5-HT_1A_R, investigated via AA MD simulations.^243^ (A) The cholesterol occupancy surface (in yellow) highlights the favorable interaction region around residue K101^2.68^ of the EC TM2 **CRAC** motif. The side chain of K101^2.68^ is shown as a dark blue stick, with its Cα atom represented as a van der Waals sphere. A representative cholesterol molecule associated with this site is shown in light blue stick representation, and the orthosteric ligand serotonin is illustrated in red. (B) Residues interacting with the cholesterol molecule at the EC TM2 **CRAC** site are shown. Polar interactions between cholesterol and protein are marked with red dotted lines. Nonpolar residues are depicted in gray, and polar residues in green on the 5-HT_1A_R surface (adapted from ref 243).

The EC TM2 **CRAC** motif predicted in ref 243 using CG MD simulations and validated through functional assays, was not resolved in the cryo-EM structures reported in ref 260. Nevertheless, equilibrium CG MD simulations combined with binding saturation curves were employed in ref 55 to characterize the binding strength of the ten cholesterol-interaction sites identified in the cryo-EM structure of the apo-5-HT_1A_R–Gi (PDB ID 7E2X^269^). Remarkably, the simulations yielded good agreement between the calculated and experimental affinities of the ten cholesterol sites, revealing that the highest-affinity site—located at the EC TM1/TM7 interface—stabilizes the OBS and regulates the binding of aripiprazole.^55^ This is consistent with the cryo-EM structure of serotonin–5-HT_1A_R–Gi complex (PDB ID 7E2Y).^260^ Furthermore, the calculated affinities were found to increase with the burial depth of the cholesterol molecule, with the binding strength varying by approximately 1 *kT*.^55^

5-HT_1A_R exhibits neuronal cell-type and species specificity,^256,257^ which depends on the lipid content of neuronal membranes,^258,259^ suggesting complex lipid interaction patterns in these specialized environments. To investigate this, long CG MD simulations were performed^33^ on 5-HT_1A_R embedded in a neuronal membrane, using a homology model of 5-HT_1A_R in the intermediate-active conformation. This model was based on the experimental structure of the agonist ergotamine–5-HT_1B_R complex (PDB ID 4IAR^266^) as the cryo-EM structure of 5-HT_1A_R^260^ had been published only a few months prior to the study in ref 33. The neuronal membrane model included phospholipids, sphingolipids (including glycosphingolipids) with differing headgroups and acyl chains, and cholesterol. The simulations revealed high cholesterol occupancy at several sites on the TM helices, including EC TM1 (residues S34^1.30^, L41^1.37^, L42^1.38^, F48^1.44^) and EC TM5 (residues I197^EL2^, Y198^EL2^, F201^5.45^, F204^5.47^), consistent with earlier CG MD^152,263^ and AA MD simulations.^243^ However, cholesterol binding at the IC TM5 **CRAC** motif, predicted by bioinformatics^51,53,159^ and AA MD simulations,^243^ was not observed. Additionally, lipid–lipid crosstalk was analyzed. The simulations identified lipids that bind synergistically to 5-HT_1A_R and others that bind competitively.^33^ It was found that cholesterol, GM1, and PE interact synergistically by adopting complementary near EC TM1 and packing well together. The receptor showed a preference for interactions with unsaturated lipid tails over saturated ones, both within a lipid and between different lipids. In contrast, cholesterol and SM were observed to compete for binding at several sites, despite their coexistence in lipid nanodomains. Key residues involved in this competitive binding included IC residues I197^EL2^, Y198^EL2^, F201^5.45^, and F204^5.47^, which also form a sphingolipid-binding motif. Notably, ΤM5 of 5-HT_1A_R appears to contain both a sphingolipid-binding motif and multiple cholesterol-interaction sites at various depths of the helix, highlighting its multifunctional lipid-sensing role.^235,270^

Several replicates of long CG MD simulations were performed in ref 130 using a homology model based on the X-ray structure of agonist ergotamine-5-HT1BR complex (PDB ID 4IAR^266^), embedded in a neuronal-like membrane, to investigate the energetics and dynamics of lipid association as initially explored in ref 33. Binding free energy calculations were derived from equilibrium CG MD simulations and cholesterol density distributions. The results showed that for a cholesterol molecule in the bulk membrane to reach an annular site on the receptor, it must overcome an activation barrier of approximately 7–8 *kT*, although the associated free energy change is relatively low. Due to the high number of lipids in the bulk, multiple transitions from bulk to annular binding sites were observed. From an annular site, cholesterol may transition to an intermediate site, crossing an additional activation barrier (∼5–12 *kT*), and then to a nonannular site. Typically, cholesterol binds to the receptor’s surface, forming shallow free energy minima in the range of ∼1–3 *kT*, consistent with US/PMF results for other GPCRs such as the adenosine receptors.^131^ The intersite variability in cholesterol binding free energy reported in ref 130 was ∼1 *kT*, like the findings in ref 55. This suggests modest variations in the energetics of specific cholesterol–site interactions, while the energy barriers connecting these sites are considerably higher. The predicted nonannular cholesterol interaction site with the highest residence time (∼10 μs) was located at an energy minimum of ∼−3 *kT* at the EC TM1/TM7 interface, corresponding to the binding site also identified in the cryo-EM of active-state 5-HT_1A_R.^260^ The simulations further indicated that other cholesterol-interaction sites observed in the cryo-EM structure exhibited slower association/dissociation kinetics. These cholesterol “hot spots” are interconnected with intermediate and annular binding sites, aligning with the multiple binding time scales reported in β_2_AR using solution NMR^271^ and the multiple lipid-binding sites identified in membrane proteins via nMS.^272^

#### 2.5.5 5-HT_2A_R: Cryo-EM, X-ray Crystallography–MD Simulations

Experimental structures and MD simulations of 5-HT_2A_R complexes suggest that cholesterol-interaction site positions are dependent on the receptor’s activation state, varying between the inactive, intermediate-active, and fully activated conformation. In the cryo-EM structure of the fully activated 25CN-NBOH–5-HT_2A_R–Gαq complex (PDB ID: 6WHA^273^), one cholesterol molecule is located at the IC TM2–TM4 **CCM** and another at the EC TM6/TM7 (see Table 1).^273^

In the crystal structure of the inactive 5-HT_2A_R bound to the inverse agonist 89F (PDB ID 6WH4),^273^ one cholesterol is observed at the IC TM1/H8 interface. In the antagonist-bound inactive structure with lumateperone (PDB ID 7WC8 ^274^), two cholesterols are cocrystallized at EC TM1 and TM1/TM2, and two more at IC TM3/TM4/TM5 and IC TM6/TM7. In the intermediate-active 5-HT_2A_R structure bound to an agonist (PDB ID 7WC4 ^274^) three cholesterol molecules were cocrystallized: at IC TM1/H8, IC TM2–TM4 **CCM**, and EC TM6/TM7. If these cocrystallized cholesterols are not artifacts, their number and location appear to vary depending on whether the receptor is in an antagonist-bound inactive state, an agonist-bound preactive state, or a fully activated G protein-bound state. These variations may reflect conformational changes that modulate cholesterol accessibility and affinity.

Even among structures bound to ligands of the same type, such as agonists or antagonists, the number of cocrystallized cholesterol molecules can differ. For example, structures of 5-HT_2A_R bound to different biased agonists show varying cholesterol occupancy: three cholesterols (one EC, two IC) with serotonin (PDB ID 7WC4), two (one EC, one IC) with psilocin (PDB ID 7WC5), one EC cholesterol with LSD (PDB ID 7WC6), and one EC cholesterol with lisuride (PDB ID 7WC7) (see also Table 1 respectively^274^).

AA MD simulations^275^ of 5-HT_2A_R bound to the partial agonist LSD (based on the homology model of inactive β_2_AR with PDB ID 2RH1 ^104^), or the inverse agonist ketanserin, identified several cholesterol-interaction sites. These include IC TM2, IC TM2–TM4 **CCM**, the EC TM2/TM3, as well as both EC and IC regions of TM6/TM7. Additionally, CG MD simulations of 5-HT_1B_R,^15^ identified prominent cholesterol-interaction sites at IC TM1/H8, with additional sites at IC TM1/TM2, IC TM2–TM4 **CCM**, IC TM6/TM7, and EC TM1/TM7. These results highlight the dynamic and conformation-dependent nature of cholesterol binding across serotonergic GPCRs.

### 2.6 OXTR

#### 2.6.1 Biochemical Assays–Results from Bioinformatics Analysis–X-ray Crystallography–FRET

The activation of the OXTR has therapeutic potential for treating mental health disorders such as autism, Asperger’s syndrome, social anxiety disorder, and schizophrenia. Conversely, OXTR antagonism may be beneficial for managing male sexual dysfunction and spontaneous premature labor. However, in obstetric applications, the only clinically approved OXTR-targeted therapies are peptide-based ligands, which require intravenous administration.^97^

Cholesterol has been shown to dramatically enhance OXTR function: it increases agonist binding affinity by approximately 100-fold in a dose-dependent manner, enhances antagonist affinity, and improves receptor stability.^276–278^

The X-ray structure of the nonpeptidic antagonist retosiban–OXTR complex (PDB ID 6TPK^97^) solved from a monoolein: cholesterol mixture revealed a cocrystallized cholesterol molecule bound at the EC TM4/TM5 **CCM**. This site differs from the IC TM2–TM4 **CCM** predicted for OXTR via bioinformatics analysis^51,53,159^ (see also Table S1). The cholesterol binds primarily through hydrophobic interactions with residues Y200^5.38^ and W203^5.41^, as well as P170^4.59^, I174^4.63^, V190^EL2^, F191^EL2^, W295^EL2^, G196^5.43^, and A199^5.37^.^97^ A hydrogen bond also forms between the hydroxyl group of cholesterol and the I192^EL2^. Interestingly, mutations at Y200^5.38^A and W203^5.41^A significantly affect receptor thermostability in the presence of CHS, indicating disruption of the cholesterol-binding site. These findings, together with the proximity of the OBS to the EC TM4/TM5 **CCM**, suggest that cholesterol plays a critical role in maintaining the structural integrity of the OBS and optimal ligand positioning.^97^

Further evidence came from the development of ion channel-coupled receptor (ICCR) technology, which enables cholesterol modulation to be functionally assessed in live cells, independently of intracellular signaling pathways.^279^ This study showed that ligand binding stabilizes cholesterol interaction at the EC TM4/TM5 **CCM** and confirmed that cholesterol is essential for high-affinity ligand binding and signaling. Time-resolved FRET assays using fluorescently labeled OXTR expressed in CHO cells supported these findings. Moreover, the data suggest that ligand binding may promote OXTR dimerization, with one or more cholesterol molecules stabilizing the dimer interface.^279^

### 2.7 Cholecystokinin Receptors 1 and 2

#### 2.7.1 Biochemical Assays–Results from Bioinformatics Analysis–Cryo-EM–MD Simulations

Cholecystokinin receptors (CCKRs) 1 and 2 are highly homologous and both bind and are activated by the endogenous peptide CCK-8, signaling predominantly through Gq/11 pathways to reduce intracellular calcium levels. However, CCK_1_R displays a distinct functional sensitivity to membrane cholesterol compared to CCK_2_R, prompting in-depth studies using biochemical, structural biology, and MD simulations approaches. Bioinformatics analyses^51,53,159^ (see also Table S1) identified a conserved IC TM4 **CCM** and two IC TM7/H8 and IC TM3 **CRAC** motifs across class A GPCRs.

Multiple cholesterol molecules are observed in cryo-EM structures of both CCK_1_R and CCK_2_R in complex with CCK-8, where the ligand adopts a similarly deep binding pose—for instance, in the CCK8–CCK_1_R–mGqsi complex (PDB ID 7MBY^280^), the CCK8 analog–CCK_1_R–Gq complex (PDB ID 7EZM^281^) and the gastrin-17–CCK_2_R–Gq complex (PDB ID 7F8W^282^), as shown in Figure 9.

**Figure 9:**
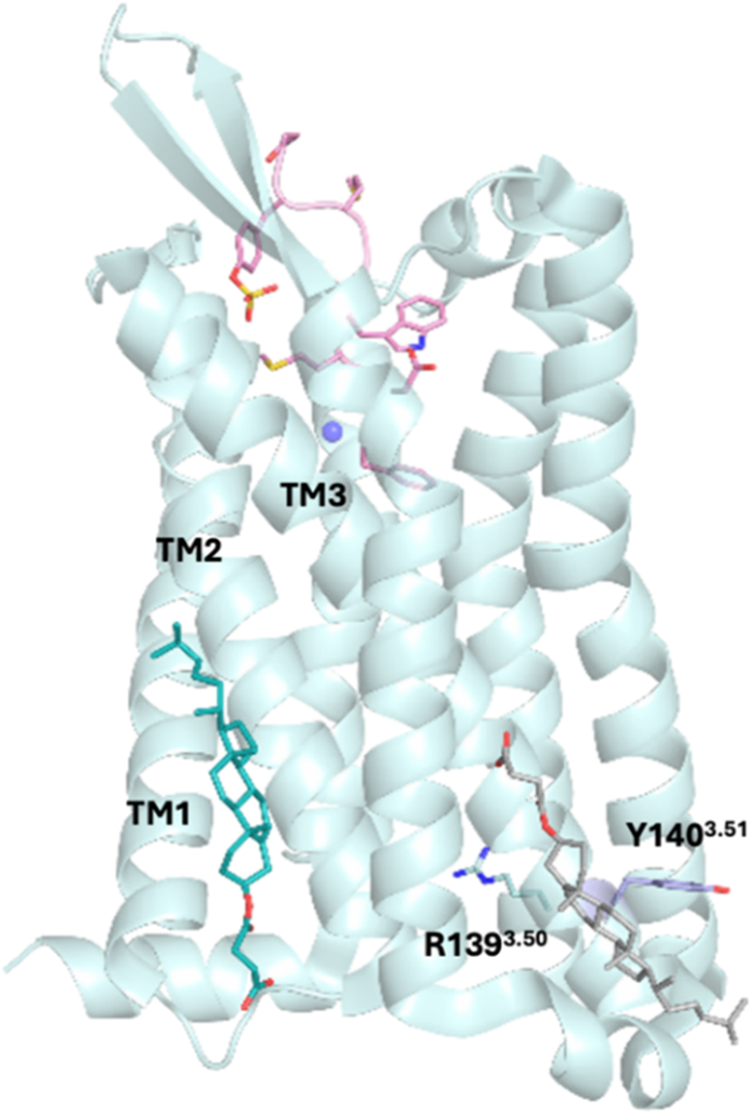
Cryo-EM structure of the CCK-8–CCK_1_R–Gi protein complex (PDB ID 7MBY^280^), with the Gi protein omitted for clarity. One cholesterol molecule located at the IC TM1/TM2 interface is shown in light blue stick representation. The orthosteric agonist CCK-8 is depicted in pink sticks, with nitrogen in blue, oxygen in red, and sulfur in yellow. A second cholesterol molecule, illustrated in gray sticks, lies in the cleft formed by an intracellular TM3 CRAC motif, and is suggested to enhance CCK-8 binding and Gq protein coupling. However, in the cryo-EM maps, only weak densities—potentially corresponding to CHS—are observed near Y140^3.51^, with the density extending toward the IL2 loop.

However, only CCK_1_R exhibits functional sensitivity to cholesterol, which acts as a PAM for CCK-8, enhancing agonist affinity and reducing intracellular calcium signaling.^116,283^ Studies^116,283^ suggested that the key allosteric cholesterol-binding site is the cytoplasmic IC TM3 **CRAC** motif in CCK_1_R with residue Y140^3.51^ playing a pivotal role. Mutation of Y140^3.51^ to alanine disrupts this motif, abolishing cholesterol sensitivity.^111^ Although CCK_2_R shares similar **CRAC** motifs in the IC TM3 region, it remains insensitive to cholesterol, making this receptor pair a valuable model for studying cholesterol-mediated modulation. While cryo-EM structures revealed only weak densities—possibly CHS—around the IC TM3 and IL2 regions, this suggests potential modulation of conformational dynamics by cholesterol.^116,117^

CG MD simulations on CCK_1_R and CCK_2_R,^183^ despite their sequence similarity, they revealed distinct cholesterol-interaction profiles. In CCK_1_R, cholesterol interacts with F130^3.41^, in a **CRAC** motif at IC TM3, whereas in CCK_2_R, cholesterol binds predominantly to a **CCM** near S95^2.45^ at IC TM2. Furthermore, CCK_1_R showed more specific and longer-lasting cholesterol binding than CCK_2_R, potentially explaining their differing physiological responses to high-cholesterol environments.

To validate these findings, radioligand binding, intracellular calcium measurements, and BRET-based G protein conformational assays were performed.^114^ Additionally, fluorescence polarization kinetic assays using fluorescently labeled CCK-8 analogs assessed agonist dissociation rates. Structural comparison of CCK_1_R Y140^3.51^A (PDB ID 9BKJ^114^), CCK_1_R­(sterol 7M) (PDB ID 9BKK^114^), and wild-type CCK_1_R showed minimal structural deviation (RMSD (Cα) ∼ 0.7 Å), despite altered cholesterol sensitivity. Notably, seven residues within 8 Å of Y140^3.51^ differ between CCK_1_R and CCK_2_R: F130^3.41^/L143^3.41^, S136^3.47^/A149^3.47^, G141^3.52^/S154^3.52^, I216^5.46^/L225^5.46^, L219^5.48^/F228^5.48^, I223^5.52^/V232^5.52^, M226^5.55^/A235^5.55^. Swapping these residues produced the CCK_1_R­(sterol 7M) and CCK_2_R­(sterol 7M) variants, effectively reversing cholesterol sensitivity. While CCK_1_R­(sterol 7M) lost cholesterol responsiveness but retained typical binding and calcium signaling, CCK_2_R­(sterol 7M) became cholesterol-sensitive, exhibiting enhanced agonist affinity and diminished calcium mobilization.

Photoaffinity cross-linking with CCK-8 analogs modified at F33 supported distinct ligand engagement modes for CCK_1_R and CCK_2_R, with binding at W39^1.30^ in CCK_1_R and T119^EL1^ in CCK_2_R. These results imply that CCK-8 binds CCK_1_R with dynamic flexibility, transitioning between shallow and deep poses, facilitating faster Gq/11 coupling and more efficient calcium signaling. In contrast, CCK_2_R forms a more stable CCK-8 complex with slower G protein engagement and reduced signaling potency. The IC TM3 **CRAC** motif’s proximity to IL2—a key G protein interface—suggests a mechanistic basis for cholesterol’s influence on signaling. Elevated membrane cholesterol enhances **CRAC**–cholesterol interactions in CCK_1_R, stabilizing Gq/11 coupling and increasing CCK-8 affinity while decreasing its dissociation rate (*k*_off_), thus lowering intracellular calcium levels. In contrast, the slower G protein activation and ligand *k*_off_ in CCK_2_R underlie its insensitivity to elevated cholesterol.

### 2.8 P2Y1R

#### 2.8.1 Biochemical Assays–Results from Bioinformatics Analysis–X-ray Crystallography–MD Simulations

While the specific impact of cholesterol on P2Y1R function remains unclear, experimental studies have shown that cholesterol depletion does not affect P2Y1R-mediated calcium release in platelets.^284^ Bioinformatics analysis (see Table S1) identified a conserved IC TM4 **CCM** in P2Y1R.

The X-ray structure of P2Y1R in complex with the non-nucleotide antagonist BPTU—an antithrombotic agent—(PDB ID 4XNV^284^), revealed that BPTU binds to an allosteric pocket located at the interface of TM1–TM3, EL1, and the lipid bilayer. This structure was notable as it was the first to capture a selective ligand binding entirely outside the helical bundle of a GPCR. In this complex, three CHS molecules cocrystallized at EC sites TM1/TM7, TM4/TM5, and an IC site involving TM3/TM4/TM5 (see Table 1). In contrast, the X-ray structure of P2Y1R in complex with the nucleotide antagonist MRS2500, which binds within the canonical orthosteric site inside the 7TM bundle (PDB ID 4XNW^284^) showed no cocrystallized CHS or cholesterol molecules.

Long-time-scale CG MD simulations^15^ confirmed cholesterol binding at the extracellular TM4/TM5 interface, consistent with the experimental structure of the BPTU–P2Y1R complex.^284^ These findings support the notion of specific cholesterol interaction sites modulating the receptor, particularly in the context of allosteric ligand binding.

### 2.9 Cannabinoid Receptors 1 and 2

#### 2.9.1 Biochemical Assays–Fluorescence Microscopy–Results from Bioinformatics Analysis

Experimental studies have demonstrated that disrupting lipid rafts with MβCD enhances CB_1_R signaling, while CB_2_R signaling remains unaffected. This differential modulation by cholesterol depletion has sparked interest in uncovering the molecular basis behind the distinct responses of these cannabinoid receptors.

Specifically, treatment with MβCD was shown to double CB_1_R agonist binding and signaling in rat C6 glioma cells.^285^ Similarly, experiments on human immune cell membranes revealed that MβCD treatment increases CB_1_R signaling activity, while having no significant effect on CB_2_R activation^286,287^ or the binding of the agonist CP-55,940 on CB_2_R.^286^ Interestingly, cholesterol has been identified as a positive allosteric modulator (PAM) of CB_2_R, enhancing its constitutive activity.^101^ This effect was observed in membrane preparations from mammalian (HEK, CHO), insect, and bacterial cells expressing CB_2_R. To eliminate variables associated with native membrane composition, CB_2_R was reconstituted into synthetic lipid bilayers composed of POPC/POPG (3:1) with varying cholesterol concentrations of 0–40%.^101^ The presence of cholesterol in these model systems significantly enhanced CB_2_R activation and signaling. Moreover, altered ligand pharmacology: a compound classified as a partial agonist in cholesterol-free membranes acted as a neutral antagonist or partial inverse agonist in cholesterol-rich membranes.^101^

Sequence alignment studies^51,53,159^ identified conserved IC cholesterol-interaction motifs in both CB_1_R and CB_2_R, including an IC TM4 **CCM**, and a **CRAC** motif at IC TM7/H8 in CB_1_R and at IC TM7 in CB_2_R (see also Table S1). Sequence analysis revealed a 44% identity between CB_1_R and CB_2_R across the entire protein, with high conservation (82%) in the IC TM7 **CRAC** region.^51,53^ Notably, the **CRAC** motif in this region includes V392^7.48^, Y397^7.53^, and K402^8.48^ in CB_1_R, compared to V294^7.48^, Y299^7.53^, and G304^8.48^ in CB_2_R. The only difference is at position 8.48, where CB_1_R has a lysine and CB_2_R a glycine (Figure 10A).

**Figure 10:**
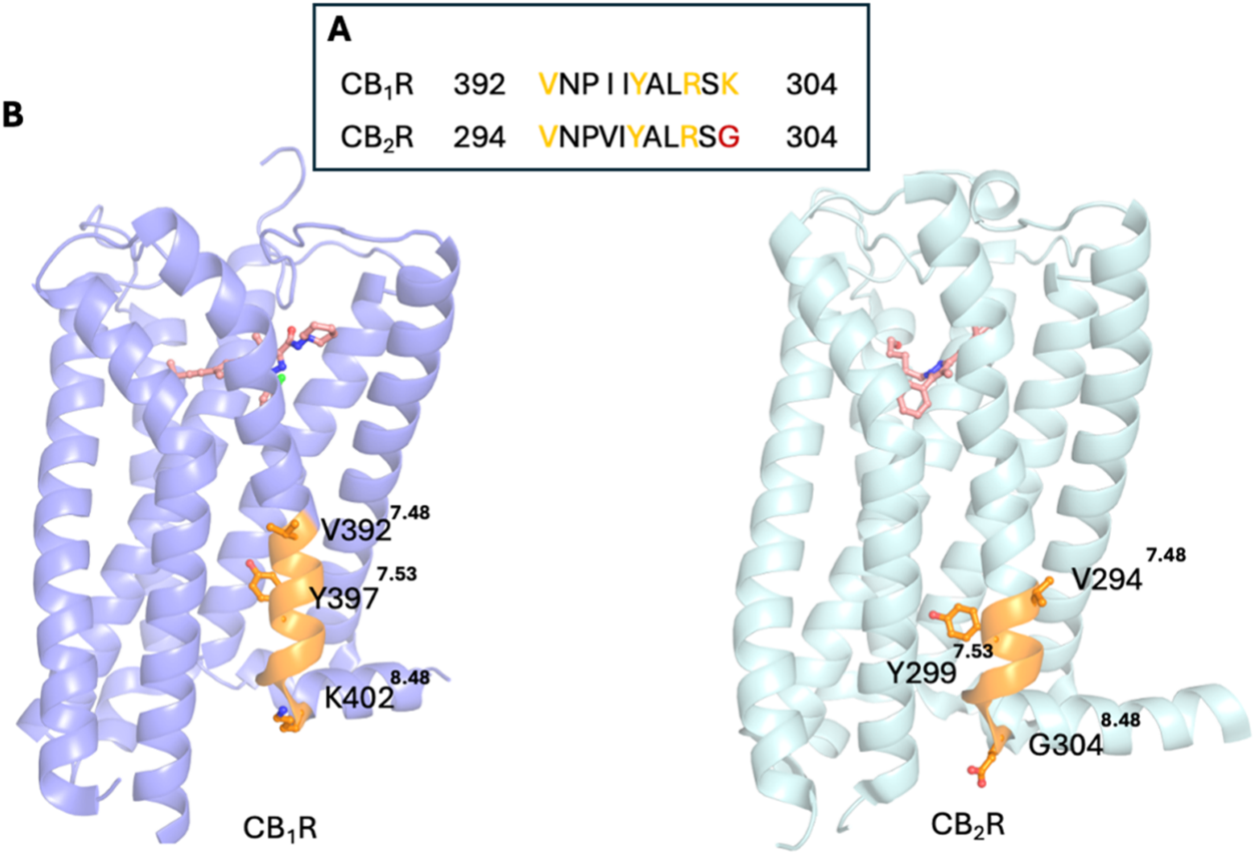
(A) Sequence alignment of CB_1_R and CB_2_R at the C-terminal end of TM7. Residues comprising the IC TM7 **CRAC** motif are highlighted in yellow. A key glycine residue, absent in CB_1_R but present in CB_2_R, is marked in red. (B) Structural comparison of inactive cannabinoid receptors: Left, X-ray structure of CB1R in complex with the stabilizing antagonist AM6538 (PDB ID 5TGZ^288^). Right, X-ray structure of CB_2_R in complex with the antagonist AM10257 (PDB ID 5ZTY^289^). The IC TM7 **CRAC** motif regions are highlighted in orange, with the side chains of key residues shown as sticks. Residue 8.48, which contributes to cholesterol stabilization, is also indicated.

In ref 290 the importance of this single-residue difference was examined by site-directed mutagenesis. A CB_1_R mutant (K402^8.48^G) was created, effectively converting its IC TM7 **CRAC** motif into that of CB_2_R. FRAP experiments in living cells revealed that the K402^8.48^G mutation reduced the receptor’s association with cholesterol-rich membrane microdomains and diminished its functional response to elevated cholesterol levels. These findings suggest that the IC TM7 **CRAC** motif in CB_1_R facilitates preferential localization to cholesterol-rich domains and contributes to cholesterol-dependent modulation of receptor activity.^290^

#### 2.9.2 CB_2_R: X-ray and Cryo-EM–MD Simulations

In the cryo-EM structure of active CB_2_R in complex with the agonist WIN 55,212-2 and Gi protein (PDB ID 6PT0 ^291^), one cholesterol molecule was observed at the IC TM4 region, and two additional cholesterol molecules were located along the EC TM7 (see Table 1). In contrast, no cholesterol molecules were observed in the X-ray structure of inactive CB_2_R bound to the antagonist AM10257 (PDB ID 5ZTY^289^). Although bioinformatics analysis (see Table S1) identified a conserved **CRAC** motif at IC TM7 (as illustrated in Figure 10B) and biochemical data have highlighted its functional importance, there is currently no structural evidence of favorable cholesterol binding at this site in the available CB_2_R experimental structures.

AA MD simulations of apo-CB_2_R embedded in cholesterol-containing membranes revealed significant conformational deviations in the IL3-TM6 region, which is implicated in Gα protein coupling, particularly in the antagonist-bound conformation. In contrast, CB_2_R bound to the antagonist MRI-2659 exhibited minimal structure deviation in this region, consistent with the strong antagonist activity of MRI-2659 irrespective of cholesterol presence. However, the potential role of the IC TM7 **CRAC** motif in modulating the conformation of the IL3-TM6 region remains to be elucidated.

#### 2.9.3 CB_1_R: X-ray Crystallography and Cryo-EM–NMR Spectroscopy–MD Simulations

The first experimental structures of CB_1_R with cocrystallized cholesterol include complexes with the agonist only AM841 (PDB ID 5XR8 ^292^) and AM11542 (PDB ID 5XRA^292^). In both agonist-bound structures, cholesterol molecules from the LCP crystallization medium associate with the indole side chain of the conserved residue W241^4.50^ and R145^IL1^ in IC TM2–TM4 **CCM**, also seen in other class A GPCRs, such as β_2_AR (PDB IDs 2RH1,^104^ 3D4S,^113^ see Section 2.3.4 and Table 1). A similar cholesterol-binding pattern is seen in the FUB–CB_1_R–Gi structure (PDB ID 6N4B^293^), where two cholesterol molecules are located at the IC TM4/TM5 interface (see Table 1). In the X-ray structure of an intermediate-active state of CB_1_R with the agonist CP55940 and the PAM ZCZ011 (PDB ID 7FEE^294^), two cholesterol molecules are observed: one in the IC TM2–TM4 **CCM** and another along TM4 (Figure 11B). Likewise, a cholesterol molecule cocrystallized at IC TM2–TM4 **CCM** in the X-ray structure of agonist CP55940-CB_1_R complex (PDB ID 7V3Z,^295^ see Figure 11C). Interestingly, in the presence of NAM ORG27569, which binds at IC TM2–TM4 **CCM**, no cholesterol is observed (PDB ID 6KQI,^296^ see Figure 11A). This suggests that cholesterol and ORG27569 may compete for overlapping binding regions. In both cases, these modulators bind at peripheral sites, within the membrane-receptor interface.

**Figure 11:**
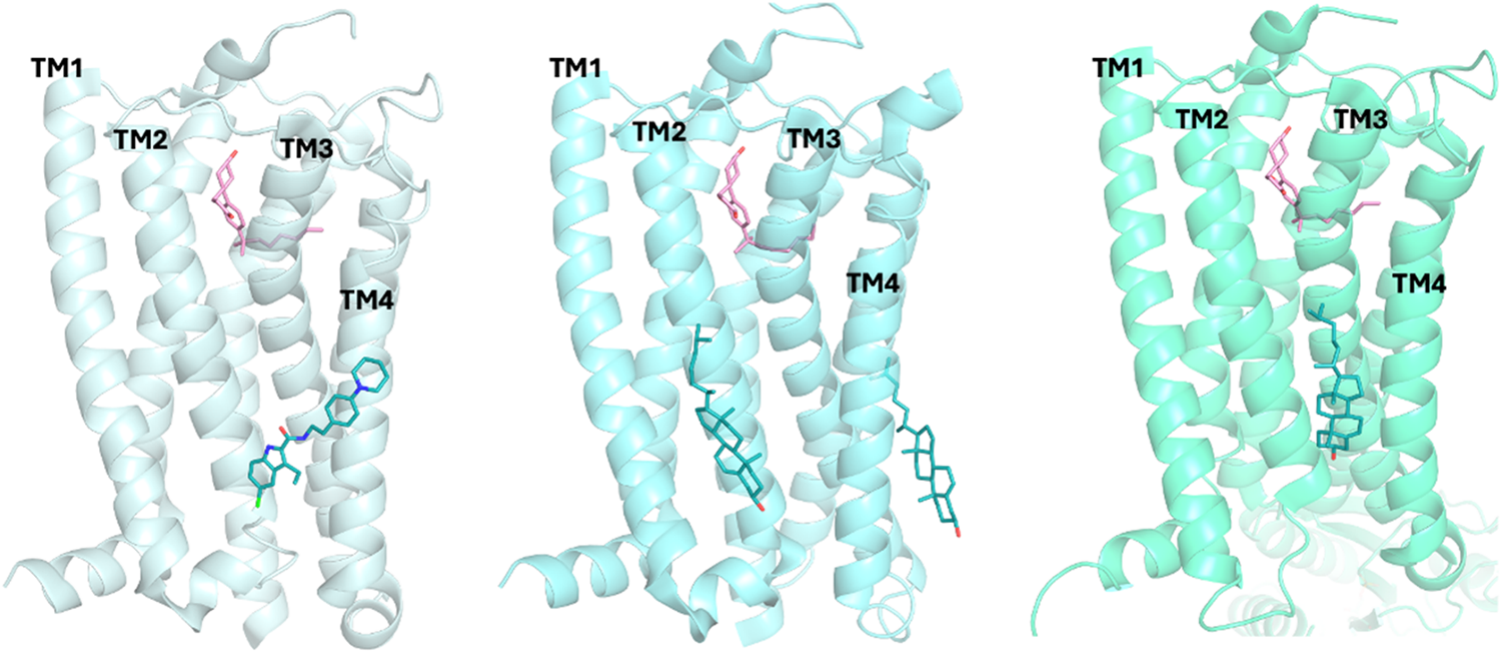
Structural comparison of CB_1_R in complex with CP55940 and various allosteric modulators or cholesterol, each adopting an intermediate-active conformation. (A) CP55940–NAM ORG27569–CB1R complex (PDB ID 6KQI).^296^ (B) CP55940–PAM ZCZ011–CB_1_R complex (PDB ID 7FEE),^294^ featuring two cholesterols associated with the IC TM2–TM4 CCM. (C) CP55940–CB1R complex (PDB ID 7V3Z^295^), featuring one cholesterol at the IC TM2–TM4 **CCM**. In all panels, CB_1_R is shown as a ribbon representation (light blue or light green), the orthosteric ligand CP55940 is shown in pink sticks, and cholesterol, PAM, or NAM ligands are represented in light blue sticks. Nitrogen atoms are colored blue, and oxygen atoms are red.

In contrast, the antagonist-bound CB_1_R structure (PDB ID 5TGZ^288^) shows no cholesterol binding. Similarly, in the cryo-EM structure of the fully active CB_1_R bound to CP55940, Gi protein, and PAM ZCZ011 (PDB ID 7WV9)^294^, no cholesterol is observed, either at EC TM2–TM4 (where ZCZ011 binds) or at the intracellular Gi-coupling interface. These findings support the hypothesis that cholesterol at the IC TM2–TM4 **CCM** may function as a NAM, analogous to ORG27569. Notably, no cholesterol molecules have been observed in the predicted **CRAC** motifs of TM7 in either CB_1_R or CB_2_R.^290^ This absence may relate to cholesterol’s role in targeting CB_1_R to cholesterol-rich membrane domains.

To further investigate, ^19^F NMR spectroscopy was performed on CB_1_R reconstituted in LMNG/CHS micelles and bound to CP55940 with or without ORG27569.^295^ CB_1_R labeled at the cytoplasmic ends of TM6 and TM7 revealed conformational populations consistent with basal activity: ∼45% and <5% active-like states, respectively. Agonist binding shifted these populations to 55–70% and 30–95%, respectively. Both cholesterol and ORG27569 modulate these equilibria by increasing the population of preactive and active-like conformations. This aligns with X-ray and cryo-EM evidence that the intracellular cavity of CB1R is more closed in inactive states (e.g., taranabant-bound, PDB ID 5U09 ^297^), more open in intermediate-active states (e.g., CP55940–ORG27569–CB_1_R with PDB ID 6KQ^296^), and fully accessible in active conformations (e.g., CP55940–Gi–ZCZ011–CB_1_R with PDB ID 7WV9;^294^ AM841–Gi–CB_1_R with PDB ID 6KPG^298^).

Long CG MD simulations^15^ revealed additional cholesterol interaction sites at IC TM1/TM7, EC TM1/TM2, IC TM1/TM2, IC TM3/TM4/TM5, and IC TM6/TM7, in agreement with observed structural data. Notably, IC TM2–TM4 **CCM**, while present in crystal structures, exhibited shorter residence time in simulations. AA MD simulations using the AM841–CB_1_R structure as a starting model (PDB ID 5XR8 ^292^) showed cholesterol stably interacting with IC TM2–TM4 **CCM**, EC TM3–5 (around T274^5.38^), EC TM1 (S114^1.30^), and IC TM4. The first three sites demonstrated high cholesterol residence time, while the IC TM4 site was more transient. Interestingly, this IC TM4 region also corresponds to a high-affinity cholesterol-binding site in CB_2_R (PDB ID 6PT0 ^291^), suggesting a shared mechanism across cannabinoid receptors.

### 2.10 D_1_R

#### 2.10.1 Biochemical Assays–FRAP/FRET–Results from Bioinformatics Analysis–Cryo-EM

Functional assays and fluorescence microscopy have underscored the crucial role of lipid rafts in effective D_1_R signaling, with bioinformatic analyses identifying structural motifs that may anchor D_1_R in these membrane microdomains.^299–301^ A **CRAC** motif in the extracellular TM5 region (residues 212^5.51^-220^5.59^ sequence: **V**T**Y**T**R**I**YR**I) has been predicted (Table S1).^301,302^ In one study, cAMP-based functional assays, sodium transport measurements, and confocal microscopy were used to assess D_1_R localization in membrane microdomains, complemented by in vivo experiments in mice.^301,303^ These results revealed that D_1_R signaling requires localization within lipid rafts. Structural features, such as palmitoylation at C347^8.60^ and the **CRAC** motif containing Y218^5.62^, appear essential for maintaining raft residency. Mutations at either site result in receptor exclusion from lipid rafts, diminished agonist (fenoldopam)-stimulated cAMP production, impaired sodium transport, and increased oxidative stress in renal proximal tubule cells.^303^ In C57BL/6J mice, MβCD-induced cholesterol depletion displaced D_1_R from the brush border, reduced sodium excretion, and elevated both oxidative stress and blood pressure.^301^ Furthermore, silencing the renal Drd1 gene increased blood pressure, which was reversed only by reintroducing wild-type D_1_R—not the C347A mutant lacking the palmitoylation site.^301^ Earlier studies showed that reducing the cholesterol/phospholipid ratio in synaptic membranes by 30% decreased the binding affinity of the D_1_R agonist SCH23390, suggesting cholesterol acts as a PAM for D_1_R^301^ While biochemical assays have demonstrated the functional importance of the TM5 **CRAC** motif,^301^ this motif has yet to be resolved in experimental D_1_R structures.

FRAP and FRET-FLIM microscopy have been used to explore the lateral mobility and membrane localization of D_1_R.^301^ Cholesterol depletion via MβCD disrupted cholesterol-rich regions, causing the redistribution of D_1_R and Gαi3 within the plasma membrane. Gβ1γ2 dimers and dopamine-bound D_1_R influenced the membrane distribution of Gα subunits. Gαs, which promotes cAMP production via D_1_R activation, is localized preferentially to raft-like regions, in contrast to Gαi.^301^ These findings reinforce the importance of lipid rafts in D1R signaling.^297,301,303–310^

Cryo-EM structures of D_1_R–Gs complexes consistently show cholesterol binding at an intracellular TM2–TM4 **CCM**, implying a role in Gs stabilization or as a PAM. Notably, ∼7% of all cholesterol-containing GPCR structures are D_1_R complexes.^301^ D_1_R also binds allosteric modulators peripherally, at the receptor–membrane interface. In three D_1_R–mini-Gs cryo-EM structures bound to fenoldopam (PDB ID 7X2C^301^), tavapadon (PDB ID 7X2D^301^), or dopamine with PAM LY3154207 (PDB ID 7X2F^301^), a cholesterol molecule consistently binds within the IC TM2–TM4 CCM. In the latter (7X2F), an additional cholesterol is found near TM4, interacting with PAM LY3154207, which binds at the IC TM4/TM5 interface. Notably, the electron density for this cholesterol could also be attributed to a second LY3154207 molecule, given their similar shapes and the resolution.^301^ In other cryo-EM structures of the D_1_R–Gsαβγ complex bound to LY3154207 (PDB IDs 7CKW, 7CKZ^301^), a cholesterol consistently binds in the IC TM2–TM4 **CCM**.^301^ In PDB ID 7X2F,^301^ LY3154207 occupies a pocket above IL2, between TM4 and TM5. IL2 adopts an α-helical conformation to effectively engage Gs. The positioning of LY3154207 mirrors the binding mode of the β_2_AR PAM Cmpd-6FA.^301^ Cholesterol at TM4 further stabilizes LY3154207 binding. The PAM also appears to induce cholesterol association at its binding site, forming a PAM–cholesterol–receptor triad.^301^ Mutations at residues involved in LY3154207 binding—W123^3.52^A, R130^IL2^A, and A139^4.45^L—or possibly affecting the nearby cholesterol-binding site at TM4, reduce or abolish LY3154207’s allosteric effects without significantly impacting orthosteric ligand binding.^301^

Interestingly, other D_1_R–mini-Gs cryo-EM structures show cholesterols at varied locations. In the SKF81297–LY3154207–D_1_R–mini-Gs complex (PDB ID 7LJC^304^), cholesterols associate with IC TM5, IC TM6, EC TM5 (two molecules), and EC TM6. In the dopamine–LY3154207–D_1_R–mini-Gs complex (PDB ID 7LJD^304^), cholesterols bind at IC TM4 and IC TM6, as well as along EC TM1, EC TM4, and EC TM5 (see Table 1).

### 2.11 NTS1R

#### 2.11.1 Results from Bioinformatics Analysis–Biochemical Assays–FRET–EPR–MD Simulations

Interestingly, while the bioinformatics analysis identified the presence of cholesterol-interaction motifs,^47^ the comparison of functional assays or MD simulations demonstrates contradictory results as regards the effect of cholesterol on NTS1R signaling^47,305^ or on the receptor’s homo-oligomerization, respectively. Sequencing analysis studies have identified an IC TM4 **CCM** and an IC TM7/H8 **CRAC** motif (see Table S1).^47^

It has been proposed that cholesterol regulates GPCR signaling by facilitating receptor localization within specific membrane microdomains, such as lipid rafts or caveolae—well-documented in receptors like β_2_AR.^177^ A similar spatial regulation has been observed for NTS1R, particularly in cancer cells where the receptor is overexpressed.^305^ In such systems, palmitoylation of NTS1R has been shown to be essential for mitogenic signaling, which occurs within structured membrane microdomains.^297^ Cholesterol depletion via MβCD abolishes NTS1R-mediated MAPK activation, while cholesterol repletion restores signaling, reinforcing the idea of cholesterol’s critical role in NTS1R function in native contexts.^305^ However, contrasting observations emerge in artificial systems. NTS1R is capable of ligand binding in *E. coli* membranes, which lack cholesterol^306^ and couple to G proteins in cholesterol-free conditions.^307^ A subsequent study found that cholesterol depletion did not impair NTS1R ligand binding.^47^ Moreover, FRET studies using eCFP- and eYFP-tagged NTS1R revealed that cholesterol can modulate receptor dimerization in model bilayers composed of POPC and POPE.^47^ Further complexity is introduced by studies using biophysical probes. One investigation using spin-labeled cholestane and EPR spectroscopy in NTS1R-reconstituted phosphatidylcholine proteoliposomes suggested proximity between cholesterol and the receptor.^308^ However, AA MD simulations and EPR analysis in that same study did not support direct interaction of cholesterol with the predicted **CCM** or **CRAC** motifs.^47^ In contrast, CG MD simulations in a later study showed that cholesterol does modulate both homodimerization and heterodimerization of NTS1R, supporting a role for cholesterol in receptor oligomerization.^307^ Taken together, these findings suggest that the functional relevance of cholesterol to NTS1R may be context-dependent, critically influencing receptor signaling and localization in native, cholesterol-rich membranes, while appearing dispensable for basic ligand binding and G protein coupling in reconstituted or cholesterol-poor systems. This dichotomy underscores the importance of using physiologically relevant membrane compositions when investigating GPCR function.

### 2.12 CX3CR1

#### 2.12.1 Results from Bioinformatics Analysis–Cryo-EM–Biochemical Assays

The combination of structural biology and biochemical assays revealed cholesterol-interaction sites that may regulate signaling of CX3CR1, a chemokine receptor of significant therapeutic relevance. Chemokines are a class of small signaling molecules that activate immune cells through binding to chemokine receptors (CKRs). These interactions are often promiscuous: a single chemokine can activate multiple CKRs, and individual receptors can respond to multiple chemokines. Notably, CX3CR1 is the only receptor known for the endogenous chemokine CX3CL1 (also known as fractalkine). Activation of CX3CR1 by CX3CL1, through coupling with Gi protein, plays a critical role in monocyte–endothelial cell communication in the CNS. CX3CR1 mediates several signaling pathways involved in cell migration, apoptosis resistance, and angiogenesis. Due to its involvement in diverse physiological and pathological processes, including cancer, atherosclerosis, rheumatoid arthritis, and neurodegenerative diseases, CX3CR1 is an attractive therapeutic target.^309^ Moreover, US28, a human cytomegalovirus chemokine (HCMV)-encoded GPCR with 29% sequence identity to CX3CR1, also binds to CX3CL1 and plays a role in viral pathogenesis, making it a promising antiviral drug target.^310^ Despite the pharmacological relevance of chemokine receptors, drug development remains challenging due to ligand–receptor promiscuity. To date, only three CKR-targeting drugs—Maraviroc, Plerixafor, and Mogamulizumab—have received clinical approval.^311^

Although cholesterol molecules have been resolved in cryo-EM structures of CX3CR1, their functional impact on CKRs remains largely unexplored.^312^ Bioinformatics analysis^51,53,159^ (see Table S1) identified a conserved IC TM2–TM4 **CCM** and an EC TM2 **CRAC** motif. In the cryo-EM structure of the active CX3CL1–CX3CR1–Giαβγ complex (PDB ID 7XBX),^312^ the cytoplasmic end of TM6 exhibits a minimal outward displacement- a hallmark of class A GPCR activation—compared to what is typically seen in activated GPCR–G protein complexes such as CCR5. Consistently, several conserved microswitches—including the D^3.49^R^3.50^Y^3.51^, P^5.50^I^3.40^F^6.44^, C^6.47^W^6.48^xP^6.50^, N^7.49^P^7.50^xxY^7.53^ retain inactive-like rotameric states, suggesting a distinct activation mechanism. For example, while the highly conserved “toggle switch” W^6.48^ residue and the PIF motif show conformational changes during activation of CCR5, in the case of activated CX3CR1, these motifs have the rotamers of the inactive CCR5. This conformational state may be stabilized by cholesterol, as evidenced by the presence of two cholesterol molecules in the active-state complex (PDB ID 7XBX^312^) and three in the preactive apo–CX3CR1–Giαβγ structure (PDB ID 7XBW^312^). These cholesterol molecules likely play key roles in maintaining this unique active-like conformation. The first of these three cholesterol-interaction sites corresponds to the common IC TM2–TM4 **CCM** (see Table 1) observed only in apo-CX3CR1–Gi complex (PDB ID 7XBW^312^) and in many other class A GPCRs. As has been discussed on this **CCM** site, W154^4.50^ has a universal, critical role among class A GPCRs in cholesterol stabilizing CH-π stacking interaction, and its mutation to alanine results in the inability of the receptor to couple with Gi. The other two cholesterol interaction sites were observed in both cryo-EM structures. The second cholesterol-interaction site lies at IC TM3/TM4/TM5 (see Table 1) with F118^3.41^, V153^4.49^ playing a critical role in stabilization of cholesterol and receptor since their mutation to alanine abolished signaling. In the third interaction site, cholesterol lies at IC TM5/TM6, sticking between I215^5.62^ and L234^6.38^, which restricts the outward shift of TM6; the mutation to alanine L234^6.38^A reduces signaling significantly, while in case of I215^5.62^, only I215^5.62^F is detrimental for signaling.

### 2.13 Specificity of Cholesterol Association Compared to Other Sterols

While cholesterol modulates the function of GPCRs, the extent to which specific cholesterol-receptor interactions, both in terms of receptor residues and cholesterol structure, affect GPCR function remains an area requiring further investigation. To address this, several studies have examined the effects of structurally related sterols as analogs or substitutes for cholesterol.

For example, in studies with RhoR reconstituted in lipid nanodiscs, FRET quenching between RhoR tryptophans and the fluorescent cholesterol analog cholestatrienol was inhibited by native cholesterol, indicating direct and competitive binding. However, ergosterol, a structurally similar yeast sterol, failed to inhibit this interaction, implying that the cholesterol-binding site on RhoR has structural specificity selective for cholesterol.^165^

Similarly, NMR spectroscopy experiments with β_2_AR demonstrated that both low- and high-affinity cholesterol interaction sites are specific to cholesterol and not accessible to ergosterol.^194^

In A_2A_R studies, ^19^F solution NMR and GTP turnover assays showed that cholesterol acts as a PAM.^206^ To assess whether this effect is specific to cholesterol’s structure, sterol analogs such as epi-cholesterol (an enantiomer**)**, ergosterol (a diastereomer), and digoxigenin were used. Interestingly, while all three analogs produced NMR spectra like that of cholesterol (showing conformational states **A1** ^**TM6**^ and **A2** ^**TM6**^), they differed in the relative population of these states. This suggests that sterol presence generally shifts the conformational ensemble toward activation, but the exact sterol modulates conformational bias, rather than acting through a single rigid cholesterol-specific site.

In the case of 5-HΤ_1A_R depletion/repletion experiments demonstrated cholesterol’s essential role in supporting ligand binding.^250^ To explore structural specificity, sterol substitution studies showed that ent-cholesterol (enantiomer of cholesterol) could restore receptor activity, while epi-cholesterol (diastereomer) could not, indicating that the receptor’s requirement is diastereospecific, but not enantiospecific.^250^ Additionally, substitution with 7-dehydrocholesterol or desmosterol, two immediate biosynthetic precursors of cholesterol, showed that 7-dehydrocholesterol abolished ligand binding, while desmosterol partially restored it, suggesting sensitivity to subtle structural differences in the sterol backbone.^251^ MD simulations further supported the stereospecific recognition of cholesterol by 5-HT_1B_R and 5-HT_2B_R, exhibiting distinct binding behavior for natural cholesterol versus its ent- and epi-isomers, which indicates discrimination at the level of stereochemistry.^313^

In OXTR studies, cholesterol was shown to stabilize the receptor, with high efficiency matched only by a few analogs such as cholesterol-5α-6α-epoxide and 19-hydroxycholesterol.^276^ In contrast, other sterols—including, e.g., β-sitosterol, lanosterol, progesterone, and 25-hydroxycholesterol—were either significantly less effective or completely inactive in stabilizing OXTR, demonstrating a high degree of structural selectivity for cholesterol.

These findings collectively underscore that while GPCRs often respond to membrane cholesterol, the effects are receptor- and site-specific, and not all sterols can mimic cholesterol’s role. The observed diastereospecificity, selective binding residues, and varied impact on receptor conformation provide a foundation for future drug design targeting GPCR–cholesterol interaction sites with synthetic allosteric modulators.

## 3 Discussion

### 3.1 Cholesterol-Interaction Motifs in Class A GPCRs

#### 3.1.1 Identification of the Cholesterol-Interaction Motifs

Studies in vitro have consistently highlighted the importance of cholesterol in GPCR stability and function. Despite clear evidence of cholesterol modulation across different GPCR classes,^15,34,42–45,51–53,60,61,249,302,314–318^ identifying cholesterol interaction sites remains challenging. These sites are often numerous and diverse, and not all are functionally relevant.

In a bioinformatics analysis^159^ of diverse X-ray and cryo-EM structures and in CG MD simulations of 28 different GPCR structures,^15^ it was shown that cholesterol engaged with multiple distinct regions within GPCRs. It was shown^159^ that cholesterol may bind almost promiscuously across the protein surface of a particular receptor, with specificity determined by the unique conformational states of each receptor.^44^ Thus, experimental structures, e.g., for 5-HTRs (see Table 1)^45^ and CG MD simulations for μOR^15,319^ and M2R^15^ or A_2A_R^131,234^ reveal that cholesterol interactions varied with the receptor’s activation-state (inactive vs fully activated state), showing the distinct fingerprint of cholesterol based on the conformational state. The cholesterol interaction sites in various GPCRs can range from motifs like **CCM** or **CRAC** and **CARC** to unique cholesterol-interaction sites specific to each GPCR subtype.^53,155^ It has been argued that cholesterol-interaction motifs—such as **CCM** and **CRAC/CARC**, which consist of motifs 5–13 amino acids long—predicted via bioinformatics or simulations, cannot reliably predict experimental sites and may yield false positives.^31,45,49,53,128^ However, **CCM** and **CRAC** motifs are likely to interact with cholesterol in GPCRs since they are thought to have a high binding affinity for cholesterol. Even cocrystallized cholesterol molecules may represent artifacts of membrane mimetics or detergent-based environments. A multitechnique approach (e.g., CG/AA MD simulations, site-directed mutagenesis, cryo-EM, cross-linking, and functional assays) is essential to differentiate incidental associations from functionally significant cholesterol binding. Moreover, some results across methodologies can be inconclusive or even contradictory, complicating mechanistic interpretation.

#### 3.1.2 Exploration of the Cholesterol-Interaction Motifs in the Selected Class A GPCRs

##### 3.1.2.1 General CCM and CRAC Motifs

In this review, we focused on 14 class A GPCRs, integrating evidence from structural biology, biochemical assays, mutagenesis, and multiple biophysical techniques to better understand the roles of cholesterol-receptor interactions. Some key findings include: β_2_AR and β_1_AR have cholesterol cocrystallized at IC TM2–TM4 **CCMs**, and cholesterol acts as a NAM, reducing β_2_AR signaling. However, the predicted IC TM7 **CRAC** motif is not essential for thermal stability, and the functional role of the IC TM2–TM4 **CCM** remains unclear. In A_2A_R, results are mixed: cholesterol’s effect on signaling is ambiguous, yet mutagenesis and ^19^F NMR suggest functional importance of IC TM2–TM4 **CCM** and IC TM7 **CRAC**, despite their absence in crystal structures. For 5-HT_1A_R, cholesterol enhances both signaling and agonist affinity, supported by cryo-EM structures showing cholesterol at IC TM2–TM4 **CCM** and functional data implicating an EC TM2 **CRAC** motif, though not directly observed in structures. OXTR displays clear cholesterol dependency: cholesterol enhances signaling and stability, and the EC TM4–TM5 **CCM** seen in X-ray structures was validated through mutagenesis and functional assays, including a novel ion channel-coupled assay. CCK_1_R exhibits strong cholesterol sensitivity, with signaling dependent on a specific IC TM3 **CRAC** motif supported by cross-linking, functional, and mutagenesis data. CCK_2_R, despite sharing the motif, remains cholesterol-insensitive, highlighting receptor-specific context. In CX3CR1, cryo-EM structures reveal three cholesterol-binding sites (IC TM2–TM4 **CCM**, IC TM3–TM5, IC TM5–TM6) where mutational disruption reduces signaling, confirming cholesterol as a PAM. CB_1_R shows reduced agonist binding in the presence of cholesterol, suggesting a NAM role, while CB_2_R shows the opposite effect. A **CRAC** motif at IC TM7 is critical for CB_1_R function, as shown by mutagenesis and MD simulations. For D_1_R, cholesterol increases cAMP signaling and agonist affinity. An EC TM5 **CRAC** motif and cholesterol binding at IC TM2–TM4 **CCM** are implicated in function, but only the latter is observed structurally. In NTS1R, cholesterol acts as a PAM, with EPR showing proximity, though predicted sites like IC TM2–TM4 **CCM** and IC TM6 **CRAC** remain unverified experimentally. In P2Y1R, despite multiple CHS molecules in structures, cholesterol does not affect function and predicted CRAC motifs were not functionally validated. A summary of motifs, experimental validations, and receptor-specific findings is provided in Table 3 for easier inspection.

**Table 3:** Experimental Evidence for the Presence and Functional Importance of **CCM** and **CRAC** Motifs in the Selected Class A GPCRs (See Also Supporting Information)

	**CCM**	**CRAC**
β_2_AR	IC TM2–TM4, X-ray inactive state	-
β_1_AR	IC TM2–TM4, X-ray inactive state	-
A_2A_R	IC TM2–TM4, MD simulations inactive state mutagenesis-functional assays	IC TM7, ^9^F NMR, MD simulations inactive state mutagenesis-functional assays
5-HT_1A_R	IC TM2–TM4, cryo-EM active state	EC TM2, MD simulations inactive state mutagenesis-functional assays
5-HT_2A_R	IC TM2–TM4, cryo-EM active state	-
5-HT_2A_R	IC TM2–TM4, X-ray inactive state	IC TM6/TM7, X-ray
CCK1R	-	IC TM3, cryo-EM, active state, cross-linking
CX3CR1	IC TM2–TM4, cryo-EM active state	-
CB_1_R	-	IC TM7 mutagenesis-functional assays
CB_2_R	IC TM2–TM4, X-ray inactive state	-

These examples demonstrate that cholesterol may act as a PAM or NAM, and that its effects are highly receptor specific. While bioinformatics can guide initial predictions, experimental validation is essential. Chemical cross-linking with reactive cholesterol analogs remains a promising strategy to identify and verify functionally relevant cholesterol-binding sites. Additionally, beyond direct interactions, cholesterol indirectly modulates GPCR activity by affecting membrane fluidity and microdomain localization (e.g., RhoR, 5-HT_1A_R, D1R), underlining its dual role as both a structural and allosteric component in membrane signaling. Despite progress, a comprehensive mechanistic framework explaining cholesterol’s modulation of class A GPCRs is still lacking. This review highlights the need for integrative approaches, combining structural, biochemical, and computational tools, to better elucidate these complex interactions.

##### 3.1.2.2 Distinct Cholesterol-Interaction Motifs

Importantly, additional cholesterol-interaction sites, beyond the commonly discussed **CCM** and **CRAC/CARC** motifs, have been experimentally observed in several class A GPCRs. These sites, often acting as distinct cholesterol-interaction hotspots, appear to contribute significantly to receptor function and warrant further investigation. For instance, low-resolution cryo-EM studies of RhoR have identified cholesterol molecules associated with EC TM6/TM7 and IC TM4, suggesting potential roles in receptor activation or stabilization. Similarly, in A_2A_R, cholesterol molecules were observed at EC TM2/TM3, EC TM5/TM6, and EC TM6/TM7 in X-ray structures—sites that were also supported by MD simulations, indicating that these extracellular positions may serve as regulatory cholesterol-interaction regions affecting ligand access or receptor conformation. In the fully activated 5-HT_1A_R, cryo-EM structures revealed cholesterol molecules associated with both the OBS and the receptor-Gi protein interface. The importance of these cholesterol molecules—particularly those with higher residence times, as identified in CG MD simulations—remains to be determined, but their potential role in stabilizing receptor-G protein coupling or modulating ligand binding warrants further study. In CX3CR1, the cryo-EM structure of the activated receptor revealed functionally significant cholesterol interactions at IC TM3/TM4/TM5 and IC TM5/TM6. Mutagenesis data already implicate these sites in signaling, but further biophysical and biochemical investigations could delineate the precise mechanisms by which these interactions contribute to CX3CR1 activation and Gi coupling.

### 3.2 Investigating Cholesterol’s Role in Class A GPCR Function: The Importance of Suitable Methods and Experimental Conditions

Several apparent contradictions in the literature underscore the complexity of class A GPCR-cholesterol interactions and highlight the need for additional research to fully elucidate the mechanisms involved. These inconsistencies may stem from differences in experimental conditions, receptor subtypes, or cellular and model membrane contexts across studies.

To validate the functional relevance of cholesterol-interaction sites, biochemical and functional assays must be appropriately adapted to mirror physiological conditions and allow meaningful comparisons across research efforts. For example, as discussed in Section 2.4.1, pharmacological data on A_2A_R remain difficult to interpret, likely due to the complex interplay between cholesterol and the surrounding lipid environment, which modulates receptor pharmacology. A similar issue arises in the case of 5-HT_1A_R (Section 2.5.3), where cholesterol was shown to inhibit receptor internalization. Yet, the efficiency of agonist-induced internalization differs significantly among neurons derived from various tissues and species, likely due to differences in lipid composition. These observations are in line with the broader understanding that cholesterol can engage in synergistic or competitive cobinding with other lipids in natural membranes, as demonstrated by MD simulations of 5-HT_1A_R.^33^

To reach a consensus across different structural and functional studies, a robust, integrative methodology is essential for identifying and validating cholesterol interaction sites (Figure 12). Structural biology techniques such as cryo-EM, solution NMR, and photo-cross-linking with reactive cholesterol analogs, in conjunction with MD simulations, should be employed in membrane-mimetic environments. The use of phospholipid bilayers, which closely replicate the structural properties of natural membranes, is critical for such investigations. Suitable mimetics include bicelles (e.g., DMPC with short-chain PLs or sphingolipids and cholesterol)^320,321^ nanodiscs,^322,323^ and liposomes^206^—all of which facilitate both structural elucidation and functional analysis of GPCRs. Notably, engineered nanodiscs have emerged as powerful fluorescent sensors for monitoring protein–lipid interactions.^324^

**Figure 12:**
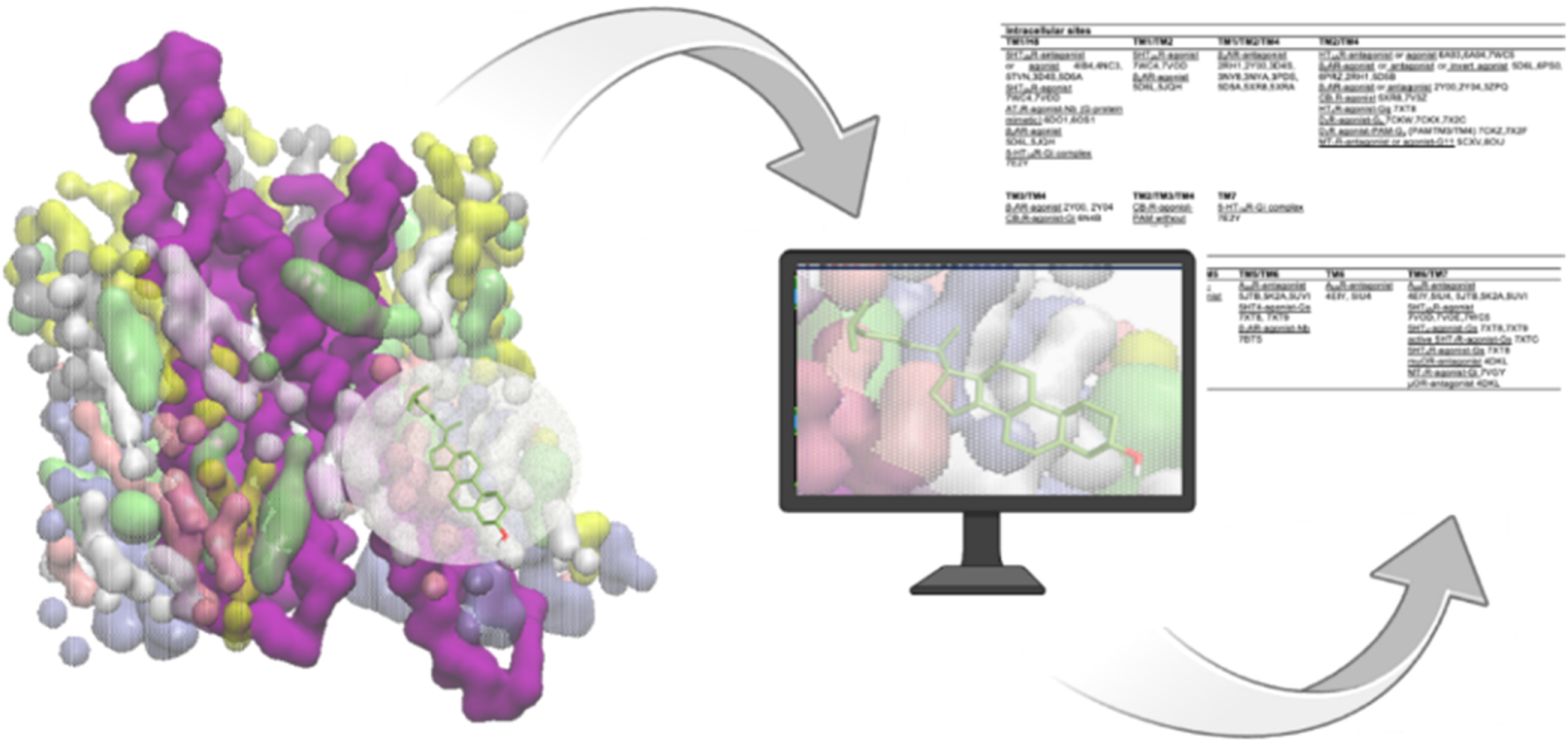
An integrative methodology is required to explain the functional importance of cholesterol interaction sites after their identification.

Although there is often strong agreement between cholesterol-interaction sites observed experimentally and those predicted via MD simulations, discrepancies persist. These can be attributed to the intrinsic flexibility and conformational heterogeneity of GPCRs, as well as limitations in sampling, force field accuracy, and membrane modeling in simulations. For instance, CG MD simulations in ref 33 explored the association of cholesterol’s α- and β-faces with 5-HT1AR using the Martini 2.2 model. However, despite custom mapping, the resolution was insufficient to distinguish between the interactions of the two cholesterol faces—an issue also seen in simulations of SMOR (Section 1.2.2). Higher-resolution approaches, such as all-atom MD simulations or improved coarse-grained parameters available in Martini 3, may be necessary to overcome these limitations and achieve a more accurate representation of cholesterol–GPCR interactions.^325^

### 3.3 Comparison of Cholesterol-Interaction Sites and Binding Sites of Allosteric Inhibitors

The comparison of class A GPCR structures with cocrystallized cholesterol, which may act as a natural allosteric modulator, with structures of the same receptor or a different receptor bound to a synthetic allosteric modulator offers valuable insight into the molecular mechanisms of cholesterol-mediated modulation. Such comparisons may also reveal candidate sites for drug targeting. Several examples, mentioned below, illustrate this principle, where an allosteric antagonist or modulator binds to the same site as cholesterol in another GPCR structure.

In both the fully active state (PDB ID: 6H7J)^196^ and the intermediate-active conformation (PDB ID 2Y03)^197^ of the isoprenaline−β_1_AR complex, a cholesterol molecule is resolved at the IC TM3/TM4/TM5 interface, overlapping with key microswitch motifs such as PIF, DRY, and NPxxY. These motifs are essential components of the receptor’s allosteric signaling network.^224,227,326^ Intriguingly, this same region is targeted by several synthetic allosteric modulators in other class A GPCRs. For instance, PAMs of GPR40 ^327^ and D_1_R,^328^ or NAMs of β_2_AR,^329^ or CB_1_R,^330^ for example, in the X-ray structure of the neutral antagonist alprenolol–NAM AS408−β_2_AR complex (PDB ID 6OBA^329^), and the X-ray structure of the agonist MK-8666–PAM AP8–GPR40 complex (PDB ID 5TZY^331^) which exist in an intermediate-active conformation. Although these modulators bind in a region overlapping the cholesterol site in β_1_AR, they exhibit diverse functional effects—either enhancing (PAM) or inhibiting (NAM) receptor activity. Their mechanism often involves impeding the sliding or outward movement of TM5, a critical step in receptor activation. The functional direction of modulation depends on how this restriction affects the receptor’s transition to its active state.

In the X-ray structure of P2Y1R bound to the orthosteric antagonist MRS2500 and the allosteric antagonist BPTU (PDB ID 4XNV^284^), a CHS molecule is cocrystallized. BPTU binds to an allosteric site at the EC TM2/TM3 interface, located at the boundary between the receptor and lipid bilayer. This pocket corresponds to a cholesterol-interaction cavity observed in A_2A_R, suggesting conserved lipid-accessible allosteric modulation sites across GPCRs (see Table 1).

A clear example of allosteric modulation via cholesterol is seen in CB_1_R. In the intermediate-active state of the CB_1_R–CP55940–PAM ZCZ011 complex (PDB ID 7FEE^294^), a cholesterol molecule is cocrystallized at the IC TM2–TM4 **CCM**. In contrast, in the CB_1_R–CP55940–NAM ORG27569 structure (PDB ID 6KQI^296^), the ORG27569 molecule occupies the same IC TM2–TM4 **CCM** (see Section 2.9.1) site, effectively displacing cholesterol. This replacement blocks the receptor’s transition to the fully active conformation, functioning as a NAM. These observations support a model in which cholesterol may naturally occupy the same site as ORG27569 but is displaced during receptor activation, suggesting that cholesterol and synthetic NAMs like ORG27569 can compete for the same modulatory site (see also Figure 13).

**Figure 13:**
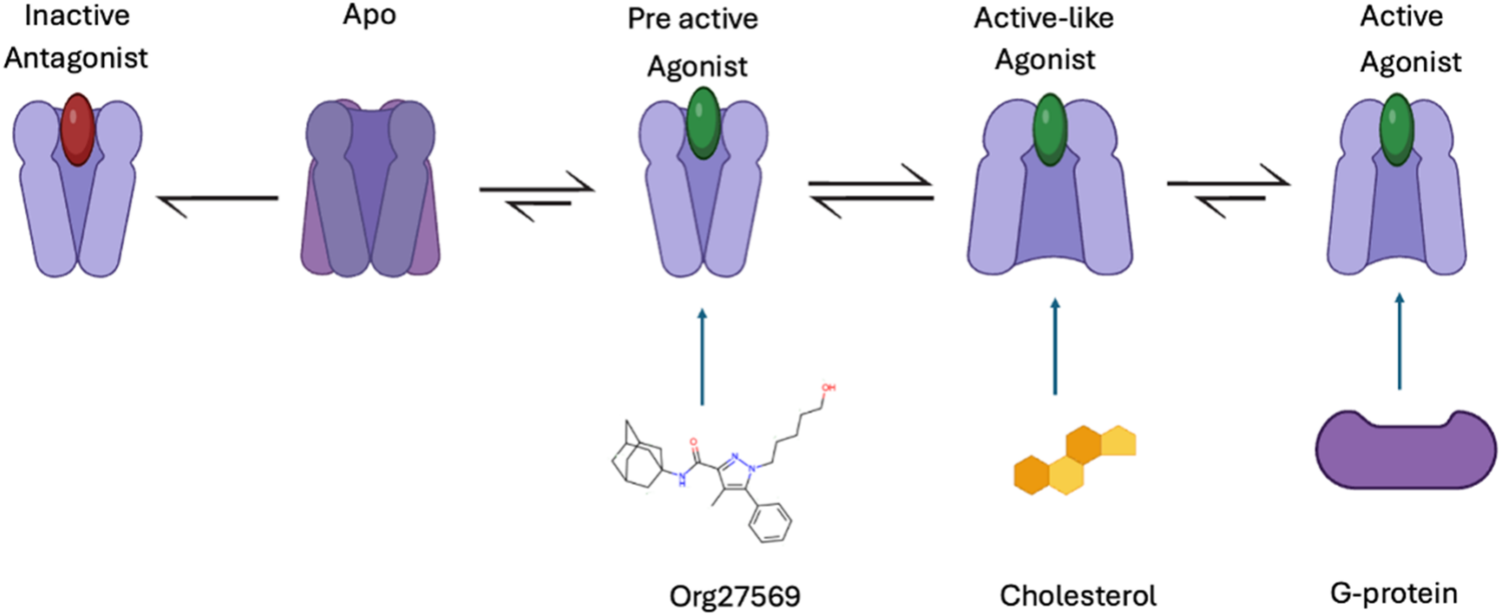
Activation and allosteric modulation model of CB_1_R: Org27569, cholesterol, and G protein can stabilize and increase the conformation population of receptors in preactive, active-like, and active states, respectively.

These examples highlight that cholesterol not only serves a structural or membrane-organizing role but can act as a functional modulator, either stabilizing inactive, intermediate, or preactive conformations. Understanding these cholesterol-interaction hotspots and their overlap with synthetic modulators opens new avenues for the design of lipid-site-targeting drugs in class A GPCRs.

### 3.4 Cholesterol-Interaction Sites as Potential Drug Targets

Synthetic allosteric modulators^4,331^ can be rationally designed to bind class A GPCR subtypes at cholesterol-interaction sites with long residence times,^249^ which often include surface-exposed driver or modulator residues.^332^ Targeting these allosteric sites offers a path to desired drug selectivity,^249,333^ as such sites are typically less conserved than the orthosteric binding sites, and their defining motifs can be distinctive across GPCR subtypes. Examples from the selected set of receptors and a novel one are provided below.

In the cryo-EM structure of CCK8-CCK1R-G protein complexes (PDB IDs 7MBY,^280^ 7EZM^281^), multiple cholesterol-interaction sites were identified (see Section 2.7.1). Cholesterol interacts with Y140^3.51^ at IC TM3 **CRAC**, which is proximal to IL2, altering the binding of agonist CCK8 and the efficiency of the receptor to engage Gq/11 proteins. Since cholesterol disrupts normal receptor-G protein coupling, PAMs that bind outside the deep orthosteric binding site^114^ may restore signaling—a promising strategy for obesity treatment.^334^

CX3CR1, an emerging therapeutic target, is difficult to drug due to its chemokine promiscuity. Currently, only a nanobody and the small-molecule inhibitor AZD8797 are in phase I clinical trials.^311^ The three cholesterol-interaction sites that stabilize CX3CR1 activation (Section 2.12.1) present promising allosteric targets for new inhibitors.

Targeting the EC TM4/TM5 **CCM** in OXTR, displacing cholesterol and destabilizing OBS through allosteric antagonists, can have a therapeutic interest in the treatment of male sexual disorders, assisted reproductive technologies, and spontaneous premature labor (see Section 2.6.1).

The structure of the active D_1_R (PDB ID 7X2F^335^) which corresponds to the agonist dopamine–D_1_R–Gs complex provides a template for the rational design of D_1_R-selective agonists and PAMs. This structure also includes cocrystallized cholesterol molecules at IC TM2–TM4 **CCM** and IC TM4, which likely stabilize LY3154207 binding. These cholesterol-interaction sites could be exploited by allosteric inhibitors to dampen D_1_R signaling, potentially offering treatments for schizophrenia, psychosis, levodopa-induced dyskinesia (in PD), and addiction.

In CB_1_R, NAM ORG27569 can be replaced by cholesterol to allow the transition from a preactivated to a fully activated state (Figure 13). Such stable and long-standing cholesterols can be used for the design of allosteric modulators that can bind CB_1_R by entering through lipid pathways^296,330^ and reduce this receptor signaling needed in disorders like obesity and metabolic syndrome, cardiovascular disorders, and psychiatric disorders (e.g., schizophrenia, anxiety, depression).

The recently solved X-ray structure of apo-GPR6 (PDB ID 8TF5 ^336^), an orphan receptor of the MECA GPCR cluster (melanocortin/EDG/cannabinoid/adenosine), reveals 16 cocrystallized annular lipids, including a cholesterol molecule interacting with IC TM2–TM4 **CCM** and engaged W193^4.50^. GPR6, highly expressed in the CNS, shows promise as a nondopaminergic target in PD, based on evidence from knockout models with reduced involuntary movements.^337^ The cholesterol-interaction cavity is stabilized by additional interactions (e.g., with T116^2.48^ and V109^2.41^) and may be targeted by inverse agonists or allosteric modulators for therapeutic purposes (Figure 14).

**Figure 14:**
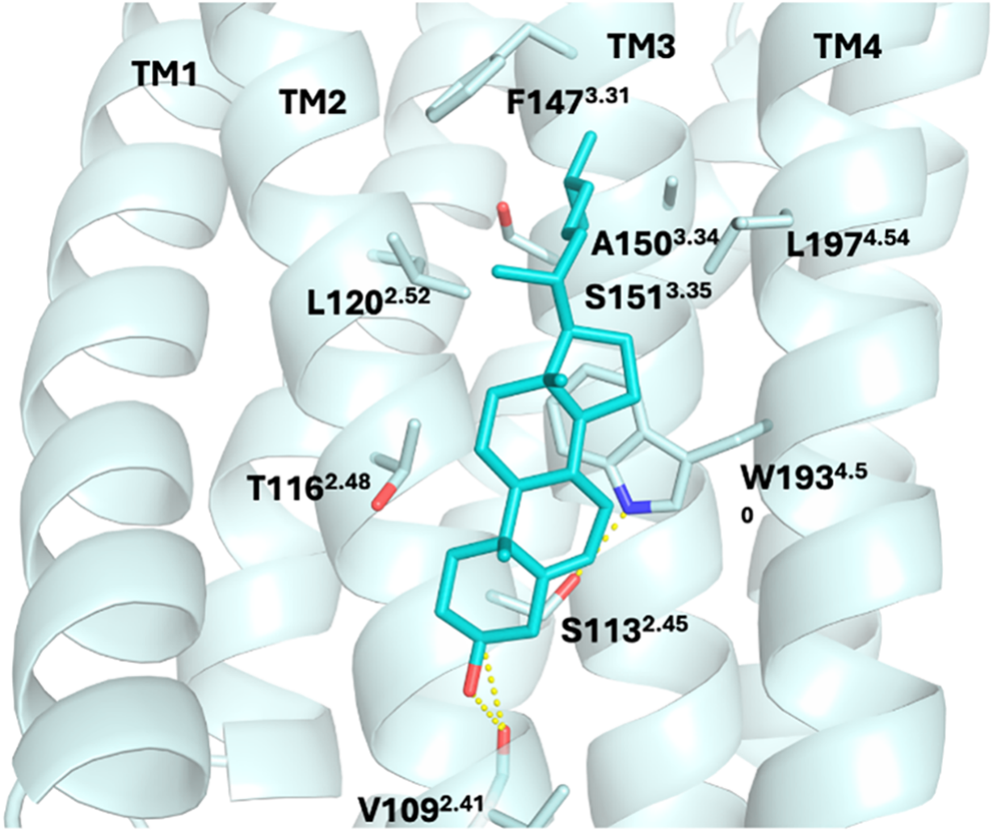
Cholesterol interaction site in the crystal structure of apo-GPR6 (PDB ID 8TF5 ^336^). A cholesterol molecule (light green sticks) is observed at the intracellular interface between TM2 and TM4. Residues within 4.5 Å of cholesterol are shown in gray sticks, indicating direct contact. Hydrogen bonds are represented as yellow dashed lines.

Even in natural tissues, many GPCRs can form dimers or oligomers^8,17,18^ which can have a crucial role in both physiological and pathological processes.^19^ For some GPCRs, a dynamic equilibrium between monomers and dimers or other oligomers has been noted.^17,20,21^ The development of allosteric drugs that bind to the appropriate cholesterol-interaction sites, inhibiting the oligomerization, which is critical for receptor function, is an intriguing area of research.

### 3.5 Interpreting the Effect of Cholesterol In Vivo

To provide a comprehensive description of the mechanism of the effect of cholesterol on GPCR function, both in vitro and in vivo data must be incorporated and related. Therefore, after identification and confirmation of stable cholesterol-interaction sites in a physiologically relevant environment, as important in vitro, it will be critical to consider their physiological relevance in vivo. However, as regards the cholesterol-interaction sites, it is hard to directly observe cholesterol association in vivo. For example, seeing cholesterol associated with a **CCM** or a **CRAC** motif in animal models is technically impossible with today’s methods. Most in vivo evidence is indirect changes in receptor function consistent with cholesterol interaction being disrupted.^248^ Results from functional assays (mutations, cholesterol depletion + phenotypes) are what are mainly available and accepted as evidence from in vivo experiments. Mutagenesis studies targeting a motif in living animals may be possible in the future. Therefore, interpreting the effect of cholesterol in vivo is challenging, and considerable additional work is necessary to clarify the role of cholesterol in class A GPCRs’ signaling and function in vivo.

## Supplementary Material



## Notes

Phylogenetic analysis of class A GPCRs (Figure S1); with multiple sequence analysis of the selected class A GPCRs focusing on comparison of certain CCM, CRAC motifs, and the NPxxY, DRY, CWxP motifs (Figure S2); with comparison of predicted conserved CCM and CRAC motifs between selected class A GPCRs (Table S1); and methods of sequence analysis and relevant references (PDF)

## Abbreviations

AR: adenosine or adrenergic receptor
arr: arrestin
AA: all-atom
CBR: cannabinoid receptor
CG: coarse-grained
CHAPS: 3-[(3-cholamidopropyl)­dimethylammonio]-1-propanesulfonate
CKR: chemokine receptor
CCKR: cholecystokinin receptor
CCM: Cholesterol Consensus Motif
CRAC: Cholesterol Recognition Amino Acids Consensus
cryo-EM: cryogenic electron microscopy
DSC: differential scanning calorimetry
DR: dopamine receptor
EC: extracellular
EL: exracellular loop
FRET: fluorescence resonance energy transfer (FRET)
GM1: monosialotetrahexosylganglioside
GPCR: G protein-coupled receptor
GRK: GPCR kinase
GUV: giant unilamellar vesicle
GUP: unilamellar protein-vesicle
IC: intracellular
IL: intracellular loop
ITC: isothermal titration calorimetry
ICCR: ion channel-coupled receptor
5-HT: 5-hydroxytryptamine
LCP-Tm: lipid cubic phase melting temperature
MD: molecular dynamics
MS: mass spectrometry
Nb: nanobody
NMR: nuclear magnetic resonance
nMS: native mass spectrometry
NTS: neurotensin
OBS: orthosteric binding site
PA: phosphatidic acid
PC: phosphatidylcholine
PE: phosphatidylethanolamine
PD: Parkinson’s disease
PI: phosphatidylinositol
PL: phospholipid
PLinPC: 1-palmitoyl-2-linoleoyl-*sn*-glycero-3-phosphocholine
POPC: 1-palmitoyl-2-oleoyl-*sn*-glycero-3-phosphocholine
POPE: 1-palmitoyl-2-oleoyl-*sn*-glycero-3-phosphoethanolamine
POPS: 1-palmitoyl-2-oleoyl-*sn*-glycero-3-phosphoserine
PoxnoPC: 1-palmitoyl-2-(9′-oxo-nonanoyl)-*sn*-glycero-3-phosphocholine
PtdIns4P: phosphatidylinositol 4-phosphate
PtdIns­(4,5)­P_2_ or PI­(4,5)­P_2_ or PIP2: phosphatidylinositol 4,5-diphosphate
PS: phosphatidylserine
P2YR: purinergic P2Y receptor
Rho: rhodopsin
ROS: rod outer segment
SM: sphingomyelin
RMSD: root-mean-square deviation
SMF: single-molecule fluorescence
ss: solid state
SPR: surface plasmon resonance
TM: transmembrane
UV–vis: ultraviolet–visible

## References

[1] van
Meer G., Voelker D. R., Feigenson G. W. (2008). Membrane lipids: where they are and
how they behave. Nat. Rev. Mol. Cell Biol..

[2] Hilger D., Masureel M., Kobilka B. K. (2018). Structure
and dynamics of GPCR signaling
complexes. Nat. Struct. Mol. Biol..

[3] Manglik, A. ; Kobilka, B. The role of protein dynamics in GPCR function: Insights from the β2AR and rhodopsin. Curr. Opin. Cell Biol. 2014 27 136 10.1016/j.ceb.2014.01.008. 24534489 PMC3986065

[4] Thal D. M., Glukhova A., Sexton P. M., Christopoulos A. (2018). Structural
insights into G-protein-coupled receptor allostery. Nature.

[5] Lane J. R., May L. T., Parton R. G., Sexton P. M., Christopoulos A. (2017). A kinetic
view of GPCR allostery and biased agonism. Nat.
Chem. Biol..

[6] Smith J. S., Lefkowitz R. J., Rajagopal S. (2018). Biased signalling: from simple switches
to allosteric microprocessors. Nat. Rev. Drug
Discovery.

[7] Attwood T. K., Findlay J. B. C. (1994). Fingerprinting G-protein-coupled receptors. Protein Eng., Des. Sel..

[8] Gahbauer S., Böckmann R. A. (2016). Membrane-Mediated
Oligomerization of G Protein Coupled
Receptors and Its Implications for GPCR Function. Front. Physiol..

[9] Fried S. D. E., Lewis J. W., Szundi I. (2021). Membrane Curvature Revisited—the
Archetype of Rhodopsin Studied by Time-Resolved Electronic Spectroscopy. Biophys. J..

[10] Soubias O., Teague W. E., Hines K. G., Mitchell D. C., Gawrisch K. (2010). Contribution
of Membrane Elastic Energy to Rhodopsin Function. Biophys. J..

[11] Wang Y., Botelho A. V., Martinez G. V., Brown M. F. (2002). Electrostatic
Properties
of Membrane Lipids Coupled to Metarhodopsin II Formation in Visual
Transduction. J. Am. Chem. Soc..

[12] Mitchell D.
C., Straume M., Miller J. L., Litman B. J. (1990). Modulation of metarhodopsin
formation by cholesterol-induced ordering of bilayer lipids. Biochemistry.

[13] Niu S. L., Mitchell D. C., Litman B. J. (2002). Manipulation
of Cholesterol Levels
in Rod Disk Membranes by Methyl-β-cyclodextrin. J. Biol. Chem..

[14] Soubias O., Sodt A. J., Teague W. E., Hines K. G., Gawrisch K. (2023). Physiological
changes in bilayer thickness induced by cholesterol control GPCR rhodopsin
function. Biophys. J..

[15] Sejdiu B. I., Tieleman D. P. (2020). Lipid-Protein Interactions
Are a Unique Property and
Defining Feature of G Protein-Coupled Receptors. Biophys. J..

[16] Yen H. Y., Hoi K. K., Liko I. (2018). PtdIns­(4,5)­P2
stabilizes
active states of GPCRs and enhances selectivity of G-protein coupling. Nature.

[17] Kasai R. S., Kusumi A. (2014). Single-molecule imaging
revealed dynamic GPCR dimerization. Curr. Opin.
Cell Biol..

[18] Chapter 6—Molecular Determinants of GPCR Oligomerization. In GPCRs; Mohole, M. ; Kumar, G. A. ; Sengupta, D. ; Chattopadhyay, A. ; Jastrzebska, B. ; Park, P. S.-H. , Eds.; Elsevier, 2020; pp 97–108.

[19] Cvejic S., Devi L. A. (1997). Dimerization of
the δ Opioid Receptor. J. Biol. Chem..

[20] Calebiro D., Sungkaworn T. (2018). Single-Molecule Imaging of GPCR Interactions. Trends Pharmacol. Sci..

[21] Calebiro D., Rieken F., Wagner J. (2013). Single-molecule analysis
of fluorescently labeled G-protein–coupled receptors reveals
complexes with distinct dynamics and organization. Proc. Natl. Acad. Sci. U.S.A..

[22] Kumar G. A., Chattopadhyay A. (2016). Cholesterol:
An evergreen molecule in biology. Biomed. Spectrosc.
Imaging.

[23] Ranadive G. N., Lala A. K. (1987). Sterol-phospholipid interaction in model membranes:
role of C5-C6 double bond in cholesterol. Biochemistry.

[24] de
Meyer F., Smit B. (2009). Effect of cholesterol on the structure
of a phospholipid bilayer. Proc. Natl. Acad.
Sci. U.S.A..

[26] Kim J. H., Singh A., Del Poeta M., Brown D. A., London E. (2017). The effect
of sterol structure upon clathrin-mediated and clathrin-independent
endocytosis. J. Cell Sci..

[27] Villar V. A. M., Cuevas S., Zheng X., Jose P. A. (2016). Localization and
signaling of GPCRs in lipid rafts. Methods Cell
Biol..

[28] Bodzęta A., Scheefhals N., MacGillavry H. D. (2021). Membrane trafficking and positioning
of mGluRs at presynaptic and postsynaptic sites of excitatory synapses. Neuropharmacology.

[29] Wijetunge L. S., Till S. M., Gillingwater T. H., Ingham C. A., Kind P. C. (2008). mGluR5
Regulates Glutamate-Dependent Development of the Mouse Somatosensory
Cortex. J. Neurosci..

[30] Roth A. F., Wan J., Bailey A. O. (2006). Global Analysis of Protein Palmitoylation
in Yeast. Cell.

[31] Bukiya A. N., Dopico A. M. (2017). Common structural
features of cholesterol binding sites
in crystallized soluble proteins. J. Lipid Res..

[32] Hedger G., Koldsø H., Chavent M., Siebold C., Rohatgi R., Sansom M. S. P. (2019). Cholesterol Interaction Sites on the Transmembrane
Domain of the Hedgehog Signal Transducer and Class F G Protein-Coupled
Receptor Smoothened. Structure.

[33] Mohole M., Sengupta D., Chattopadhyay A. (2022). Synergistic
and Competitive Lipid
Interactions in the Serotonin1A Receptor Microenvironment. ACS Chem. Neurosci..

[34] Jafurulla, M. ; Aditya Kumar, G. ; Rao, B. D. ; Chattopadhyay, A. A Critical Analysis of Molecular Mechanisms Underlying Membrane Cholesterol Sensitivity of GPCRs. In Cholesterol Modulation of Protein Function; Springer International Publishing, 2019; pp 21–52 10.1007/978-3-030-04278-3_2. 30649754

[35] Nagle J. F. (2021). Measuring
the bending modulus of lipid bilayers with cholesterol. Phys. Rev. E.

[36] Chakraborty S., Doktorova M., Molugu T. R. (2020). How cholesterol stiffens
unsaturated lipid membranes. Proc. Natl. Acad.
Sci. U.S.A...

[37] Needham D., McIntosh T. J., Evans E. (1988). Thermomechanical and transition properties
of dimyristoylphosphatidylcholine/cholesterol bilayers. Biochemistry.

[38] Kollmitzer B., Heftberger P., Rappolt M., Pabst G. (2013). Monolayer spontaneous
curvature of raft-forming membrane lipids. Soft
Matter..

[39] Subczynski W. K., Wisniewska A., Yin J. J., Hyde J. S., Kusumi A. (1994). Hydrophobic
Barriers of Lipid Bilayer Membranes Formed by Reduction of Water Penetration
by Alkyl Chain Unsaturation and Cholesterol. Biochemistry.

[40] Feigenson G. W. (2006). Phase behavior
of lipid mixtures. Nat. Chem. Biol..

[41] Pöhnl M., Trollmann M. F. W., Böckmann R. A. (2023). Nonuniversal impact of cholesterol
on membranes mobility, curvature sensing and elasticity. Nat. Commun..

[42] Oates J., Watts A. (2011). Uncovering the intimate relationship
between lipids, cholesterol
and GPCR activation. Curr. Opin. Struct. Biol..

[43] Sarkar, P. ; Chattopadhyay, A. Cholesterol in GPCR Structures: Prevalence and Relevance. J. Membr. Biol. 2022 255 99 10.1007/s00232-021-00197-8. 34365520

[44] Gimpl, G. Interaction of G protein coupled receptors and cholesterol. Chem. Phys. Lipids. 2016 199 61 10.1016/j.chemphyslip.2016.04.006. 27108066

[45] Moreau C. J., Audic G., Lemel L., García-Fernández M. D., Nieścierowicz K. (2023). Interactions of cholesterol molecules with GPCRs in
different states: A comparative analysis of GPCRs’ structures. Biochim. Biophys. Acta, Biomembr..

[46] Gimpl, G. ; Burger, K. ; Fahrenholz, F. Cholesterol as modulator of receptor function. Biochemistry 1997 36 10959 10.1021/bi963138w. 9283088

[47] Oates J., Faust B., Attrill H., Harding P., Orwick M., Watts A. (2012). The role of cholesterol
on the activity and stability of neurotensin
receptor 1. Biochim. Biophys. Acta, Biomembr..

[48] Serdiuk T., Manna M., Zhang C. (2022). A cholesterol analog
stabilizes the human β2-adrenergic receptor nonlinearly with
temperature. Sci. Signal..

[49] Corradi V., Sejdiu B. I., Mesa-Galloso H. (2019). Emerging Diversity in
Lipid–Protein Interactions. Chem. Rev..

[50] Leonard A. N., Lyman E. (2021). Activation of G-protein-coupled receptors is thermodynamically linked
to lipid solvation. Biophys. J..

[51] Sarkar P., Chattopadhyay A. (2020). Cholesterol interaction motifs in G protein-coupled
receptors: Slippery hot spots?. Wiley Interdiscip.
Rev.: Syst. Biol. Med..

[52] Lee A. G. (2018). A Database
of Predicted Binding Sites for Cholesterol on Membrane Proteins, Deep
in the Membrane. Biophys. J..

[53] Jafurulla, M. ; Tiwari, S. ; Chattopadhyay, A. Identification of cholesterol recognition amino acid consensus (CRAC) motif in G-protein coupled receptors. Biochem. Biophys. Res. Commun. 2011 404 569 10.1016/j.bbrc.2010.12.031. 21146498

[54] Corey R. A., Vickery O. N., Sansom M. S. P., Stansfeld P. J. (2019). Insights
into Membrane Protein–Lipid Interactions from Free Energy Calculations. J. Chem. Theory Comput..

[55] Ansell T. B., Curran L., Horrell M. R. (2021). Relative Affinities
of Protein–Cholesterol Interactions from Equilibrium Molecular
Dynamics Simulations. J. Chem. Theory Comput..

[56] Genheden, S. ; Essex, J. W. ; Lee, A. G. G protein coupled receptor interactions with cholesterol deep in the membrane. Biochim. Biophys. Acta, Biomembr. 2017 1859 268 10.1016/j.bbamem.2016.12.001. 27919726

[57] Lee J. Y., Lyman E. (2012). Predictions for Cholesterol Interaction
Sites on the A2A Adenosine
Receptor. J. Am. Chem. Soc..

[58] Salari R., Joseph T., Lohia R., Hénin J., Brannigan G. (2018). A Streamlined, General Approach for
Computing Ligand
Binding Free Energies and Its Application to GPCR-Bound Cholesterol. J. Chem. Theory Comput..

[59] Levental I., Lyman E. (2023). Regulation of membrane
protein structure and function by their lipid
nano-environment. Nat. Rev. Mol. Cell Biol..

[60] Paila Y. D., Chattopadhyay A. (2009). The function of G-protein coupled receptors and membrane
cholesterol: Specific or general interaction?. Glycoconjugate J..

[61] Paila Y. D., Tiwari S., Chattopadhyay A. (2009). Are specific
nonannular cholesterol
binding sites present in G-protein coupled receptors?. Biochim. Biophys. Acta, Biomembr..

[62] Lorente, J. S. ; Sokolov, A. V. ; Ferguson, G. ; Schiöth, H. B. ; Hauser, A. S. ; Gloriam, D. E. GPCR drug discovery: new agents, targets and indications. Nat. Rev. Drug Discovery 2025 24 458 10.1038/s41573-025-01139-y. 40033110

[63] Santos R., Ursu O., Gaulton A. (2017). A comprehensive map
of molecular drug targets. Nat. Rev. Drug Discovery.

[64] Ursu O., Holmes J., Knockel J. (2017). DrugCentral: online
drug compendium. Nucleic Acids Res..

[65] Alexander S. P. H., Christopoulos A., Davenport A. P. (2021). The concise guide to pharmacology 2021/22:
G protein-coupled receptors. Br. J. Pharmacol..

[66] Kobilka, B. The structural basis of G-protein-coupled receptor signaling (nobel lecture). Angew. Chem., Int. Ed. 2013 52 6380 10.1002/anie.201302116. PMC4031317 23650120

[67] Weis W. I., Kobilka B. K. (2008). Structural insights into G-protein-coupled receptor
activation. Curr. Opin. Struct. Biol..

[68] Weis W. I., Kobilka B. K. (2018). The Molecular Basis of G Protein–Coupled Receptor
Activation. Annu. Rev. Biochem..

[69] Venkatakrishnan A. J., Deupi X., Lebon G. (2016). Diverse activation pathways
in class A GPCRs converge near the G-protein-coupling region. Nature.

[70] Ragnarsson L., Andersson Å., Thomas W. G., Lewis R. J. (2019). Mutations in the
NPxxY motif stabilize pharmacologically distinct conformational states
of the α1B - and β2-adrenoceptors. Sci. Signal..

[71] Bologna Z., Teoh J. p., Bayoumi A. S., Tang Y., Kim I. m. (2017). Biased
G Protein-Coupled Receptor Signaling: New Player in Modulating Physiology
and Pathology. Biomol. Ther..

[72] Hauser A. S., Kooistra A. J., Munk C. (2021). GPCR activation mechanisms
across classes and macro/microscales. Nat. Struct.
Mol. Biol..

[73] Unal H., Karnik S. S. (2012). Domain coupling in GPCRs: the engine
for induced conformational
changes. Trends Pharmacol. Sci..

[74] Deupi X., Standfuss J. (2011). Structural
insights into agonist-induced activation
of G-protein-coupled receptors. Curr. Opin.
Struct. Biol..

[75] Rosenbaum, D. M. ; Rasmussen, S. G. F. ; Kobilka, B. K. The structure and function of G-protein-coupled receptors. Nature 2009 459 356 10.1038/nature08144. 19458711 PMC3967846

[76] Manglik A., Kruse A. C., Kobilka T. S. (2012). Crystal structure of
the μ-opioid receptor bound to a morphinan antagonist. Nature.

[77] Felce J. H., Latty S. L., Knox R. G. (2017). Receptor Quaternary
Organization Explains G Protein-Coupled Receptor Family Structure. Cell Rep..

[78] Bouvier M., Hébert T. E. (2014). CrossTalk
proposal: Weighing the evidence for Class
A GPCR dimers, the evidence favours dimers. J. Physiol..

[79] Pin J. P., Kniazeff J., Prézeau L., Liu J. F., Rondard P. (2019). GPCR interaction
as a possible way for allosteric control between receptors. Mol. Cell. Endocrinol..

[80] Whorton M. R., Bokoch M. P., Rasmussen S. G. F. (2007). A monomeric G protein-coupled
receptor isolated in a high-density lipoprotein particle efficiently
activates its G protein. Proc. Natl. Acad. Sci.
U.S.A..

[81] Zhou X. E., He Y., de Waal P. W. (2017). Identification of Phosphorylation Codes
for Arrestin Recruitment by G Protein-Coupled Receptors. Cell.

[82] Huang W., Masureel M., Qu Q. (2020). Structure
of the neurotensin
receptor 1 in complex with β-arrestin 1. Nature.

[83] Staus D. P., Hu H., Robertson M. J. (2020). Structure of the M2 muscarinic receptor−β-arrestin
complex in a lipid nanodisc. Nature.

[84] Kang Y., Zhou X. E., Gao X. (2015). Crystal structure of
rhodopsin bound to arrestin by femtosecond X-ray laser. Nature.

[85] Gurevich V. V., Gurevich E. V. (2019). GPCR Signaling Regulation:
The Role of GRKs and Arrestins. Front. Pharmacol..

[86] Gurevich V. V., Gurevich E. V. (2018). GPCRs and Signal Transducers: Interaction Stoichiometry. Trends Pharmacol. Sci..

[87] Armando S., Quoyer J., Lukashova V. (2014). The chemokine CXC4 and
CC2 receptors form homo- and heterooligomers that can engage their
signaling G-protein effectors and βarrestin. FASEB J..

[88] Ge B., Lao J., Li J., Chen Y., Song Y., Huang F. (2017). Single-molecule
imaging reveals dimerization/oligomerization of CXCR4 on plasma membrane
closely related to its function. Sci. Rep..

[89] White J. F., Grodnitzky J., Louis J. M. (2007). Dimerization of the
class A G protein-coupled neurotensin receptor NTS1 alters G protein
interaction. Proc. Natl. Acad. Sci. U.S.A..

[90] Möller J., Isbilir A., Sungkaworn T. (2020). Single-molecule analysis
reveals agonist-specific dimer formation of μ-opioid receptors. Nat. Chem. Biol..

[91] Ingólfsson H.
I., Arnarez C., Periole X., Marrink S. J. (2016). Computational ‘microscopy’
of cellular membranes. J. Cell Sci..

[92] Ingólfsson H. I., Melo M. N., van Eerden F. J., Arnarez C., Lopez C. A., Wassenaar T. A., Periole X., de Vries A. H., Tieleman D. P., Marrink S. J. (2014). Lipid organization of the plasma membrane. J. Am. Chem. Soc..

[93] Marrink S. J., Corradi V., Souza P. C. T., Ingólfsson H. I., Tieleman D. P., Sansom M. S. P. (2019). Computational Modeling of Realistic
Cell Membranes. Chem. Rev..

[94] Dawaliby R., Trubbia C., Delporte C., Masureel M., VanAntwerpen P., Kobilka B. K., Govaerts C. (2016). Allosteric
regulation of GPCR activity
by phospholipids. Nat. Chem. Biol..

[95] Guixa-Gonzalez R., Javanainen M., Gomez-Soler M., Cordobilla B., Domingo J. C., Sanz F., Pastor M., Ciruela F., Martinez-Seara H., Selent J. (2016). Membrane omega-3 fatty acids modulate
the oligomerisation kinetics of adenosine A2A and dopamine D2 receptors. Sci. Rep..

[96] Allen J. A., Halverson-Tamboli R. A., Rasenick M. M. (2007). Lipid raft microdomains and neurotransmitter
signalling. Nat. Rev. Neurosci..

[97] Waltenspühl Y., Schöppe J., Ehrenmann J., Kummer L., Plückthun A. C. (2020). Crystal
Structure of the Human Oxytocin Receptor. Sci.
Adv..

[98] Zocher M., Zhang C., Rasmussen S. G. F., Kobilka B. K., Müller D. J. (2012). Cholesterol
increases kinetic, energetic, and mechanical stability of the human
β_2_-adrenergic receptor. Proc.
Natl. Acad. Sci. U.S.A..

[99] Saxena R., Chattopadhyay A. (2012). Membrane cholesterol stabilizes the human serotonin1A
receptor. Biochim. Biophys. Acta, Biomembr..

[100] Qi X., Friedberg L., Bose-Boyd R. D., Long T., Li X. (2020). Sterols in
an Intramolecular Channel of Smoothened Mediate Hedgehog Signaling. Nat. Chem. Biol..

[101] Yeliseev A., Iyer M. R., Joseph T. T. (2021). Cholesterol
as a modulator of cannabinoid receptor CB2 signaling. Sci. Rep..

[102] Zheng H., Pearsall E. A., Hurst D. P., Zhang Y., Chu J., Zhou Y., Reggio P. H., Loh H. H., Law P. Y. (2012). Palmitoylation
and Membrane Cholesterol Stabilize μ-Opioid Receptor Homodimerization
and G Protein Coupling. BMC Cell Biol..

[103] Ganguly S., Clayton A. H. A., Chattopadhyay A. (2011). Organization
of higher- order oligomers of the serotonin1A receptor explored utilizing
homo-FRET in live cells. Biophys. J..

[104] Cherezov V., Rosenbaum D. M., Hanson M. A. (2007). High-resolution
crystal structure of an engineered human β2-adrenergic G protein-coupled
receptor. Science.

[105] Huang W., Manglik A., Venkatakrishnan A. J., Laeremans T., Feinberg E. N., Sanborn A. L., Kato H. E., Livingston K. E., Thorsen T. S., Kling R. C. (2015). Structural
Insights into μ-Opioid Receptor Activation. Nature.

[106] Prasanna X., Mohole M., Chattopadhyay A., Sengupta D. (2020). Role of Cholesterol-Mediated
Effects in GPCR Heterodimers. Chem. Phys. Lipids.

[107] Nguyen D. H., Taub D. (2002). CXCR4 Function Requires
Membrane
Cholesterol: Implications for HIV Infection. J. Immunol..

[108] Huang P., Xu W., Yoon S. I., Chen C., Chong P. L. G., Liu-Chen L. Y. (2007). Cholesterol reduction by methyl-β-cyclodextrin
attenuates the delta opioid receptor-mediated signaling in neuronal
cells but enhances it in non-neuronal cells. Biochem. Pharmacol..

[109] Zheng H., Zou H., Liu X. (2012). Cholesterol
level influences opioid signaling in cell models and analgesia in
mice and humans. J. Lipid Res..

[110] Xu W., Yoon S. I., Huang P. (2006). Localization of the
κ Opioid Receptor in Lipid Rafts. J. Pharmacol.
Exp. Ther..

[111] Michal P., Rudajev V., El-Fakahany E. E., Doležal V. (2009). Membrane cholesterol content influences binding properties
of muscarinic M2 receptors and differentially impacts activation of
second messenger pathways. Eur. J. Pharmacol..

[112] van Aalst E., Wylie B. J. (2021). Cholesterol Is a Dose-Dependent Positive
Allosteric Modulator of CCR3 Ligand Affinity and G Protein Coupling. Front. Mol. Biosci..

[113] Hanson M. A., Cherezov V., Roth C. B. (2008). A Specific
Cholesterol Binding Site Is Established by the 2.8 Å Structure
of the Human β2-Adrenergic Receptor. Structure.

[114] Harikumar K. G., Zhao P., Cary B. P. (2024). Cholesterol-dependent
dynamic changes in the conformation of the type 1 cholecystokinin
receptor affect ligand binding and G protein coupling. PLoS Biol..

[115] Potter, R. M. ; Harikumar, K. G. ; Wu, S. V. ; Miller, L. J. Differential sensitivity of types 1 and 2 cholecystokinin receptors to membrane cholesterol. J. Lipid Res. 2012 53 137 10.1194/jlr.M020065. 22021636 PMC3243470

[116] Desai A. J., Harikumar K. G., Miller L. J. (2014). A type 1 cholecystokinin
receptor mutant that mimics the dysfunction observed for wild type
receptor in a high cholesterol environment. J. Biol. Chem..

[117] Harikumar K. G., Potter R. M., Patil A., Echeveste V., Miller L. J. (2013). Membrane Cholesterol Affects Stimulus-Activity
Coupling
in Type 1, but not Type 2, CCK Receptors: Use of Cell Lines with Elevated
Cholesterol. Lipids.

[118] Guixà-González R., Albasanz J. L., Rodriguez-Espigares I., Pastor M., Sanz F., Martí-Solano M., Manna M., Martinez-Seara H., Hildebrand P. W., Martín M., Selent J. (2017). Membrane Cholesterol
Access into
a G-Protein-Coupled Receptor. Nat. Commun..

[119] Kulig W., Tynkkynen J., Javanainen M. (2014). How well does cholesteryl hemisuccinate mimic
cholesterol in saturated
phospholipid bilayers?. J. Mol. Model..

[120] Lee A. G. (2019). Interfacial
Binding Sites for Cholesterol on G Protein-Coupled
Receptors. Biophys. J..

[121] Manna M., Niemelä M., Tynkkynen J. (2016). Mechanism of allosteric regulation of β2-adrenergic
receptor
by cholesterol. eLife.

[122] Aranda-García D., Stepniewski T. M., Torrens-Fontanals M. (2025). Large scale investigation of GPCR molecular
dynamics data uncovers
allosteric sites and lateral gateways. Nat.
Commun..

[123] Tieleman D. P., Sejdiu B. I., Cino E. A. (2021). Insights
into lipid-protein interactions from computer simulations. Biophys Rev..

[124] Marrink S. J., Risselada H. J., Yefimov S., Tieleman D. P., De Vries A. H. (2007). The MARTINI force
field: Coarse grained model for biomolecular
simulations. J. Phys. Chem. B.

[125] Souza, P. C. T. ; Alessandri, R. ; Barnoud, J. , Martini 3: a general purpose force field for coarse-grained molecular dynamics. Nat. Methods 2021 18 382 10.1038/s41592-021-01098-3. 33782607 PMC12554258

[126] Marlow B., Kuenze G., Meiler J., Koehler
Leman J. (2023). Docking cholesterol to integral membrane proteins with Rosetta. PLoS Comput. Biol..

[127] Song W., Corey R. A., Ansell T. B. (2022). PyLipID:
A Python Package for Analysis of Protein–Lipid Interactions
from Molecular Dynamics Simulations. J. Chem.
Theory Comput..

[128] Corradi V., Mendez-Villuendas E., Ingoífsson H. I., Gu R.- X., Siuda I., Melo M. N., Moussatova A., DeGagne L. J., Sejdiu B. I., Singh G. (2018). Lipid–Protein
Interactions Are Unique Fingerprints for Membrane Proteins. ACS Cent. Sci..

[129] Lihan M., Tajkhorshid E. (2024). Improved Highly Mobile Membrane Mimetic
Model for Investigating Protein–Cholesterol Interactions. J. Chem. Inf. Model..

[130] Mohole M., Naglekar A., Sengupta D., Chattopadhyay A. (2024). Probing the
energy landscape of the lipid interactions of the serotonin1A receptor. Biophys. Chem..

[131] Tzortzini E., Corey R. A., Kolocouris A. (2023). Comparative
Study of Receptor-, Receptor State-, and Membrane-Dependent Cholesterol
Binding Sites in A2A and A1 Adenosine Receptors Using Coarse-Grained
Molecular Dynamics Simulations. J. Chem. Inf.
Model..

[132] Treptow W. (2023). Allosteric Modulation of Membrane Proteins by Small
Low-Affinity Ligands. J. Chem. Inf. Model..

[133] Gupta K., Donlan J. A. C., Hopper J. T. S. (2017). The role of interfacial
lipids in stabilizing membrane protein oligomers. Nature.

[134] Gahbauer S., Pluhackova K., Böckmann R. A. (2018). Closely
related, yet unique: Distinct homo- and heterodimerization patterns
of G protein coupled chemokine receptors and their fine-tuning by
cholesterol. PLoS Comput. Biol..

[135] Pluhackova K., Gahbauer S., Kranz F., Wassenaar T. A., Böckmann R. A. (2016). Dynamic Cholesterol-Conditioned Dimerization
of the
G Protein Coupled Chemokine Receptor Type 4. PLoS Comput. Biol..

[136] Di Marino D., Conflitti P., Motta S., Limongelli V. (2023). Structural
basis of dimerization of chemokine receptors CCR5 and CXCR4. Nat. Commun..

[137] Zhao H., Lappalainen P. (2012). A simple guide to biochemical approaches
for analyzing protein–lipid interactions. Mol. Biol. Cell.

[138] Jodaitis L., van Oene T., Martens C. (2021). Assessing
the Role
of Lipids in the Molecular Mechanism of Membrane Proteins. Int. J. Mol. Sci..

[139] Bolla J. R., Agasid M. T., Mehmood S., Robinson C. V. (2019). Membrane
Protein–Lipid Interactions Probed Using Mass Spectrometry. Annu. Rev. Biochem..

[140] Kwiatek J. M., Hinde E., Gaus K. (2014). Microscopy
approaches
to investigate protein dynamics and lipid organization. Mol. Membr. Biol..

[141] Khelashvili G., Albornoz P. B. C., Johner N., Mondal S., Caffrey M., Weinstein H. (2012). Why GPCRs
behave differently in cubic
and lamellar lipidic mesophases. J. Am. Chem.
Soc..

[142] Liu W., Hanson M. A., Stevens R. C., Cherezov V. (2010). LCP-Tm: An Assay to
Measure and Understand Stability of Membrane Proteins in a Membrane
Environment. Biophys. J..

[143] Hulce J. J., Cognetta A. B., Niphakis M. J., Tully S. E., Cravatt B. F. (2013). Proteome-wide mapping of cholesterol-interacting
proteins
in mammalian cells. Nat. Methods.

[144] Budelier M. M., Cheng W. W. L., Bergdoll L. (2017). Photoaffinity
labeling with cholesterol analogues precisely maps a cholesterol-binding
site in voltage-dependent anion channel-1. J.
Biol. Chem..

[145] Grunbeck A., Huber T., Sachdev P., Sakmar T. P. (2011). Mapping
the Ligand-Binding Site on a G Protein-Coupled Receptor (GPCR) Using
Genetically Encoded Photocrosslinkers. Biochemistry.

[146] van Aalst E. J., McDonald C. J., Wylie B. J. (2023). Cholesterol Biases
the Conformational Landscape of the Chemokine Receptor CCR3: A MAS
ssNMR-Filtered Molecular Dynamics Study. J.
Chem. Inf. Model..

[147] Hu B., Höfer C. T., Thiele C., Veit M. (2019). Cholesterol
Binding to the Transmembrane Region of a Group 2 Hemagglutinin (HA)
of Influenza Virus Is Essential for Virus Replication, Affecting both
Virus Assembly and HA Fusion Activity. J. Virol..

[148] Fatakia S. N., Sarkar P., Chattopadhyay A. (2019). A collage
of cholesterol interaction motifs in the serotonin1A receptor: An
evolutionary implication for differential cholesterol interaction. Chem. Phys. Lipids.

[149] Prasanna X., Chattopadhyay A., Sengupta D. (2014). Cholesterol modulates
the dimer interface of the β2-adrenergic receptor via cholesterol
occupancy sites. Biophys. J..

[150] Epand R. M. (2008). Proteins and cholesterol-rich domains. Biochim. Biophys. Acta, Biomembr..

[151] Levitan I., Singh D. K., Rosenhouse-Dantsker A. (2014). Cholesterol
binding to ion channels. Front. Physiol..

[152] Sengupta D., Chattopadhyay A. (2012). Identification of Cholesterol Binding
Sites in the Serotonin1A Receptor. J. Phys.
Chem. B.

[153] Li H., Papadopoulos V. (1998). Peripheral-type
benzodiazepine receptor function in
cholesterol transport. Identification of a putative cholesterol recognition/interaction
amino acid sequence and consensus pattern. Endocrinology.

[154] Baier C. J., Fantini J., Barrantes F. J. (2011). Disclosure
of cholesterol recognition motifs in transmembrane domains of the
human nicotinic acetylcholine receptor. Sci.
Rep..

[155] Fantini J., Di Scala C., Evans L. S., Williamson P. T. F., Barrantes F. J. (2016). A mirror code for protein-cholesterol interactions
in the two leaflets of biological membranes. Sci. Rep..

[156] Fantini J., Barrantes F. J. (2013). How cholesterol interacts with membrane
proteins: an exploration of cholesterol-binding sites including CRAC,
CARC, and tilted domains. Front. Physiol..

[157] Nguyen A., Ondrus A. E. (2024). In Silico Tools
to Score and Predict
Cholesterol–Protein Interactions. J.
Med. Chem..

[158] Hanson, M. A. ; Cherezov, V. ; Griffith, M. T. , A Specific Cholesterol Binding Site Is Established by the 2.8 Å Structure of the Human β2-Adrenergic Receptor. Structure 2008 16 897 10.1016/j.str.2008.05.001. 18547522 PMC2601552

[159] Taghon G. J., Rowe J. B., Kapolka N. J., Isom D. G. (2021). Predictable
cholesterol binding sites in GPCRs lack consensus motifs. Structure.

[160] Ballesteros, J. A. ; Weinstein, H. [19] Integrated methods for the construction of three-dimensional models and computational probing of structure-function relations in G protein-coupled receptors. In Receptor Molecular Biology; Elsevier, 1995; Vol. 25, pp 366–428 10.1016/S1043-9471(05)80049-7.

[161] Lopez J. J., Lorch M. (2008). Location and Orientation of Serotonin
Receptor 1a Agonists in Model and Complex Lipid Membranes. J. Biol. Chem..

[162] Salas-Estrada L. A., Leioatts N., Romo T. D., Grossfield A. (2018). Lipids Alter
Rhodopsin Function via Ligand-like and Solvent-like Interactions. Biophys. J..

[163] Albert A., Boeszebattaglia K. (2005). The role of cholesterol in rod outer
segment membranes. Prog. Lipid Res..

[164] Albert A. D., Boesze-Battaglia K., Paw Z., Watts A., Epand R. M. (1996). Effect
of cholesterol on rhodopsin stability in disk
membranes. Biochim. Biophys. Acta, Protein Struct.
Mol. Enzymol..

[165] Albert A. D., Young J. E., Yeagle P. L. (1996). Rhodopsin-cholesterol
interactions in bovine rod outer segment disk membranes. Biochim. Biophys. Acta, Biomembr..

[166] Okada T., Sugihara M., Bondar A. N., Elstner M., Entel P., Buss V. (2004). The retinal conformation
and its
environment in rhodopsin in light of a new 2.2 Å crystal structure. J. Mol. Biol..

[167] Choe, H. W. ; Kim, Y. J. ; Park, J. H. , Crystal structure of metarhodopsin II. Nature 2011 471 651 10.1038/nature09789. 21389988

[168] Park, J. H. ; Scheerer, P. ; Hofmann, K. P. ; Choe, H. W. ; Ernst, O. P. Crystal structure of the ligand-free G-protein-coupled receptor opsin. Nature 2008 454 183 10.1038/nature07063. 18563085

[169] Bennett M. P., Mitchell D. C. (2008). Regulation of Membrane
Proteins by
Dietary Lipids: Effects of Cholesterol and Docosahexaenoic Acid Acyl
Chain-Containing Phospholipids on Rhodopsin Stability and Function. Biophys. J..

[170] Ruprecht J. J., Mielke T., Vogel R., Villa C., Schertler G. F. (2004). Electron crystallography reveals
the structure of metarhodopsin
I. EMBO J..

[171] Khelashvili G., Grossfield A., Feller S. E., Pitman M. C., Weinstein H. (2009). Structural
and dynamic effects of cholesterol at preferred
sites of interaction with rhodopsin identified from microsecond length
molecular dynamics simulations. Proteins: Struct.,
Funct., Bioinf..

[172] Grossfield A., Feller S. E., Pitman M. C. (2006). A role for direct
interactions in the modulation of rhodopsin by ω-3 polyunsaturated
lipids. Proc. Natl. Acad. Sci. U.S.A..

[173] Paila Y. D., Jindal E., Goswami S. K., Chattopadhyay A. (2011). Cholesterol
depletion enhances adrenergic signaling in cardiac myocytes. Biochim. Biophys. Acta Biomembr..

[174] Pontier, S. M. ; Percherancier, Y. ; Galandrin, S. ; Breit, A. ; Galés, C. ; Bouvier, M. Cholesterol-dependent separation of the β2-adrenergic receptor from its partners determines signaling efficacy: Insight into nanoscale organization of signal transduction. J. Biol. Chem.. 2008 283 24659 10.1074/jbc.M800778200. 18566454 PMC3259828

[175] MacDougall D. A., Agarwal S. R., Stopford E. A. (2012). Caveolae
compartmentalise β2-adrenoceptor signals by curtailing cAMP
production and maintaining phosphatase activity in the sarcoplasmic
reticulum of the adult ventricular myocyte. J. Mol. Cell Cardiol..

[176] Wright P. T., Nikolaev V. O., O’Hara T. (2014). Caveolin-3 regulates compartmentation of cardiomyocyte beta2-adrenergic
receptor-mediated cAMP signaling. J. Mol. Cell
Cardiol..

[177] Xiang, Y. ; Rybin, V. O. ; Steinberg, S. F. ; Kobilka, B. Caveolar localization dictates physiologic signaling of β2-adrenoceptors in neonatal cardiac myocytes. J. Biol. Chem.. 2002 277 34280 10.1074/jbc.M201644200. 12097322

[178] Xiang Y., Kobilka B. (2003). The PDZ-binding motif
of the β2
-adrenoceptor is essential for physiologic signaling and trafficking
in cardiac myocytes. Proc. Natl. Acad. Sci.
U.S.A..

[179] Scarselli M., Annibale P., Radenovic A. (2012). Cell Type-specific
β2-Adrenergic Receptor Clusters Identified Using Photoactivated
Localization Microscopy Are Not Lipid Raft Related, but Depend on
Actin Cytoskeleton Integrity. J. Biol. Chem..

[180] Sarkar P., Kumar G. A., Shrivastava S., Chattopadhyay A. (2022). Chronic cholesterol
depletion increases F-actin levels
and induces cytoskeletal reorganization via a dual mechanism. J. Lipid Res..

[181] Marheineke K., Grünewald S., Christie W., Reiländer H. (1998). Lipid composition
of *Spodoptera frugiperda* (Sf9) and
Trichoplusia ni (Tn) insect cells used for baculovirus infection. FEBS Lett..

[182] Marullo S., Delavier-Klutchko C., Eshdat Y., Strosberg A. D., Emorine L. (1988). Human beta 2-adrenergic receptors expressed in *Escherichia coli* membranes retain their pharmacological
properties. Proc. Natl. Acad. Sci. U.S.A..

[183] Geiger J., Sexton R., Al-Sahouri Z. (2021). Evidence that specific
interactions play a role in the cholesterol
sensitivity of G protein-coupled receptors. Biochim. Biophys. Acta, Biomembr..

[184] Ishchenko, A. ; Stauch, B. ; Han, G. W. , Toward G protein-coupled receptor structure-based drug design using X-ray lasers. IUCrJ 2019 6 1106 10.1107/S2052252519013137. PMC6830214 31709066

[185] Wacker D., Fenalti G., Brown M. A. (2010). Conserved
binding mode of human β2 adrenergic receptor inverse agonists
and antagonist revealed by X-ray crystallography. J. Am. Chem. Soc..

[186] Rosenbaum, D. M. ; Zhang, C. ; Lyons, J. A. , Structure and function of an irreversible agonist-β2 adrenoceptor complex. Nature 2011 469 236 10.1038/nature09665. 21228876 PMC3074335

[187] Kulig, W. ; Jurkiewicz, P. ; Olzyńska, A. , Experimental determination and computational interpretation of biophysical properties of lipid bilayers enriched by cholesteryl hemisuccinate. Biochim. Biophys. Acta Biomembr. 2015 1848 422 10.1016/j.bbamem.2014.10.032. 25450348

[188] Lan T. H., Liu Q., Li C., Wu G., Steyaert J., Lambert N. A. (2015). BRET evidence that β2 adrenergic
receptors do not oligomerize in cells. Sci.
Rep..

[189] Warne T., Serrano-Vega M. J., Baker J. G. (2008). Structure
of a β1-adrenergic G-protein-coupled receptor. Nature.

[190] Rasmussen S. G. F., DeVree B. T., Zou Y. (2011). Crystal
structure of the β2 adrenergic receptor–Gs protein complex. Nature.

[191] Cang, X. ; Yang, L. ; Yang, J. , Cholesterol-β1AR interaction versus cholesterol-β2AR interaction. Proteins: Struct., Funct., Bioinf..2014 82 760 10.1002/prot.24456. 24265091

[192] Kolokouris, D. ; Kalenderoglou, I. E. ; Duncan, A. L. ; Corey, R. A. ; Sansom, M. S. P. ; Kolocouris, A. The Role of Cholesterol in M2 Clustering and Viral Budding Explained. J. Chem. Theory Comput. 2025 21 912 10.1021/acs.jctc.4c01026. 39494590 PMC11780748

[193] Cang X., Du Y., Mao Y., Wang Y., Yang H., Jiang H. (2013). Mapping the functional binding sites
of cholesterol in β2-adrenergic receptor by long-time molecular
dynamics simulations. J. Phys. Chem. B.

[194] Gater, D. L. ; Saurel, O. ; Iordanov, I. ; Liu, W. ; Cherezov, V. ; Milon, A. Two classes of cholesterol binding sites for the β2AR revealed by thermostability and NMR. Biophys. J. 2014 107 2305 10.1016/j.bpj.2014.10.011. 25418299 PMC4241438

[195] Jafurulla M., Chattopadhyay A. (2012). Membrane lipids in the function of
serotonin and adrenergic receptors. Curr. Med.
Chem..

[196] Warne T., Edwards P. C., Doré A. S., Leslie A. G. W., Tate C. G. (2019). Molecular basis for high-affinity
agonist binding in GPCRs. Science.

[197] Warne, T. ; Moukhametzianov, R. ; Baker, J. G. , The structural basis for agonist and partial agonist action on a β1-adrenergic receptor. Nature 2011 469 241 10.1038/nature09746. 21228877 PMC3023143

[198] Abiko, L. A. ; Dias Teixeira, R. ; Engilberge, S. , Filling of a water-free void explains the allosteric regulation of the β1-adrenergic receptor by cholesterol. Nat. Chem. 2022 14 1133 10.1038/s41557-022-01009-9. 35953642

[199] Abiko L. A., Grahl A., Grzesiek S. (2019). High Pressure
Shifts
the β1 -Adrenergic Receptor to the Active Conformation in the
Absence of G Protein. J. Am. Chem. Soc..

[200] Abiko, L. A. ; Grahl, A. ; Grzesiek, S. High Pressure Shifts the β1-Adrenergic Receptor to the Active Conformation in the Absence of G Protein. J. Am. Chem. Soc. 2019 141 16663 10.1021/jacs.9b06042. 31564099

[201] Mondal S., Johnston J. M., Wang H., Khelashvili G., Filizola M., Weinstein H. (2013). Membrane driven
spatial organization
of GPCRs. Sci. Rep..

[202] Weiß H. M., Grisshammer R. (2002). Purification
and characterization
of the human adenosine A2a receptor functionally expressed in *Escherichia coli*. Eur. J. Biochem..

[203] Guixà-González R., Albasanz J. L., Rodriguez-Espigares I. (2017). Membrane cholesterol
access into a G-protein-coupled receptor. Nat.
Commun..

[204] O’Malley, M. A. ; Helgeson, M. E. ; Wagner, N. J. ; Robinson, A. S. Toward rational design of protein detergent complexes: Determinants of mixed micelles that are critical for the in vitro stabilization of a G-protein coupled receptor. Biophys. J. 2011 101 1938 10.1016/j.bpj.2011.09.018. 22004748 PMC3192985

[205] Martin-Garcia, J. M. ; Conrad, C. E. ; Nelson, G. , Serial millisecond crystallography of membrane and soluble protein microcrystals using synchrotron radiation. IUCrJ 2017 4 439 10.1107/S205225251700570X. PMC5571807 28875031

[206] Ray A. P., Thakur N., Pour N. G., Eddy M. T. (2023). Dual mechanisms
of cholesterol-GPCR interactions that depend on membrane phospholipid
composition. Structure.

[207] McGraw C., Yang L., Levental I., Lyman E., Robinson A. S. (2019). Membrane cholesterol depletion reduces
downstream signaling
activity of the adenosine A2A receptor. Biochim.
Biophys. Acta Biomembr..

[208] Huang S. K., Almurad O., Pejana R. J. (2022). Allosteric
modulation of the adenosine A2A receptor by cholesterol. eLife.

[209] Jaakola V. P., Griffith M. T., Hanson M. A. (2008). The
2.6 Angstrom Crystal Structure of a Human A2A Adenosine Receptor Bound
to an Antagonist. Science.

[210] Liu W., Chun E., Thompson A. A. (2012). Structural Basis for
Allosteric Regulation of GPCRs by Sodium Ions. Science.

[211] Liu W., Chun E., Thompson A. A. (2012). Structural basis for
allosteric regulation of GPCRs by sodium ions. Science.

[212] Melnikov I., Polovinkin V., Kovalev K. (2017). Fast iodide-SAD
phasing for high-throughput membrane protein structure determination. Sci. Adv..

[213] Batyuk A., Galli L., Ishchenko A. (2016). Native phasing of x-ray free-electron laser data for a G protein–coupled
receptor. Sci. Adv..

[214] Weinert T., Olieric N., Cheng R. (2017). Serial
millisecond crystallography for routine room-temperature structure
determination at synchrotrons. Nat. Commun..

[215] Cheng R. K. Y., Segala E., Robertson N. (2017). Structures of Human A1and A2AAdenosine Receptors with Xanthines Reveal
Determinants of Selectivity. Structure.

[216] Broecker J., Morizumi T., Ou W. L. (2018). High-throughput
in situ X-ray screening of and data collection from protein crystals
at room temperature and under cryogenic conditions. Nat. Protoc..

[217] Eddy M. T., Lee M. Y., Gao Z. G. (2018). Allosteric
Coupling of Drug Binding and Intracellular Signaling in the A2A Adenosine
Receptor. Cell.

[218] Rucktooa P., Cheng R. K. Y., Segala E. (2018). Towards
high throughput GPCR crystallography: In Meso soaking of Adenosine
A2A Receptor crystals. Sci. Rep..

[219] Segala E., Guo D., K Y., Cheng R. (2016). Controlling
the Dissociation of Ligands from the Adenosine A2A Receptor through
Modulation of Salt Bridge Strength. J. Med.
Chem..

[220] Song W., Yen H. Y., Robinson C. V., Sansom M. S. P. (2019). State-dependent
Lipid Interactions with the A2a Receptor Revealed by MD Simulations
Using In Vivo-Mimetic Membranes. Structure.

[221] Rouviere, E. ; Arnarez, C. ; Yang, L. ; Lyman, E. Identification of Two New Cholesterol Interaction Sites on the A2A Adenosine Receptor. Biophys. J. 2017 113 2415 10.1016/j.bpj.2017.09.027. 29211995 PMC5738547

[222] Lovera S., Cuzzolin A., Kelm S., De Fabritiis G., Sands Z. A. (2019). Reconstruction of apo A2A receptor
activation pathways
reveal ligand-competent intermediates and state-dependent cholesterol
hotspots. Sci. Rep..

[223] Lyman E., Higgs C., Kim B. (2009). A Role
for a Specific Cholesterol Interaction in Stabilizing the Apo Configuration
of the Human A 2A Adenosine Receptor. Structure.

[224] Lebon G., Warne T., Edwards P. C. (2011). Agonist-bound
adenosine A2A receptor structures reveal common features of GPCR activation. Nature.

[225] Magnani, F. ; Shibata, Y. ; Serrano-Vega, M. J. ; Tate, C. G. Co-evolving stability and conformational homogeneity of the human adenosine A2a receptor. Proc. Natl. Acad. Sci. U.S.A. 2008 105 10744 10.1073/pnas.0804396105. 18664584 PMC2504806

[226] Doré A., Robertson N., Errey J. C. (2011). Structure
of the adenosine A 2A receptor in complex with ZM241385 and the xanthines
XAC and caffeine. Structure.

[227] Xu F., Wu H., Katritch V. (2011). Structure of an Agonist-Bound
Human A2A Adenosine Receptor. Science.

[228] Carpenter B., Nehmé R., Warne T., Leslie A. G. W., Tate C. G. (2016). Structure
of the adenosine A2Areceptor bound to an
engineered G protein. Nature.

[229] Maria-solano M. A., Choi S. (2023). Dynamic allosteric
networks drive
adenosine A_1_ receptor activation and G-protein coupling. Biophys. J..

[230] Koretz K. S., McGraw C. E., Stradley S., Elbaradei A., Malmstadt N., Robinson A. S. (2021). Characterization
of binding kinetics
of A2AR to Gαs protein by surface plasmon resonance. Biophys. J..

[231] Thakur N., Ray A. P., Sharp L. (2023). Anionic
phospholipids control mechanisms of GPCR-G protein recognition. Nat. Commun..

[232] Ohvo H., Slotte J. P. (1996). Cyclodextrin-Mediated Removal of
Sterols from Monolayers: Effects of Sterol Structure and Phospholipids
on Desorption Rate. Biochemistry.

[233] Thakur, N. ; Ray, A. P. ; Jin, B. , Membrane mimetic-dependence of GPCR energy landscapes. Structure 2024 32 523 10.1016/j.str.2024.01.013. 38401537 PMC11069452

[234] Song W., Yen H. Y., Robinson C. V., Sansom M. S. P. (2019). State-dependent
Lipid Interactions with the A2A Receptor Revealed by MD Simulations
Using In Vivo-Mimetic Membranes. Structure.

[235] Fatakia S. N., Sarkar P., Chattopadhyay A. (2020). Molecular
evolution of a collage of cholesterol interaction motifs in transmembrane
helix V of the serotonin1A receptor. Chem. Phys.
Lipids.

[236] Pucadyil T. J., Chattopadhyay A. (2005). Cholesterol modulates the antagonist-binding
function of hippocampal serotonin1A receptors. Biochim. Biophys. Acta, Biomembr..

[237] Evans K. L. J., Cropper J. D., Berg K. A., Clarke W. P. (2001). Mechanisms
of regulation of agonist efficacy at the 5-HT­(1A) receptor by phospholipid-derived
signaling components. J. Pharmacol. Exp. Ther..

[238] Sjögren B., Csöregh L., Svenningsson P. (2008). Cholesterol
reduction attenuates 5-HT1A receptor-mediated signaling in human primary
neuronal cultures. Naunyn Schmiedebergs Arch.
Pharmacol..

[239] Sarkar, P. ; Mozumder, S. ; Bej, A. ; Mukherjee, S. ; Sengupta, J. ; Chattopadhyay, A. Structure, dynamics and lipid interactions of serotonin receptors: excitements and challenges. Biophys. Rev. 2021 13 101 10.1007/s12551-020-00772-8. PMC7930197 33188638

[240] Pucadyil T. J., Chattopadhyay A. (2004). Cholesterol modulates ligand binding
and G-protein coupling to serotonin1A receptors from bovine hippocampus. Biochim. Biophys. Acta, Biomembr..

[241] Pucadyil T. J., Chattopadhyay A. (2007). Cholesterol
depletion induces dynamic
confinement of the G-protein coupled serotonin1A receptor in the plasma
membrane of living cells. Biochim. Biophys.
Acta, Biomembr..

[242] Prasad R., Paila Y. D., Chattopadhyay A. (2009). Membrane cholesterol
depletion enhances ligand binding function of human serotonin1A receptors
in neuronal cells. Biochem. Biophys. Res. Commun..

[243] Kumar G. A., Sarkar P., Stepniewski T. M. (2021). A molecular sensor for
cholesterol in the human serotonin1A receptor. Sci. Adv..

[244] Gutierrez, M. G. ; Mansfield, K. S. ; Malmstadt, N. The Functional Activity of the Human Serotonin 5-HT1A Receptor Is Controlled by Lipid Bilayer Composition. Biophys. J. 2016 110 2486 10.1016/j.bpj.2016.04.042. 27276266 PMC4922583

[245] Shrivastava S., Pucadyil T. J., Paila Y. D., Ganguly S., Chattopadhyay A. (2010). Chronic Cholesterol Depletion Using
Statin Impairs
the Function and Dynamics of Human Serotonin 1A Receptors. Biochemistry.

[246] Chattopadhyay A., Jafurulla M., Kalipatnapu S., Pucadyil T. J., Harikumar K. G. (2005). Role of
cholesterol in ligand binding
and G-protein coupling of serotonin1A receptors solubilized from bovine
hippocampus. Biochem. Biophys. Res. Commun..

[247] Kumar G. A., Chattopadhyay A. (2021). Membrane cholesterol regulates endocytosis
and trafficking of the serotonin1A receptor: Insights from acute cholesterol
depletion. Biochim. Biophys. Acta, Mol. Cell
Biol. Lipids.

[248] Pucadyil T. J., Chattopadhyay A. (2006). Role of cholesterol in the function
and organization of G-protein coupled receptors. Prog. Lipid Res..

[249] Chattopadhyay A. (2014). GPCRs: Lipid-Dependent Membrane Receptors
That Act
as Drug Targets. Adv. Biol..

[250] Jafurulla M., Rao B. D., Sreedevi S., Ruysschaert J. M., Covey D. F., Chattopadhyay A. (2014). Stereospecific
requirement of cholesterol
in the function of the serotonin1A receptor. Biochim. Biophys. Acta, Biomembr..

[251] Sarkar P., Jafurulla Md., Bhowmick S., Chattopadhyay A. (2020). Structural
Stringency and Optimal Nature of Cholesterol Requirement in the Function
of the Serotonin1A Receptor. J. Membr. Biol..

[252] Wilson K. A., Wang L., O’Mara M. L. (2021). Site of
Cholesterol Oxidation Impacts Its Localization and Domain Formation
in the Neuronal Plasma Membrane. ACS Chem. Neurosci..

[253] Elbaradei A., Wang Z., Malmstadt N. (2022). Oxidation
of Membrane Lipids Alters the Activity of the Human Serotonin 1A Receptor. Langmuir.

[254] Sarkar P., Chattopadhyay A. (2023). Interplay
of Cholesterol and Actin
in Neurotransmitter GPCR Signaling: Insights from Chronic Cholesterol
Depletion Using Statin. ACS Chem. Neurosci..

[255] Kumar G. A., Chattopadhyay A. (2020). Statin-Induced Chronic Cholesterol
Depletion Switches GPCR Endocytosis and Trafficking: Insights from
the Serotonin 1A Receptor. ACS Chem. Neurosci..

[256] Riad M., Watkins K. C., Doucet E., Hamon M., Descarries L. (2001). Agonist-Induced
Internalization of Serotonin-1A Receptors
in the Dorsal Raphe Nucleus (Autoreceptors) But Not Hippocampus (Heteroreceptors). J. Neurosci..

[257] Bouaziz E., Emerit M. B., Vodjdani G. (2014). Neuronal
Phenotype Dependency of Agonist-Induced Internalization of the 5-HT1A
Serotonin Receptor. J. Neurosci..

[258] Scandroglio F., Venkata J. K., Loberto N. (2008). Lipid
content of brain, brain membrane lipid domains, and neurons from acid
sphingomyelinase deficient mice. J. Neurochem..

[259] Abbott S. K., Jenner A. M., Mitchell T. W., Brown S. H. J., Halliday G. M., Garner B. (2013). An Improved High-Throughput Lipid
Extraction Method for the Analysis of Human Brain Lipids. Lipids.

[260] Xu P., Huang S., Zhang H. (2021). Structural
insights
into the lipid and ligand regulation of serotonin receptors. Nature.

[261] García-Nafría, J. ; Nehmé, R. ; Edwards, P. C. ; Tate, C. G. Cryo-EM structure of the serotonin 5-HT1B receptor coupled to heterotrimeric Go. Nature 2018 558 620 10.1038/s41586-018-0241-9. 29925951 PMC6027989

[262] Ullrich A., Schneider J., Braz J. M. (2025). Discovery
of a functionally selective serotonin receptor (5-HT1AR) agonist for
the treatment of pain. Sci. Adv..

[263] Paila Y. D., Tiwari S., Sengupta D., Chattopadhyay A. (2011). Molecular
modeling of the human serotonin1A receptor: role of membrane cholesterol
in ligand binding of the receptor. Mol. Biosyst..

[264] Mohole, M. ; Prasanna, X. ; Sengupta, D. ; Chattopadhyay, A. Molecular Signatures of Cholesterol Interaction with Serotonin Receptors. In Biochemical and Biophysical Roles of Cell Surface Molecules, Advances in Experimental Medicine and Biology; Springer: Singapore, 2018; pp 151–160 10.1007/978-981-13-3065-0_11. 30637696

[265] Prasanna X., Sengupta D., Chattopadhyay A. (2016). Cholesterol-dependent
Conformational Plasticity in GPCR Dimers. Sci.
Rep..

[266] Wang C., Jiang Y., Ma J. (2013). Structural
Basis for Molecular Recognition at Serotonin Receptors. Science.

[267] Patra S. M., Chakraborty S., Shahane G. (2015). Differential
dynamics of the serotonin 1A receptor in membrane bilayers of varying
cholesterol content revealed by all atom molecular dynamics simulation. Mol. Membr. Biol..

[268] Ramírez-Anguita J. M., Rodríguez-Espigares I., Guixà-González R. (2018). Membrane cholesterol
effect on the 5-HT2A receptor: Insights into the lipid-induced modulation
of an antipsychotic drug target. Biotechnol.
Appl. Biochem..

[269] Xu P., Huang S., Zhang H. (2021). Structural insights
into the lipid and ligand regulation of serotonin receptors. Nature.

[270] Shrivastava, S. ; Jafurulla, M. ; Tiwari, S. ; Chattopadhyay, A. Identification of Sphingolipid-binding Motif in G Protein-coupled Receptors. In Biochemical and Biophysical Roles of Cell Surface Molecules; Springer: Singapore, 2018; pp 141–149 10.1007/978-981-13-3065-0_10. 30637695

[271] Gater D. L., Saurel O., Iordanov I., Liu W., Cherezov V., Milon A. (2014). Two Classes of Cholesterol Binding
Sites for the β2AR Revealed by Thermostability and NMR. Biophys. J..

[272] Bechara C., Robinson C. V. (2015). Different Modes
of Lipid Binding
to Membrane Proteins Probed by Mass Spectrometry. J. Am. Chem. Soc..

[273] Kim, K. ; Che, T. ; Panova, O. , Structure of a Hallucinogen-Activated Gq-Coupled 5-HT2A Serotonin Receptor. Cell.2020 182 1574 10.1016/j.cell.2020.08.024. 32946782 PMC7593816

[274] Cao, D. ; Yu, J. ; Wang, H. , Structure-based discovery of nonhallucinogenic psychedelic analogs. Science 2022 375 403 10.1126/science.abl8615. 35084960

[275] Shan J. F., Khelashvili G., Mondal S., Mehler E. L., Weinstein H. (2012). Ligand-dependent
conformations and dynamics of the
serotonin 5-HT2A receptor determine its activation and membrane-driven
oligomerization properties. PLoS Comput. Biol..

[276] Gimpl G., Fahrenholz F. (2002). Cholesterol
as stabilizer of the
oxytocin receptor. Biochim. Biophys. Acta, Biomembr..

[277] Klein, U. ; Gimpl, G. ; Fahrenholz, F. Alteration of the Myometrial Plasma Membrane Cholesterol Content with β-Cyclodextrin Modulates the Binding Affinity of the Oxytocin Receptor. Biochemistry 1995 34 13784 10.1021/bi00042a009. 7577971

[278] Gimpl, G. ; Klein, U. ; Reilander, H. ; Fahrenholz, F. Expression of the Human Oxytocin Receptor in Baculovirus-infected Insect Cells: High-Affinity Binding Is Induced by a Cholesterol-Cyclodextrin Complex. Biochemistry 1995 34 13794 10.1021/bi00042a010. 7577972

[279] Lemel, L. ; Nieścierowicz, K. ; García-Fernández, M. D. , The ligand-bound state of a G protein-coupled receptor stabilizes the interaction of functional cholesterol molecules. J. Lipid Res. 2021 62 100059 10.1016/j.jlr.2021.100059. 33647276 PMC8050779

[280] Mobbs J. I., Belousoff M. J., Harikumar K. G. (2021). Structures of the human
cholecystokinin 1 (CCK1) receptor bound to
Gs and Gq mimetic proteins provide insight into mechanisms of G protein
selectivity. PLoS Biol..

[281] Liu Q., Yang D., Zhuang Y. (2021). Ligand recognition and
G-protein coupling selectivity of cholecystokinin A receptor. Nat. Chem. Biol..

[282] Zhang X., He C., Wang M. (2021). Structures
of the human cholecystokinin receptors bound to agonists and antagonists. Nat. Chem. Biol..

[283] Potter R. M., Harikumar K. G., Wu S. V., Miller L. J. (2012). Differential
sensitivity of types 1 and 2 cholecystokinin receptors to membrane
cholesterol. J. Lipid Res..

[284] Zhang D., Gao Z. G., Zhang K. (2015). Two disparate
ligand-binding sites in the human P2Y1 receptor. Nature.

[285] Bari M., Battista N., Fezza F., Finazzi-Agrò A., Maccarrone M. (2005). Lipid Rafts Control Signaling of Type-1 Cannabinoid
Receptors in Neuronal Cells. J. Biol. Chem..

[286] Bari M., Spagnuolo P., Fezza F. (2006). Effect
of Lipid Rafts on Cb2 Receptor Signaling and 2-Arachidonoyl-Glycerol
Metabolism in Human Immune Cells. J. Immunol..

[287] Bari M., Paradisi A., Pasquariello N., Maccarrone M. (2005). Cholesterol-dependent
modulation of type 1 cannabinoid
receptors in nerve cells. J. Neurosci. Res..

[288] Hua T., Vemuri K., Pu M. (2016). Crystal Structure of
the Human Cannabinoid Receptor CB1. Cell.

[289] Li X., Hua T., Vemuri K. (2019). Crystal Structure of
the Human Cannabinoid Receptor CB2. Cell.

[290] Oddi, S. ; Dainese, E. ; Fezza, F. , Functional characterization of putative cholesterol binding sequence (CRAC) in human type-1 cannabinoid receptor. J. Neurochem. 2011 116 858 10.1111/j.1471-4159.2010.07041.x. 21214565

[291] Xing C., Zhuang Y., Xu T. H. (2020). Cryo-EM
Structure of the Human Cannabinoid Receptor CB2-Gi Signaling Complex. Cell.

[292] Hua T., Vemuri K., Nikas S. P. (2017). Crystal
structures of
agonist-bound human cannabinoid receptor CB1. Nature.

[293] Krishna Kumar K., Shalev-Benami M., Robertson M. J. (2019). Structure of a Signaling Cannabinoid Receptor 1-G Protein Complex. Cell.

[294] Yang X., Wang X., Xu Z. (2022). Molecular
mechanism of allosteric modulation for the cannabinoid receptor CB1. Nat. Chem. Biol..

[295] Wang X., Liu D., Shen L. (2021). A Genetically
Encoded F-19 NMR Probe Reveals the Allosteric Modulation Mechanism
of Cannabinoid Receptor 1. J. Am. Chem. Soc..

[296] Shao, Z. ; Yan, W. ; Chapman, K. , Structure of an allosteric modulator bound to the CB1 cannabinoid receptor. Nat. Chem. Biol. 2019 15 1199 10.1038/s41589-019-0387-2. 31659318

[297] Heakal Y., Woll M. P., Fox T., Seaton K., Levenson R., Kester M. (2011). Neurotensin receptor-1 inducible
palmitoylation is required for efficient receptor-mediated mitogenic-signaling
within structured membrane microdomains. Cancer
Biol. Ther..

[298] Hua, T. ; Li, X. ; Wu, L. , Activation and Signaling Mechanism Revealed by Cannabinoid Receptor-Gi Complex Structures. Cell.2020 180 655 10.1016/j.cell.2020.01.008. 32004463 PMC7898353

[299] Mystek P., Dutka P., Tworzydło M., Dziedzicka-Wasylewska M., Polit A. (2016). The role of cholesterol
and sphingolipids in the dopamine D 1 receptor and G protein distribution
in the plasma membrane. Biochim. Biophys. Acta,
Mol. Cell Biol. Lipids.

[300] Martinez V. J., Asico L. D., Jose P. A., Tiu A. C. (2020). Lipid Rafts
and Dopamine Receptor Signaling. Int. J. Mol.
Sci..

[301] Tiu A. C., Yang J., Asico L. D. (2020). Lipid
rafts are required for effective renal D1 dopamine receptor function. FASEB J..

[302] Duncan A. L., Song W., Sansom M. S. P. (2020). Lipid-Dependent
Regulation of Ion Channels and G Protein–Coupled Receptors:
Insights from Structures and Simulations. Annu.
Rev. Pharmacol. Toxicol..

[303] Yu P., Sun M., Villar V. A. M. (2014). Differential dopamine
receptor subtype regulation of adenylyl cyclases in lipid rafts in
human embryonic kidney and renal proximal tubule cells. Cell Signal..

[304] Zhuang Y., Krumm B., Zhang H. (2021). Mechanism
of dopamine binding and allosteric modulation of the human D1 dopamine
receptor. Cell Res..

[305] Heakal Y., Kester M. (2009). Nanoliposomal Short-Chain
Ceramide
Inhibits Agonist-Dependent Translocation of Neurotensin Receptor 1
to Structured Membrane Microdomains in Breast Cancer Cells. Mol. Cancer Res..

[306] TUCKER J., GRISSHAMMER R. (1996). Purification
of a rat neurotensin
receptor expressed in *Escherichia coli*. Biochem. J..

[307] Gahbauer, S. ; Böckmann, R. A. Comprehensive Characterization of Lipid-Guided G Protein-Coupled Receptor Dimerization. J. Phys. Chem. B.2020 124 2823 10.1021/acs.jpcb.0c00062. 32200641

[308] Bolivar J. H., Muñoz-García J. C., Castro-Dopico T., Dijkman P. M., Stansfeld P. J., Watts A. (2016). Interaction of lipids
with the neurotensin receptor 1. Biochim. Biophys.
Acta, Biomembr..

[309] Liu W., Jiang L., Bian C. (2016). Role of CX3CL1 in Diseases. Arch. Immunol.
Ther. Exp..

[310] Hjortø G. M., Kiilerich-Pedersen K., Selmeczi D., Kledal T. N., Larsen N. B. (2013). Human cytomegalovirus
chemokine receptor US28 induces
migration of cells on a CX3CL1-presenting surface. J. Gen. Virol..

[311] Berg C., Rosenkilde M. M. (2023). Therapeutic
targeting of HCMV-encoded
chemokine receptor US28: Progress and challenges. Front. Immunol..

[312] Lu M., Zhao W., Han S. (2022). Activation of the human
chemokine receptor CX3CR1 regulated by cholesterol. Sci. Adv..

[313] Oakes V., Domene C. (2019). Influence of Cholesterol and Its
Stereoisomers on Members of the Serotonin Receptor Family. J. Mol. Biol..

[314] Jakubík J., El-Fakahany E. E. (2021). Allosteric
Modulation of GPCRs of
Class A by Cholesterol. Int. J. Mol. Sci..

[315] Kiriakidi S., Kolocouris A., Liapakis G., Ikram S., Durdagi S., Mavromoustakos T. (2019). Effects of
Cholesterol on GPCR Function:
Insights from Computational and Experimental Studies. Adv. Exp. Med. Biol..

[316] Baccouch R., Rascol E., Stoklosa K., Alves I. D. (2022). The role
of the lipid environment in the activity of G protein coupled receptors. Biophys. Chem..

[317] Isu U. H., Badiee S. A., Khodadadi E., Moradi M. (2023). Cholesterol in Class C GPCRs: Role, Relevance, and
Localization. Membranes.

[318] Weston C., Poyner D., Patel V., Dowell S., Ladds G. (2014). Investigating G protein signalling
bias at the glucagon-like peptide-1
receptor in yeast. Br. J. Pharmacol..

[319] Marino K.
A., Prada-Gracia D., Provasi D., Filizola M. (2016). Impact of
Lipid Composition and Receptor Conformation on the Spatio-temporal
Organization of μ-Opioid Receptors in a Multi-component Plasma
Membrane Model. PLoS Comput. Biol..

[320] Jones A. J. Y., Gabriel F., Tandale A., Nietlispach D. (2020). Structure
and Dynamics of GPCRs in Lipid Membranes: Physical Principles and
Experimental Approaches. Molecules.

[321] Chipot C., Dehez F., Schnell J. R. (2018). Perturbations
of Native Membrane Protein Structure in Alkyl Phosphocholine Detergents:
A Critical Assessment of NMR and Biophysical Studies. Chem. Rev..

[322] Mizumura T., Kondo K., Kurita M. (2020). Activation
of adenosine A2A receptor by lipids from docosahexaenoic acid revealed
by NMR. Sci. Adv..

[323] Denisov I. G., Sligar S. G. (2016). Nanodiscs for structural and functional
studies of membrane proteins. Nat. Struct. Mol.
Biol..

[324] Ren Q., Zhang S., Bao H. (2022). Circularized fluorescent nanodiscs
for probing protein–lipid interactions. Commun. Biol..

[325] Borges-Araújo L., Borges-Araújo A. C., Ozturk T. N. (2023). Martini 3 Coarse-Grained Force Field for Cholesterol. J. Chem. Theory Comput..

[326] Carpenter B., Nehmé R., Warne T., Leslie A. G. W., Tate C. G. (2016). Structure of the
adenosine A2A receptor bound to an
engineered G protein. Nature.

[327] Lu, J. ; Byrne, N. ; Wang, J. , Structural basis for the cooperative allosteric activation of the free fatty acid receptor GPR40. Nat. Struct. Mol. Biol. 2017 24 570 10.1038/nsmb.3417. 28581512

[328] Xiao, P. ; Yan, W. ; Gou, L. , Ligand recognition and allosteric regulation of DRD1-Gs signaling complexes. Cell 2021 184 943 10.1016/j.cell.2021.01.028. 33571432 PMC11005940

[329] Liu, X. ; Kaindl, J. ; Korczynska, M. , An allosteric modulator binds to a conformational hub in the β2 adrenergic receptor. Nat. Chem. Biol. 2020 16 749 10.1038/s41589-020-0549-2. 32483378 PMC7816728

[330] Obi P., Natesan S. (2022). Membrane Lipids Are
an Integral Part of Transmembrane
Allosteric Sites in GPCRs: A Case Study of Cannabinoid CB1 Receptor
Bound to a Negative Allosteric Modulator, ORG27569, and Analogs. J. Med. Chem..

[331] Hedderich J. B., Persechino M., Becker K. (2022). The pocketome
of G-protein-coupled receptors reveals previously untargeted allosteric
sites. Nat. Commun..

[332] Heydenreich F. M., Marti-Solano M., Sandhu M., Kobilka B. K., Bouvier M., Babu M. M. (2023). Molecular
determinants of ligand
efficacy and potency in GPCR signaling. Science.

[333] Wakefield A. E., Bajusz D., Kozakov D., Keserű G. M., Vajda S. (2022). Conservation of Allosteric Ligand
Binding Sites in G-Protein Coupled
Receptors. J. Chem. Inf. Model..

[334] Réffy B., Kolonits M., Schulz A., Klapötke T. M., Hargittai M. (2000). Intriguing Gold TrifluorideMolecular Structure of Monomers
and Dimers: An Electron Diffraction and Quantum Chemical Study. J. Am. Chem. Soc..

[335] Teng X., Chen S., Nie Y. (2022). Ligand
recognition and biased agonism of the D1 dopamine receptor. Nat. Commun..

[336] Barekatain M., Johansson L. C., Lam J. H. (2024). Structural
insights into the high basal activity and inverse agonism of the orphan
receptor GPR6 implicated in Parkinson’s disease. Sci. Signal..

[337] Oeckl P., Hengerer B., Ferger B. (2014). G-protein coupled receptor
6 deficiency alters striatal dopamine and cAMP concentrations and
reduces dyskinesia in a mouse model of Parkinson’s disease. Exp. Neurol..

