# Supplementary material for: Effects of Membrane Cholesterol on the Structure and Function of Selected Class A GPCRs—Challenges and Future Perspectives
Source: Biochemistry. 2025 Aug 8;64(19):4011–49. doi: 10.1021/acs.biochem.5c00145 (PMC12509330; doi:10.1021/acs.biochem.5c00145)
Supplement: Supplementary file 1 [file bi5c00145_si_001.pdf]

Supporting Information

Effects of Membrane Cholesterol on the Structure and Function of Selected Class A GPCRs – Challenges and Future Perspectives

Marina Christofidi, [1,x] EfpraxiaTzortzini, [1,x] Thomas Mavromoustakos, [2] Antonios Kolocouris [1,*]

[1] Laboratory of Medicinal Chemistry, Section of Pharmaceutical Chemistry, Department of Pharmacy, School of Health Sciences, National and Kapodistrian University of Athens, Panepistimiopolis-Zografou, 15771 Athens, Greece

[2] Laboratory of Organic Chemistry, Department of Chemistry, School of Science, National and Kapodistrian University of Athens, Panepistimiopolis-Zografou, 15771 Athens, Greece

x: these authors contribute equally

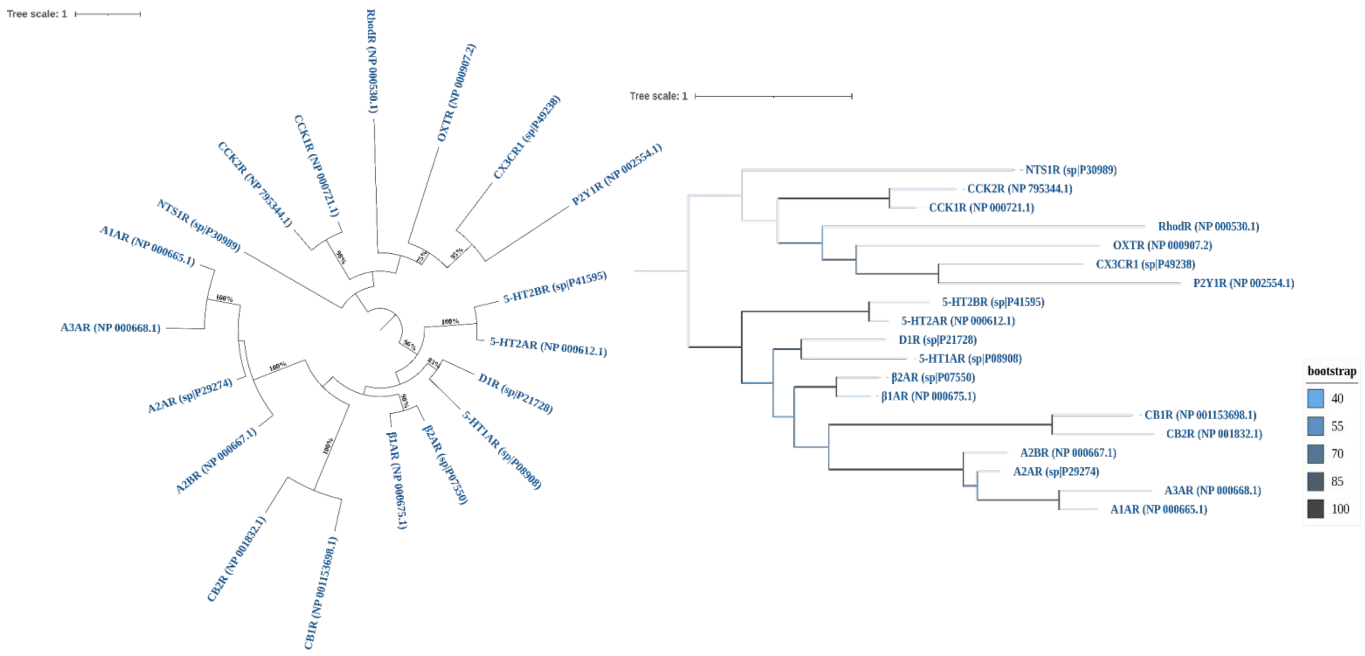

Figure S1. Phylogenetic tree of the receptors NTS1R(P30989), RhoR (NP_00530.1), CX3CR1(P49238), D1R (P21728), β_1AR (NP_000675.1), β_2AR (P07550), A_{2B}R (NP_000667.1), A_1R (NP_000665.1), A_2R (P29274), A_3R (NP_00668.1), 5-HT_{1A}R (P08908), 5-HT_{2A}R (NP_000612.1), 5-HT_{2B}R (P41595), OXTR

(NP_000907.2), CCK_1R (NP_000721.1), CCK_2R (NP795344.1), P2Y1R (NP_002554.1), CB_1R (NP_001153698.1), CB_2R (NP_001832.1) were produced based on the multiple sequence alignment (see **Figure S2**), using the IQ-TREE2 tool, [1] and visualised by the iTOL software. [2] Phylogenetic analysis and tree visualization were conducted using IQ-TREE [1] and the Interactive Tree of Life (iTOL) platform. [2] IQ-TREE (v2) was used to construct maximum likelihood phylogenetic trees, with model selection and branch support assessed via the ultrafast bootstrap approximation (UFBoot2, 1000 replicates), a statistically efficient method for evaluating clade reliability. [1] Bootstrap values range from 0% to 100% and represent the proportion of resampled datasets that support a given branch. Values ≥ 90% indicate very strong support (high-confidence), 70–89% suggest moderately strong support, and values below 70% are generally considered less reliable for phylogenetic inference and should be interpreted with caution, as they may represent less robust evolutionary relationships. The resulting tree files were visualized using iTOL (v6), an online platform for interactive tree rendering. [1] Bootstrap support values were incorporated into the tree as scaled node labels to visually emphasize clade confidence, thereby aiding in the interpretation of receptor clustering patterns.

A.

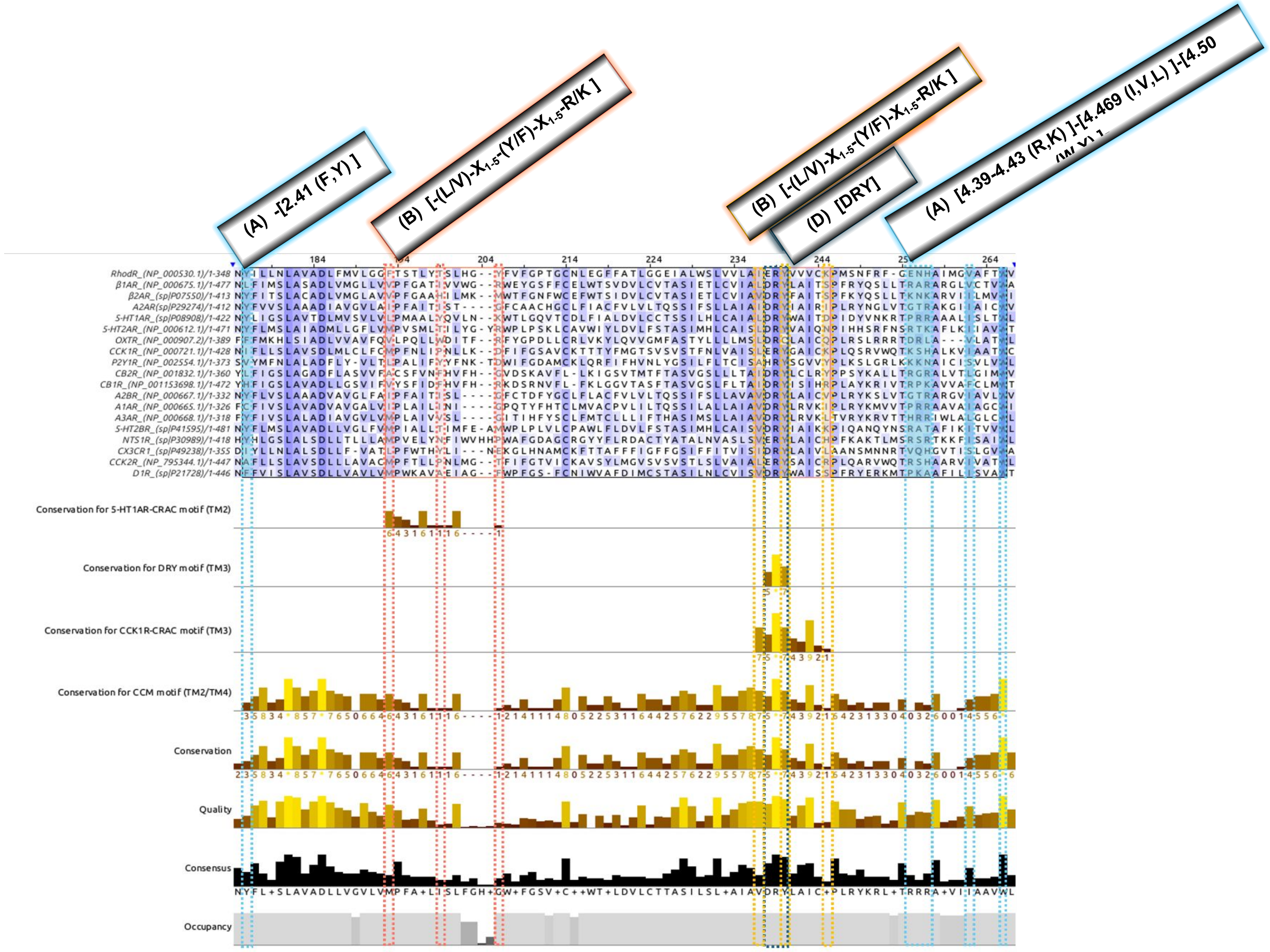

(A) -[2.41 (F,Y)]
(B) [-(L/V)-X1-5-(Y/F)-X1-5-R/K]
(B) [-(L/V)-X1-5-(Y/F)-X1-5-R/K]
(D) [DRY]
(A) [4.39-4.43 (R,K)]-[4.469 (I,V,L)]-[4.50
184
204
214
224
234
244
264
RhodR_(NP_000530.1)/1-348
β1AR_(NP_000675.1)/1-477
β2AR_(sp|P07550)/1-413
A2AR(sp|P29274)/1-412
5-HT1AR_(sp|P08908)/1-422
5-HT2AR_(NP_000612.1)/1-471
OXTR_(NP_000907.2)/1-389
CCK1R_(NP_000721.1)/1-428
P2Y1R_(NP_002554.1)/1-373
CB2R_(NP_001832.1)/1-360
CB1R_(NP_001153698.1)/1-472
A2BR_(NP_000667.1)/1-332
A1AR_(NP_000665.1)/1-326
A3AR_(NP_000668.1)/1-318
5-HT2BR_(sp|P41595)/1-481
NTS1R_(sp|P30989)/1-418
CX3CR1_(sp|P49238)/1-355
CCK2R_(NP_795344.1)/1-447
D1R_(sp|P21728)/1-446
Conservation for 5-HT1AR-CRAC motif (TM2)
Conservation for DRY motif (TM3)
Conservation for CCK1R-CRAC motif (TM3)
Conservation for CCM motif (TM2/TM4)
Conservation
Quality
Consensus
Occupancy

B.

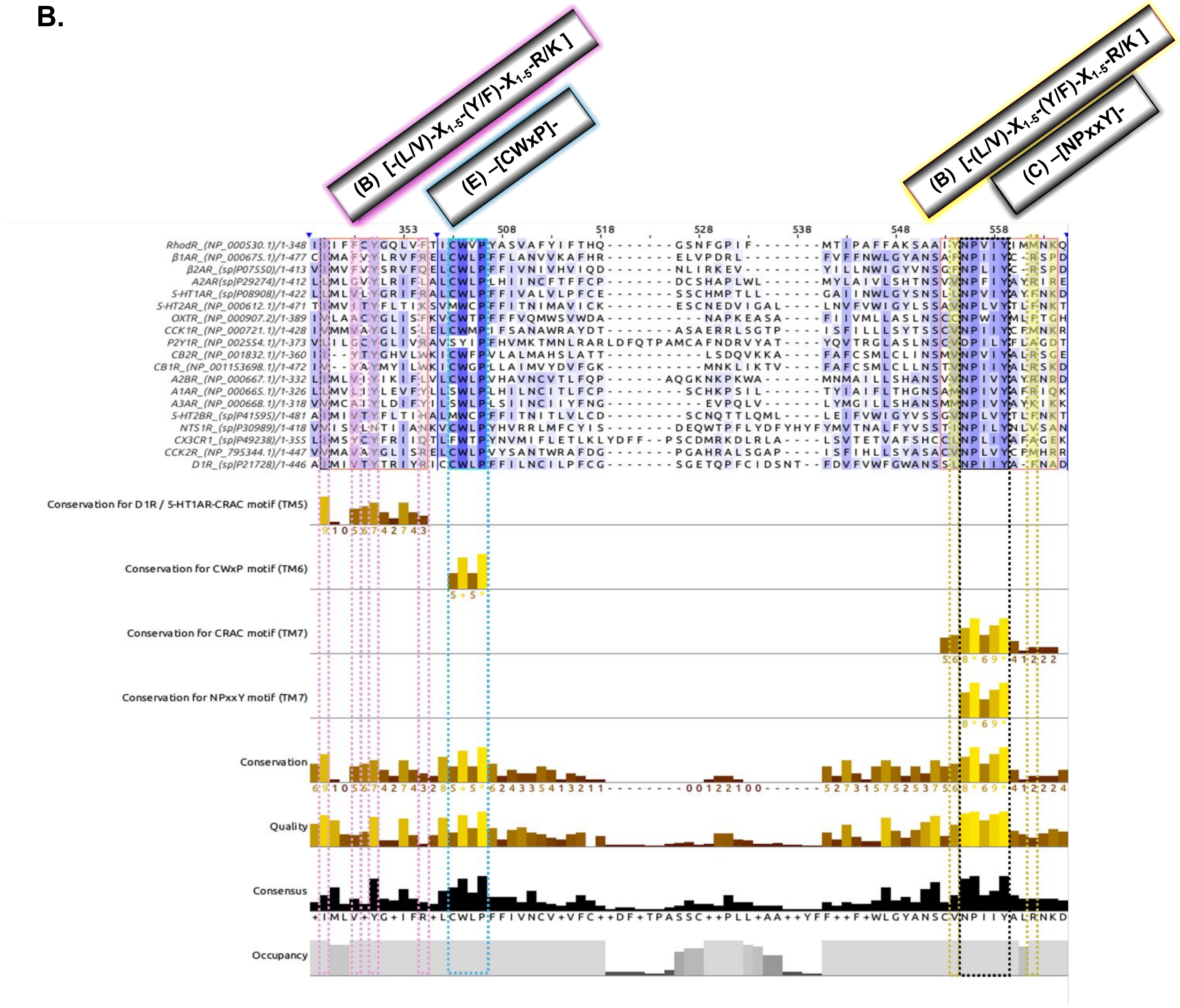

Figure S2. Multiple sequence analysis, using MAFFT tool. The conservation between the receptors NTS1R(P30989), RhoR (NP_00530.1), CX3CR1(P49238), D$_1$R (P21728), β$_1$AR (NP_000675.1), β$_2$AR (P07550), A$_{2B}$R (NP_000667.1), A$_1$R (NP_000665.1), A$_{2A}$R (P29274), A$_3$R (NP_00668.1), 5-HT$_{1A}$R (P08908), 5-HT$_{2A}$R (NP_000612.1), 5-HT$_{2B}$R (P41595), OXTR (NP_000907.2), CCK$_1$R (NP_000721.1), CCK$_2$R (NP795344.1), P2Y1R (NP_002554.1), CB$_1$R (NP_001153698.1), CB$_2$R (NP_001832.1) is shown.
A. MSA with significant motifs in TM2-4 (CCM, CRAC, DRY)
B. MSA with significant motifs in TM5-H8 (CRAC, CWxP,NPxxY)

(A) **CCM motif:** [4.39-4.43 (R,K)]-[4.469 (I,V,L)]-[4.50 (W,Y)]-[2.41 (F,Y)] (Significant Residues: Light Blue highlights)
(B) **CRAC motif:** [-(L/V)-X$_{1-5}$-(Y/F)-X$_{1-5}$-R/K] (Significant residues: TM2: Orange, TM3: Light Orange, TM5: Pink, TM7: Yellow highlights)
(C) **NPxxY motif:** It is collocated with CRAC motif in TM7 (Black dashed lines)
(D) **DRY motif:** (Grey dashed lines)
(E) **CWxP motif:** (Light blue dashed lines)
The sequence is coloured by percentage identity Low percentage: White -> High percentage: Dark blue & Black sequence of the residues between the receptors. Multiple sequence alignments were performed using MAFFT v7.505 [3] and were further inspected and manually annotated in Jalview v2.11.3.2. [4]
The phylogenetic relationship between the receptors reveals similarities and variations in cholesterol interaction patterns, which are also evolutionarily related, as the motifs that play a main role in receptor-cholesterol interactions are highly conserved, e.g., **CCM** or **CRAC** motifs.

Table S1. Comparison of predicted conserved **CCM** and **CRAC** motifs based only on the sequence conservation.

**Receptor**	**CCM**		**CRAC**	
β$_1$AR	R174$^{4.41}$ -V179$^{4.46}$- W183$^{4.50}$- -$^{2.41}$	TM4	-	-
β$_2$AR	K149$^{4.41}$ -I154$^{4.46}$- W158$^{4.50}$ -Y70$^{2.41}$	IC TM2-TM4	L324$^{7.51}$-X$^{7.52}$-Y326$^{7.53}$-X$^{7.54}$-R328$^{7.55}$	IC TM7
5-HT$_1$AR	R152$^{4.41}$ -I157$^{4.46}$- W161$^{4.50}$ -Y73$^{2.41}$	IC TM2-TM4	L395$^{7.48}$-(X$^{7.49}$-X$^{7.52}$)-Y400$^{7.53}$-(X$^{7.54}$-X$^{8.47}$)-K405$^{8.48}$	IC TM7/H8
			L90$^{2.57}$-(X$^{2.58}$-X$^{2.62}$)-Y96$^{2.63}$-(X$^{2.64}$-X$^{2.67}$)-K101^{EL1}	EC TM2
			L210$^{5.53}$-(X$^{5.54}$-X$^{5.57}$)-Y215$^{5.58}$-(X$^{5.59}$-X$^{5.62}$)-R220$^{5.63}$	IC TM5
5-HT$_{2A}$R	K191$^{4.41}$-I196$^{4.46}$-W200$^{4.50}$-Y111$^{2.41}$	IC TM2-TM4	V375$^{7.48}$-(X$^{7.49}$-X$^{7.52}$)-Y380$^{7.53}$-(X$^{7.54}$-X$^{8.47}$)-K385$^{8.48}$	IC TM7/H8
5-HT$_{2B}$R	R169$^{4.39}$-I176$^{4.46}$-W180$^{4.50}$-Y91$^{2.41}$	IC TM2-TM4	V375$^{7.48}$-(X$^{7.49}$-X$^{7.52}$)-Y380$^{7.53}$-(X$^{7.54}$-X$^{8.47}$)-K385$^{8.48}$	IC TM7/H8
A$_{2A}$R	R120$^{4.41}$-I125$^{4.46}$-W129$^{4.50}$-Y43$^{2.41}$	IC TM2-TM4	V282$^{7.47}$-(X$^{7.48}$-X$^{7.50}$)-F286$^{7.51}$-(X$^{7.52}$-X$^{7.55}$)-R293$^{7.58}$	IC TM7
A$_{2B}$R	R128$^{4.41}$-I133$^{4.46}$-W137$^{4.50}$- -$^{2.41}$	IC TM4	V424$^{7.51}$-(X$^{7.52}$ -X$^{7.56}$)-F429$^{7.56}$-(X$^{7.57}$-X$^{8.50}$)-R434$^{8.51}$	IC TM7/H8
A$_1$R	R142$^{4.41}$-I147$^{4.46}$-W151$^{4.50}$-Y63$^{2.41}$	IC TM2-TM4	-	-
A$_3$R	R126$^{4.41}$-L131$^{4.46}$-W135$^{4.50}$-Y49$^{2.41}$	IC TM2-TM4	V281$^{7.52}$-(X$^{7.43}$)-Y284$^{7.55}$-(X$^{7.54}$-X$^{8.47}$)- K287$^{8.48}$	IC TM7/H8
A$_{2B}$R	R128$^{4.41}$-I133$^{4.46}$-W137$^{4.50}$- -$^{2.41}$	IC TM4	V424$^{7.51}$-(X$^{7.52}$ -X$^{7.56}$)-F429$^{7.56}$-(X$^{7.57}$-X$^{8.50}$)-R434$^{8.51}$	IC TM7/H8
CB$_1$R	K232$^{4.41}$- -$^{4.46}$-W241$^{4.50}$- -$^{2.41}$	IC TM4	V392$^{7.48}$-(X$^{7.49}$-X$^{7.52}$)-Y397$^{7.53}$-(X$^{7.54}$-X$^{8.47}$)-K402$^{8.48}$	IC TM7/H8
CB$_2$R	R149$^{4.41}$-L154$^{4.46}$-W148$^{4.50}$- -$^{2.41}$	IC TM4	V294$^{7.48}$-(X$^{7.49}$-X$^{7.52}$)-Y299$^{7.53}$-(X$^{7.54}$-X$^{7.55}$)- R302$^{7.56}$	IC TM7
CCK1R	K155$^{4.39}$-I162$^{4.46}$-W166$^{4.50}$- -$^{2.41}$	IC TM4	V365$^{7.48}$-(X$^{7.49}$-X$^{7.52}$)-Y370$^{7.53}$-(X$^{7.54}$-X$^{8.47}$)-K375$^{8.48}$	IC TM7/H8
			L137$^{3.48}$-(X$^{3.49}$-X$^{3.50}$)-Y140$^{3.51}$-(X$^{3.52}$-X$^{3.55}$)-K145$^{3.56}$	IC TM3
CCK2R	K168$^{4.39}$-I175$^{4.46}$-W179$^{4.50}$- S95$^{2.41}$	IC TM2-TM4	V385$^{7.48}$-(X$^{7.49}$-X$^{7.52}$)-Y390$^{7.53}$-(X$^{7.54}$-X$^{8.47}$)-R395$^{8.48}$	IC TM7/H8
RhoR	- $^{4.41}$-V157$^{4.46}$-W161$^{4.50}$-Y74$^{2.41}$	IC TM2-TM4	V304$^{7.51}$-X$^{7.52}$-Y306$^{7.53}$-(X$^{7.54}$-X$^{8.47}$)-K311$^{8.48}$	IC TM7/H8
P2Y1R	K166$^{4.40}$- -$^{4.46}$-W176$^{4.50}$- -$^{2.41}$	TM4	-	-
OXTR	R151$^{4.41}$-V157$^{4.46}$-W161$^{4.50}$-F76$^{2.41}$	IC TM2-TM4	-	-
NTS1R	R182$^{4.34}$- I189$^{4.46}$-W193$^{4.50}$- Y103$^{2.41}$	IC TM2-TM4	V367$^{7.56}$-(X$^{7.57}$-X$^{8.49}$)-F371$^{8.50}$-K372$^{8.51}$	IC TM7/H8
D1R	K138$^{4.40}$-I144$^{4.46}$-W148$^{4.50}$- F61$^{2.41}$	IC TM2-TM4	V212$^{5.56}$-(X$^{5.57}$-X$^{5.61}$)-Y218$^{5.62}$-R219$^{5.63}$	IC TM5
CX3CR1	-$^{4.41}$- -$^{4.46}$-W176$^{4.50}$- -$^{2.41}$	IC TM2-TM4	L288$^{7.48}$-(X$^{7.49}$-X$^{7.52}$)-Y293$^{7.53}$-(X$^{7.54}$-X$^{8.48}$)-K299$^{8.49}$	IC TM7/H8

Methods: Phylogenetic and Sequence Analysis

Sequence Retrieval and Alignment
FASTA-formatted amino acid sequences of selected Class A G protein-coupled receptors (GPCRs) were retrieved from the NCBI RefSeq and UniProt databases. Multiple sequence alignment was performed using MAFFT v7.505[3] with the L-INS-i strategy (--localpair --maxiterate 1000) to ensure high alignment accuracy in conserved transmembrane and motif regions.

Alignment Visualization
The resulting multiple sequence alignment (MSA) was visualized using Jalview v2.11.3.2[4] . Sequence conservation across the GPCR family was assessed using Jalview's built-in percent identity score, which was applied to color residues based on their conservation across all aligned sequences. Highly conserved residues appear in darker shades, while variable positions are lighter. In addition, key GPCR functional motifs—including DRY (TM3), CWxP (TM6), and NPxxY (TM7), CRAC and CCM motifs—were manually highlighted and annotated within the alignment to facilitate structural and functional interpretation.

Phylogenetic Analysis
To infer evolutionary relationships among the 19 GPCR sequences, the alignment was first trimmed using trimAl v1.4.rev22[5] with the -automated1 flag to remove poorly aligned or gapped regions. The cleaned alignment was used as input for IQ-TREE2 v2.2.2.6 [1], a maximum-likelihood phylogenetic inference tool. The best-fit evolutionary model was determined using ModelFinder Plus (MFP), and node support was evaluated with 1,000 ultrafast bootstrap replicates (-bb 1000) and 1,000 SH-aLRT replicates (-alrt 1000). Computation was performed using automatic multithreading (-nt AUTO). The resulting phylogenetic tree was visualized using Interactive Tree of Life iTOL v6 [2] (https://itol.embl.de) and annotated to highlight receptor subfamilies.

Evolutionary Conservation Mapping
Conservation scores were calculated using the ConSurf 2020 server [6] (https://consurf.tau.ac.il/). The predicted 3D structure of the human β2-adrenergic receptor (β2AR; UniProt ID: P07550) was obtained from the AlphaFold Protein Structure Database (accessed via https://alphafold.ebi.ac.uk/entry/P07550) and used as the input structure for ConSurf analysis to map the evolutionary conservation of residues across GPCR Class A receptors. The MAFFT alignment and the structure of the human β1-adrenergic receptor were submitted. Finally, the analysis employed the Bayesian method for estimating conservation scores, which provides posterior probabilities for the evolutionary rate at each position. The amino acid substitution model was selected automatically by ConSurf based on best-fit criteria, ensuring the optimal evolutionary model was used for the dataset. Conservation scores were normalized on a scale from 1 (variable) to 9 (highly conserved) and mapped to the B-factor column of the reference structure (Uniprot ID: P075570) for visualization in PyMOL. Residues with insufficient coverage or low confidence were excluded from scoring and displayed in neutral gray.

Structure Visualization
Structural rendering and analysis were performed using PyMOL v3.1.4.1. [7] The structure of the human β1-adrenergic receptor (UniprotID: P07550) was displayed in cartoon representation. Conservation scores from ConSurf [6] were mapped onto the structure using the B-factor field and visualized using the color scale same as above. All images were exported as high-resolution PNG files (300 dpi).

References

1. Minh BQ, Schmidt HA, Chernomor O, et al. IQ-TREE 2: New Models and Efficient Methods for Phylogenetic Inference in the Genomic Era. *Mol Biol Evol*. 2020;37(5):1530-1534. doi:10.1093/molbev/msaa015
2. Letunic I, Bork P. Interactive Tree of Life (iTOL) v6: recent updates to the phylogenetic tree display and annotation tool. *Nucleic Acids Res*. 2024;52(W1):W78-W82. doi:10.1093/nar/gkae268
3. Katoh K, Standley DM. MAFFT Multiple Sequence Alignment Software Version 7: Improvements in Performance and Usability. *Mol Biol Evol*. 2013;30(4):772-780. doi:10.1093/molbev/mst010
4. Waterhouse AM, Procter JB, Martin DMA, Clamp M, Barton GJ. Jalview Version 2—a multiple sequence alignment editor and analysis workbench. *Bioinformatics*. 2009;25(9):1189-1191. doi:10.1093/bioinformatics/btp033
5. Capella-Gutiérrez S, Silla-Martínez JM, Gabaldón T. trimAl: a tool for automated alignment trimming in large-scale phylogenetic analyses. *Bioinformatics*. 2009;25(15):1972-1973. doi:10.1093/bioinformatics/btp348
6. Ashkenazy H, Abadi S, Martz E, et al. ConSurf 2016: an improved methodology to estimate and visualize evolutionary conservation in macromolecules. *Nucleic Acids Res*. 2016;44(W1):W344-W350. doi:10.1093/nar/gkw408
7. Schrödinger L. The PyMOL Molecular Graphics System, Version 3.1.4.1. *New York, NY*. Published online 2023.
8. Hanson MA, Cherezov V, Roth CB, et al. A specific cholesterol binding site is established by the 2.8 Å. *Structure*. 2009;16(6):897-905. doi:10.1016/j.str.2008.05.001.A
